# Machine learning-assisted molecular design for efficient ^19^F hyperpolarization

**DOI:** 10.1039/d6sc04732g

**Published:** 2026-08-27

**Authors:** Qiwei Peng, Li Zheng, Huijun Sun, Yubin Xiong, Zeyu Zheng, Xiaohong Cui, Zhong Chen, Xinchang Wang

**Affiliations:** a School of Electronic Science and Engineering, State Key Laboratory of Physical Chemistry of Solid Surfaces, Xiamen University Xiamen China xcwang@xmu.edu.cn

## Abstract

Efficient ^19^F signal amplification by reversible exchange (SABRE) remains difficult because enhancement depends on multiple substrate-specific structural and electronic factors. Here we report a machine-learning-assisted strategy for identifying and ranking high-performance ^19^F SABRE substrates. A chemical space of more than 180 000 fluorinated N-heterocycles was mapped using refined molecular descriptors, and 33 representative fluorinated pyridine substrates were selected for experimental SABRE screening. Random forest classification identified N_Sum_of_connectivity as a key descriptor separating high- and low-enhancement substrates. For substrates classified in the high-enhancement regime, forward stepwise multivariate linear regression with leave-one-out cross-validation produced an interpretable five-descriptor model for quantitative signal-enhancement-factor prediction. External validation with six additional substrates showed good agreement between predicted and experimental values for high-enhancement candidates. Notably, 3-amino-4-fluoropyridine gave an experimental enhancement of 7133-fold, close to the predicted value of 6637-fold, corresponding to approximately 3.2% polarization at 1.4 T without co-ligands. The selected substrates further enabled single-scan simultaneous hyperpolarization of 15 fluorinated pyridines. This workflow provides an interpretable framework for data-driven optimization of ^19^F SABRE substrates.

## Abstract 2

An interpretable ML model quantitatively predicts ^19^F SABRE enhancements beyond training. Validation showed a maximum 7133-fold enhancement without co-ligands; the substrates enabled single-scan multiplexed hyperpolarization of 15 fluoropyridines.

## Graphical abstract

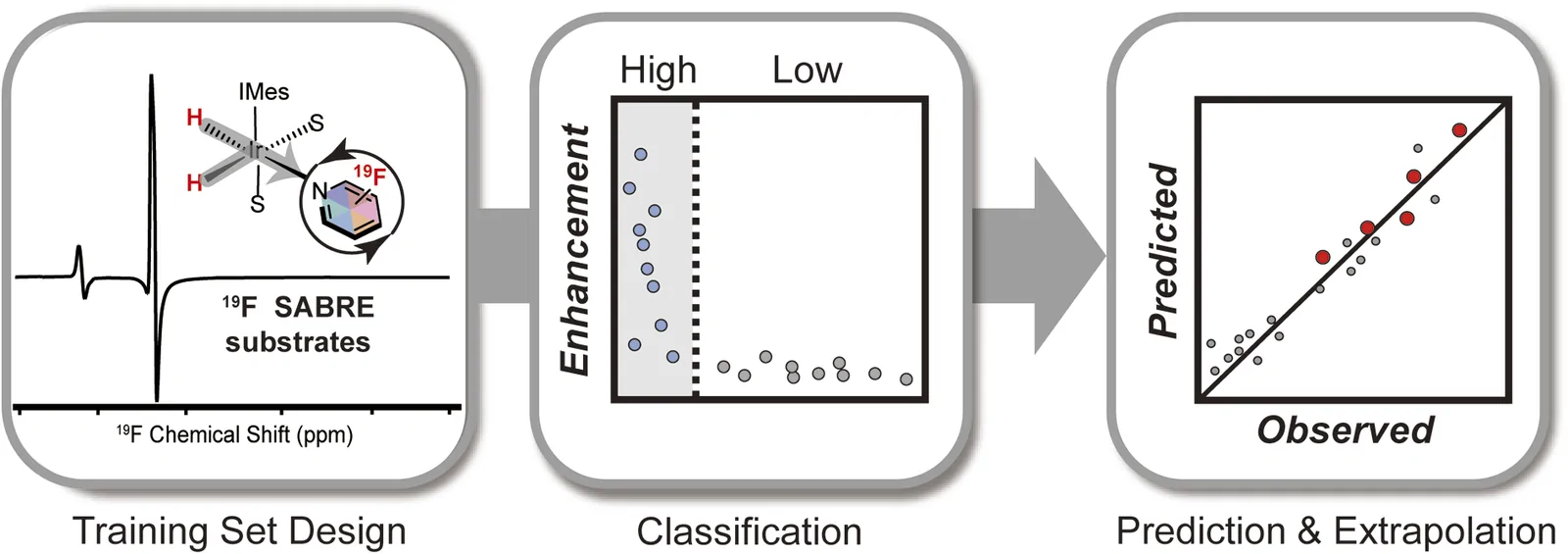

## Introduction

Fluorine-19 nuclear magnetic resonance (^19^F NMR) has emerged as an important and versatile analytical technique for chemical and biomedical research.^1^ Its utility arises from a combination of favorable nuclear properties, including a gyromagnetic ratio comparable to that of ^1^H, nearly 100% natural abundance, and the negligible endogenous background in biological systems, which together enable background-free detection. In addition, ^19^F exhibits a wide chemical shift range of more than 400 ppm and high sensitivity to the local chemical environment. Therefore, ^19^F-labeled compounds have been widely used as molecular probes and contrast agents in magnetic-resonance-related fields.^2–4^

Despite these advantages, the inherently low sensitivity of ^19^F NMR remains a major barrier to its broader application, particularly in rapid and high-sensitivity detection scenarios. Hyperpolarization techniques enhance NMR sensitivity by increasing the population difference between nuclear spin energy levels, and methods such as dynamic nuclear polarization (DNP),^5–9^ chemically induced dynamic nuclear polarization (CIDNP),^10–12^ and parahydrogen-based polarization,^13–15^ have been widely applied in chemical and biological research. Among these approaches, signal amplification by reversible exchange (SABRE)^16^ is especially attractive because it operates under mild conditions, provides high polarization efficiency, and allows repeated polarization cycles. In SABRE, both the substrate and parahydrogen reversibly bind to an iridium-based catalyst to form a metal complex, within which parahydrogen-derived spin order is spontaneously transferred to the substrate, thereby enhancing the sensitivity of other nuclei.

Using SABRE, high polarization levels have been achieved for ^1^H, ^13^C, and ^15^N,^17–24^ whereas the polarization level of ^19^F remains relatively low. One important reason is that the range of fluorinated substrates explored for ^19^F SABRE remains relatively limited. Previous studies have largely focused on optimizing the hyperpolarization performance of a small number of well-studied model substrates.^25–32^ Although these reports have provided valuable mechanistic insights, broader and more systematic investigations across larger molecular libraries remain rare.

Fluorinated N-heterocyclic compounds represent an important class of substrates for ^19^F SABRE hyperpolarization, and their enhancement factors depend on multiple intrinsic structural features. For example, the electronic properties of substituents on the pyridine ring, such as electron-withdrawing or electron-donating effects,^33^ as well as steric factors,^28^ can directly or indirectly modulate the efficiency of reversible exchange processes and the dynamics of polarization transfer, thereby influencing the final ^19^F NMR signal enhancement. Achieving higher ^19^F signal enhancement requires the simultaneous optimization of multiple, often interdependent molecular parameters, making this a high-dimensional optimization problem in chemical space. Exploring such a space by conventional trial-and-error strategies would require extensive experimental screening, resulting in high material consumption, long experimental timelines, and substantial cost.

In this context, data science and machine learning offer powerful tools for addressing this challenge.^34^ We hypothesized that, by modeling datasets linking the molecular descriptors of fluorinated N-heterocyclic compounds, such as electronic and steric parameters, to their corresponding ^19^F SABRE signal enhancement factors (SEFs), machine learning could uncover the hidden structure–property relationships in high-dimensional and nonlinear data and thereby enable quantitative assessment of how different structural features contribute to hyperpolarization efficiency.

Herein, we report a chemical-space-based machine-learning workflow for ^19^F SABRE hyperpolarization, aimed at optimizing fluorinated substrate structures to achieve high polarization efficiency. This strategy enables efficient prediction of high-enhancement substrates with a minimal number of experiments. A chemical space comprising more than 180 000 fluorinated N-heterocyclic compounds was first constructed, from which fluoropyridine derivatives were selected as the target substrate class for experimental investigation. Principal component analysis (PCA) was used to reduce the dimensionality of the molecular descriptors. Structurally representative substrates that were uniformly distributed within this chemical space were then selected for ^19^F SABRE experiments. To correlate molecular structure with experimentally measured SEFs, random forest (RF) classification was first used to identify the key descriptor governing high- and low-enhancement discrimination, and forward stepwise linear regression was then applied to construct a five-descriptor model for quantitative ranking within the high-enhancement regime. The predictive capability of this workflow was evaluated using six fluorinated pyridine substrates that have not previously been reported in ^19^F SABRE hyperpolarization, and the predicted SEFs of the high-enhancement substrates agreed well with the experimental results. Finally, we achieved simultaneous hyperpolarization of 15 fluorinated pyridine substrates with both high SEFs and well-separated chemical shifts. This workflow overcomes limitations of conventional SABRE hyperpolarization studies by enabling rapid and efficient screening of large numbers of candidate molecules within a virtual chemical space. It provides a rational framework for the design and discovery of high-performance SABRE substrates while substantially reducing experimental screening effort.

## Materials and methods

### Sample preparation

The Ir-based catalyst [IrCl(COD)(IMes)] (COD = *cis*,*cis*-1,5-cyclooctadiene; IMes = 1,3-bis(2,4,6-trimethylphenyl)imidazol-2-ylidene), deuterated methanol, and all fluorinated pyridine compounds used in this study were purchased from commercial suppliers, including Bide Pharmatech, Energy Chemical, Meryer Chemical Technology, and Acmec Biochemical. All chemicals were used as received without further purification. Hyperpolarization samples were prepared inside a glovebox. Fluorinated pyridine substrates and the Ir-based catalyst were codissolved in deuterated methanol (700 µL) and thoroughly mixed. The resulting solution was then transferred to a customized 5 mm NMR tube. The front end of the tube was connected to a polytetrafluoroethylene (PTFE) tube approximately 4 cm in length and 6 mm in inner diameter to facilitate gas exchange. A Y-shaped three-way valve was installed at the front of the PTFE tube to allow the introduction of parahydrogen and the removal of exhaust gas, with one-way valves mounted at both the inlet and outlet to ensure unidirectional gas flow. For single-substrate SABRE experiments, the concentration of fluorinated pyridine was 50 mM, and that of the iridium-based catalyst was 5 mM. For multiplexed experiments, the fluorinated pyridine concentration was reduced to 10 mM, whereas the catalyst concentration was maintained at 5 mM. Parahydrogen with a purity of 96% was generated by passing hydrogen gas over an FeO(OH) catalyst at 35 K. Thermal equilibrium ^19^F NMR spectra were acquired on a 1.4 T benchtop NMR spectrometer (Oxford Instruments, United Kingdom) with 64 scans. The ^19^F *T*_1_ relaxation and one-dimensional selective exchange spectroscopy (1D EXSY) measurements were performed at 298 K. The 1D EXSY experiments were conducted according to previously reported procedures. Briefly, the resonance of the substrate bound to the iridium catalyst was selectively excited, and the transfer of magnetization to the corresponding free-substrate resonance was monitored over a series of mixing times. The integrated intensities of the selectively excited bound-state signal and the observed free-state signal were fitted as a function of mixing time using a single-exponential exchange-buildup model.^35–37^

### SABRE hyperpolarization procedure

Prior to acquisition of the SABRE-hyperpolarized ^19^F NMR spectra, 3 bar of parahydrogen was introduced into the homemade 5 mm NMR tube through the Y-shaped valve. The tube was then shaken in the Earth's magnetic field for 30 s to activate the catalyst *in situ*. Subsequently, the residual parahydrogen inside the tube was vented through the outlet, and fresh parahydrogen at 3 bar was reintroduced within 20 s. The NMR tube was then vigorously shaken in the Earth's magnetic field for 10 s to ensure efficient mixing and interaction between parahydrogen and the solution. Immediately afterward, the tube was rapidly transferred to the 1.4 T benchtop NMR spectrometer for acquisition of the hyperpolarized ^19^F NMR spectrum (Fig. 1b). The calculation of the SEFs and the corresponding polarization levels is described in detail in SI, Part 2.

**Fig. 1:**
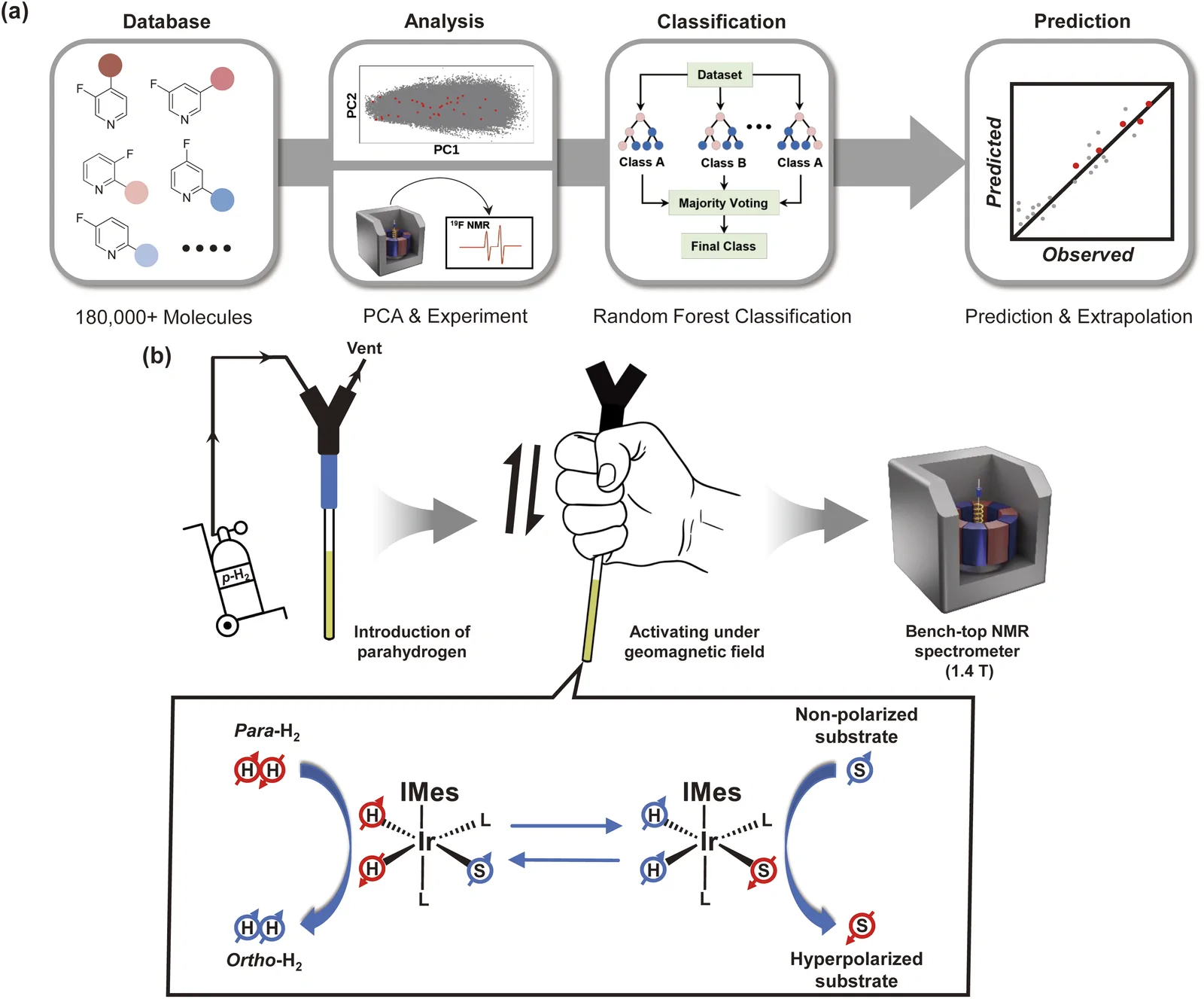
(a) Schematic illustration of the integrated data-science and machine-learning workflow. (b) Experimental procedure for ^19^F SABRE hyperpolarization.

### Data science and machine learning methods

The overall machine learning workflow is illustrated in Fig. 1a. Approximately 180 000 fluorinated N-heterocyclic compounds were downloaded from the PubChem open-access database.^38^ Twenty-eight chemically meaningful two-dimensional molecular descriptors were retained after descriptor filtering and were subsequently used for PCA,^39^ RF classification, and forward stepwise linear regression. The RF classification model was used to analyze the molecular descriptor dataset, and model performance was evaluated by leave-one-out cross-validation. In each iteration, the model was trained on all samples except one and evaluated on the held-out sample. Performance metrics, including accuracy, confusion matrix and the area under the receiver operating characteristic curve, were then used to assess the overall stability and generalization performance of the model.

For quantitative prediction of SEFs, a forward stepwise multivariate linear regression model, as proposed by the Sigman group^40^ was employed. Model parameters were fitted using the ordinary least squares (OLS) method, and LOOCV was applied for model validation. Both the training set and external test set were evaluated using MAE and *R*^2^. DFT calculations were performed for all molecules involved in both classification and regression analyses, yielding a total of 51 quantitative features (Table S1).^41,42^ These features include global molecular descriptors as well as local electronic and geometric descriptors associated with the nitrogen atom of the pyridine ring.

## Results and discussion

### Initial dataset and feature engineering

The workflow proposed in this study is illustrated in Fig. 1a. To comprehensively cover the structural diversity of pyridine derivatives, avoid structural bias, and ensure model generalizability, we first collected more than 180 000 fluorinated N-heterocyclic compounds with molecular weights below 300 from the PubChem database.^38^ To obtain an initial assessment of the chemical diversity within this large compound library, molecular descriptors were calculated using RDKit, yielding 209 descriptors.^43^ Descriptor filtering procedures were then applied to remove non-informative, redundant, and highly collinear features, thereby generating a compact and chemically interpretable descriptor set. Specifically, variance-threshold filtering, removal of single-dominant feature, and high-correlation screening were used to refine the original descriptor set, resulting in 28 discriminative descriptors with clear chemical relevance (Fig. 2a). These descriptors encompass several key molecular properties of fluorinated N-heterocyclic compounds, including electronic properties (MaxAbsEStateIndex, MinEStateIndex, BCUT2D_MWHI, BCUT2D_MRLOW, PEOE_VSA1, PEOE_VSA7, PEOE_VSA8 and PEOE_VSA9), topological features (BalabanJ, BertzCT, SPS, AvgIpc, Ipc, and FpDensityMorgan3), physicochemical properties (MolWt, MolLogP, and TPSA), surface-related properties (EState_VSA2, EState_VSA4, EState_VSA5, VSA_EState2, VSA_EState4, VSA_EState5, VSA_EState7, SMR_VSA5, SMR_VSA7, and SlogP_VSA2), and a shape descriptor (HallKierAlpha).

**Fig. 2:**
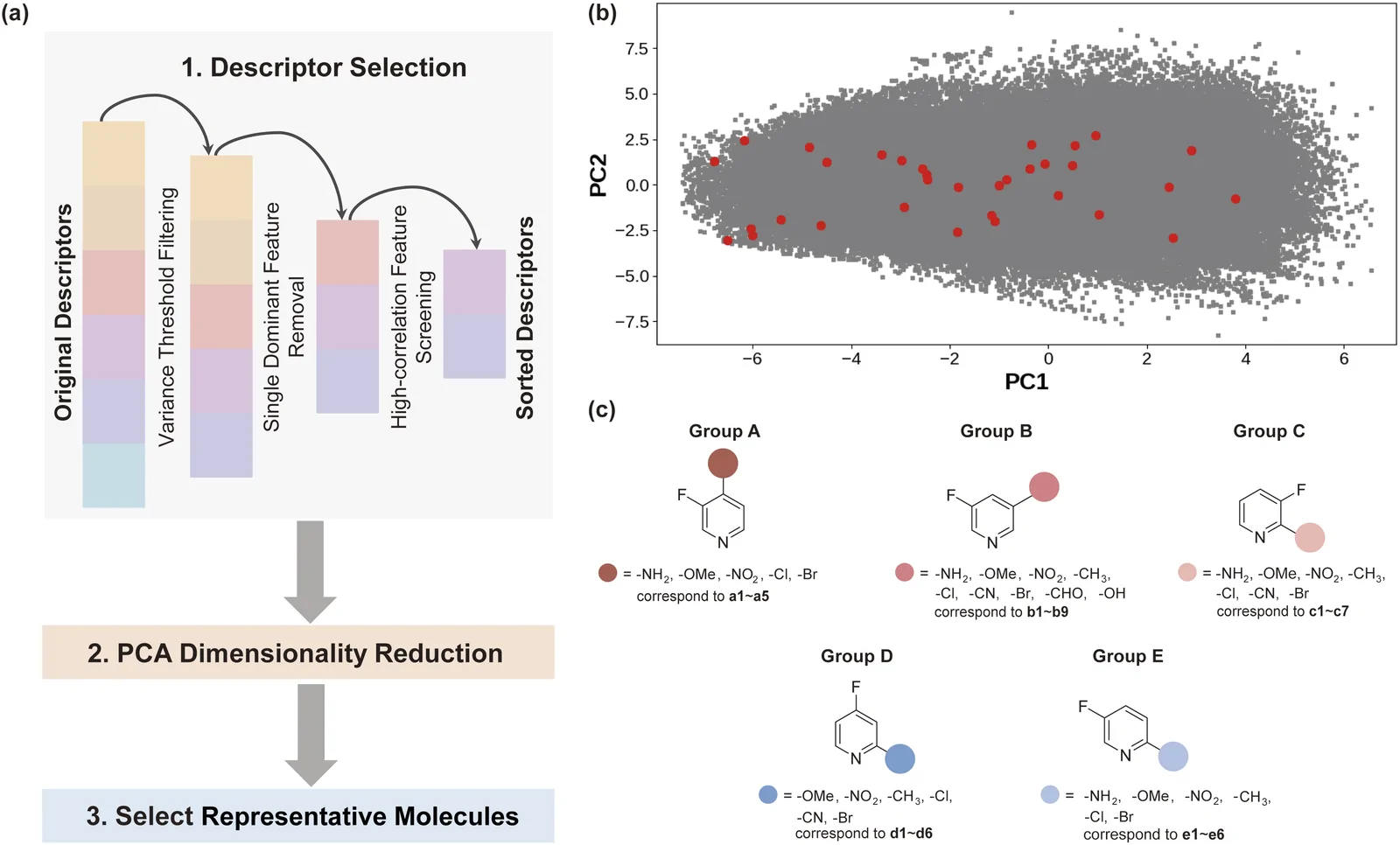
(a) Workflow of PCA-based dimensionality reduction. (b) Two-dimensional PCA projection of the large chemical space containing 180 000 fluorinated N-heterocyclic compounds. Red points indicate the distribution of the 33 structurally distinct fluorinated pyridine substrates from groups A–E within this chemical space. (c) Molecular structures of the fluorinated pyridine derivatives in groups A–E, which constitute the training set.

### Chemical space PCA and substrate selection

To visualize the distribution of candidate molecules in chemical space, the standardized descriptor matrix was projected onto the first two principal components (PC1 and PC2). Guided by this PCA representation, 33 representative fluoropyridine substrates were manually selected by considering the positions of fluorine and other substituents on the pyridine ring, their electronic and structural characteristics, molecular diversity, coverage of the PCA space, commercial availability, and experimental feasibility (Fig. 2b). Given the substantial multicollinearity among the 28 descriptors, PCA is particularly suitable because it transforms the original correlated variables into a set of mutually orthogonal components through linear transformation, thereby reducing information redundancy and helping to prevent model overfitting. Compared with nonlinear approaches such as *t*-SNE, which primarily emphasize local clustering patterns, PCA better preserves the global topological structure of the chemical space, thereby ensuring that the 33 selected molecules provide strong statistical representativeness of the overall feature space. Based on the relative positions of the fluorine atom and the substituent on the pyridine ring, the 33 fluorinated pyridine substrates were classified into five groups: group A, in which the fluorine atom and substituent are located at the 3- and 4-positions of the pyridine ring, respectively; group B, with substitution at the 3- and 5-positions; group C, with substitution at the 3- and 2-positions; group D, with substitution at the 4- and 2-positions; and group E, with substitution at the 5- and 2-positions (Fig. 2c and S1–S33).

### ^19^F SABRE experiments

Each of the five groups of fluorinated pyridine derivatives was independently evaluated in ^19^F SABRE hyperpolarization experiments without the addition of any co-ligands. In a typical experiment, a single substrate (50 mM) was mixed with [IrCl(COD)(IMes)] (5 mM) in 700 µL of deuterated methanol. The mixture was transferred to a sealed NMR tube. The tube was then shaken in the Earth's magnetic field for 30 s to activate the catalyst *in situ*. Subsequently, the residual parahydrogen was vented, fresh parahydrogen (3 bar) was reintroduced within 20 s, and the sample was vigorously shaken for 10 s before rapid transfer to the 1.4 T benchtop NMR spectrometer. Analysis of the SEFs of the fluorinated pyridine substrates (Fig. 3a and S40–S72) revealed pronounced differences in hyperpolarization performance. Certain substrates exhibited exceptionally high SEFs; for example, 3-fluoro-4-methoxypyridine showed a ^19^F enhancement of 6979-fold (Fig. S41). In contrast, other substrates displayed negligible enhancement or no detectable hyperpolarized signal, such as 2-methoxy-3-fluoropyridine and 2-bromo-3-fluoropyridine (Fig. S55 and S60).

**Fig. 3:**
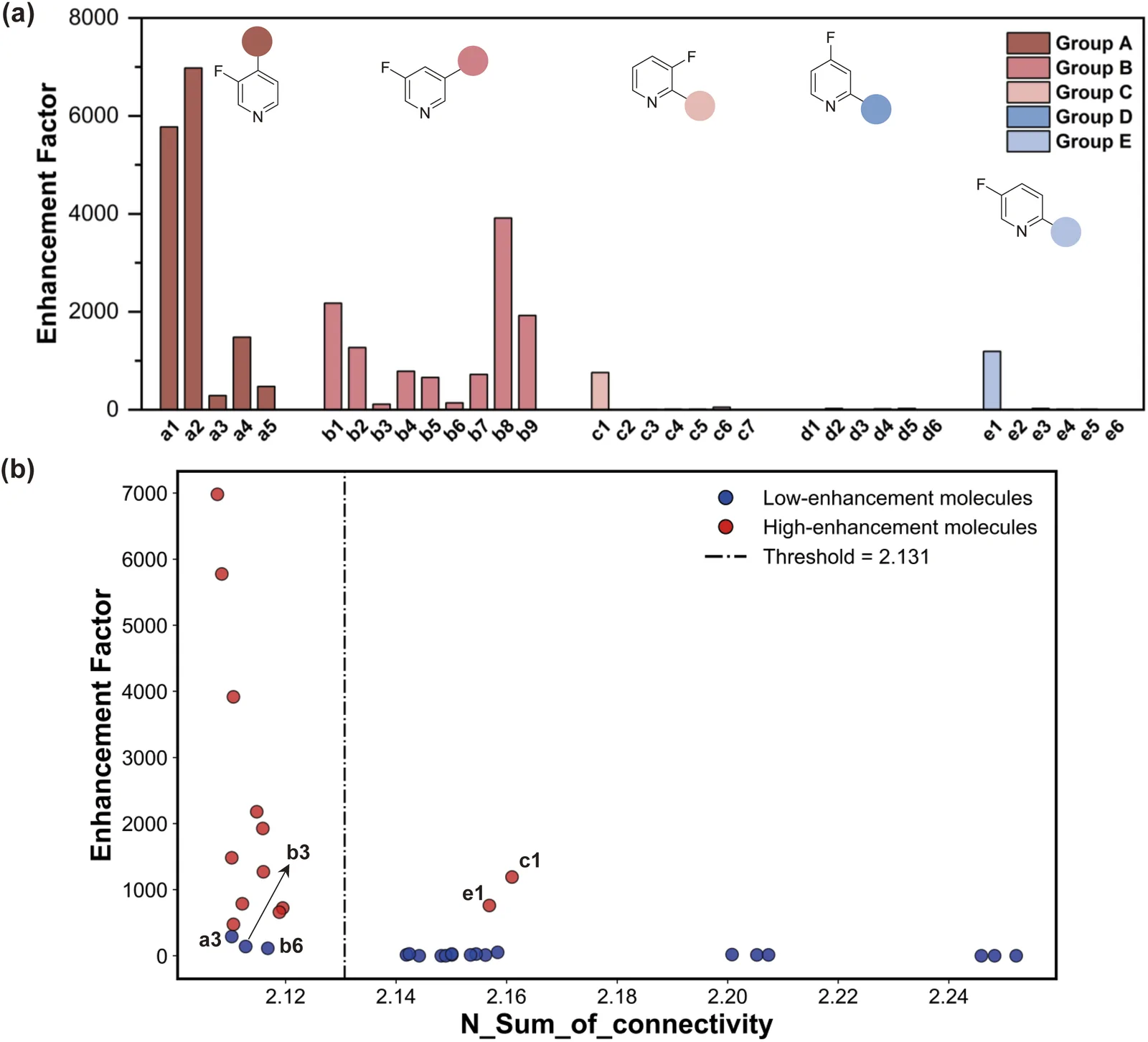
(a) SEFs of ^19^F SABRE hyperpolarization for the 33 fluorinated pyridine substrates in groups A–E. (b) Classification scatter plot based on the most important descriptor, N_Sum_of_connectivity. The threshold value is 2.132; blue points represent low-enhancement substrates (SEF < 300), whereas red points represent high-enhancement substrates (SEF > 300).

### QSAR-based feature extraction

The next objective was to identify the molecular parameters that play decisive roles in hyperpolarization efficiency. To construct a quantitative structure–activity relationship (QSAR) model with strong mechanistic interpretability, density functional theory (DFT) calculations were further performed for the 33 fluorinated pyridine substrates, optimized at the B3LYP-D3(BJ)/def2-TZVP level, in combination with NBO and Multiwfn^44,45^ analyses to generate 51 molecular descriptors. These descriptors capture global electronic properties (frontier orbital energies and multipole moments), structural and conformational characteristics (geometric and topological descriptors, planarity, and conformational parameters), surface and distribution properties (molecular volume, density, surface area, and electrostatic potential surface distributions), as well as local and reactivity-related descriptors (local reactivity indices, energy descriptors, and pyridine-nitrogen-related descriptors), as summarized in Table S1 in the SI. After combining these variables with the 28 descriptors from RDKit and removing highly correlated variables, a total of 77 descriptors were ultimately retained for model construction.

### Classifier construction and validation

To enable accurate prediction and classification of the enhancement performance of fluoropyridine molecules, a nonlinear RF classifier was established. To determine the optimal classification boundary, the SEF threshold was varied from 200 to 600, and the performance of the RF classifier was evaluated using precision, recall, and *F*_1_-score under LOOCV (Table S2). Because the model showed similarly strong performance within the 300–400 interval, an SEF threshold of 300 was selected to retain higher sensitivity while preserving robust classification accuracy.

Using an SEF threshold of 300, the samples were categorized into high- and low-performance classes, and the complex relationships between molecular descriptors and enhancement behavior were learned using an ensemble of 500 decision trees. Model performance was systematically evaluated by leave-one-out cross-validation (LOOCV), which yielded an accuracy of 0.88. Further evaluation using the confusion matrix and receiver operating characteristic (ROC) analysis showed that the model achieved a recall of 1.00 for low-enhancement molecules and a precision of 1.00 for high-enhancement molecules, indicating zero false negatives in the former and zero false positives in the latter (Fig. S73 and Table S2). The AUC value of 0.92 further confirmed the robustness and discriminative capability of the model for complex molecular screening (Fig. S75). To identify the molecular descriptors most relevant to ^19^F SABRE enhancement efficiency, feature importance from the RF model was quantitatively analyzed and ranked. Feature importance was defined according to each descriptor's contribution to the reduction in Gini impurity across all 500 trees. To improve interpretability, the splitting thresholds of each feature were extracted from the individual trees in the ensemble. The decision boundaries of the key descriptors were then determined from the median splitting thresholds of the most frequently selected features. Among all descriptors, the total local topological connectivity of the pyridine nitrogen atom (N_Sum_of_connectivity) was identified as the most influential feature, ranking first in the importance analysis (Fig. S77). Subsequent threshold analysis (Fig. 3b) revealed that when N_Sum_of_connectivity exceeds 2.132, the substrates are more likely to be classified into the low-enhancement category. In Fig. 3b, red points represent high-enhancement substrates (SEF > 300), whereas blue points correspond to low-enhancement substrates (SEF < 300). To further validate the robustness of the classification results and to disentangle the linear components of feature contributions, an L1-regularized logistic regression (LR) model was introduced for comparison. Evaluation of the LR classifier showed an accuracy of 0.90 under LOOCV, with an AUC of 0.95 (Fig. S74 and S76), demonstrating predictive performance highly consistent with that of the RF model. Notably, the descriptor with the largest absolute coefficient identified by the LR model was fully consistent with the top-ranked feature importance obtained from the RF model, namely N_Sum_of_connectivity (Fig. S78). This cross-algorithm consistency further supports the importance of N_Sum_of_connectivity as a key descriptor governing molecular enhancement performance.

From a chemical perspective, an increased N_Sum_of_connectivity value reflects the presence of substituents adjacent to the pyridine nitrogen atom, which can hinder effective coordination between the nitrogen donor and the iridium catalyst center. This steric obstruction reduces the efficiency of substrate binding and polarization transfer, thereby lowering hyperpolarization efficiency. This interpretation is consistent with the established empirical understanding that increased steric bulk adversely affects ligand coordination to iridium centers.^28^ In this context, N_Sum_of_connectivity is best interpreted as a data-derived measure of the local connectivity and steric accessibility around the pyridine nitrogen atom, which may indirectly reflect steric constraints on substrate coordination and exchange at the iridium center. Rather than representing a new physical principle, this descriptor provides a quantitative representation of established coordination-chemical and steric effects within molecular descriptor space.

It should be emphasized, however, that the efficiency of SABRE polarization transfer and the resulting signal enhancement are not determined solely by molecular structure. Instead, they arise from the combined effects of multiple physical and chemical factors, including, but not limited to, substrate–iridium coordination, chemical-exchange kinetics, polarization-transfer conditions, and nuclear-spin relaxation. For example, the mechanistic studies of Barskiy and co-workers demonstrated that reversible substrate association and dissociation, formation of the coordination complex, and the associated exchange kinetics are important determinants of SABRE polarization efficiency.^46,47^ Rayner, Duckett, and co-workers further demonstrated that the steric and electronic properties of the catalyst–substrate system can regulate substrate coordination, ligand-exchange kinetics, relaxation behavior, and ultimately SABRE efficiency.^48^

### Linear regression and external testing

Based on the RF classification results, 13 fluorinated pyridine derivatives with relatively high SEFs, defined as greater than 300-fold, were selected for subsequent regression analysis. The quantitative regression step was therefore explicitly restricted to the high-enhancement regime identified by the first-stage classifier. In the proposed two-stage workflow, the RF classifier serves as an initial screening step, whereas the regression model is used only to rank and prioritize candidates classified as high-enhancement. It is not intended to provide quantitative predictions for low-enhancement or poorly polarizable substrates. To evaluate different algorithms for quantitative prediction within this regime, three models were compared: partial least squares regression (PLS), RF regression, and forward stepwise linear regression. For the PLS model, the optimal number of latent variables was selected based on the LOOCV *Q*^2^ value. RF regression was implemented as an ensemble of 100 decision trees with a maximum depth of 5 and was likewise evaluated by LOOCV. Forward stepwise regression started from an empty model and introduced new descriptors only when their inclusion improved the LOOCV *Q*^2^ value, thereby reducing the risk of overfitting in this small-sample setting.

Comparative analysis of the three models (Fig. S79–S81) revealed substantial differences in predictive performance. Forward stepwise linear regression delivered the best performance, with *R*^2^ = 0.92 and *Q*^2^ = 0.74 (Fig. S79). By contrast, the RF model showed relatively strong fitting ability (*R*^2^ = 0.88) but substantially poorer predictive capability (*Q*^2^ = 0.29; Fig. S80), indicating an elevated risk of overfitting. The PLS model performed worst, with *R*^2^ = 0.57 and *Q*^2^ = −0.51 (Fig. S81), suggesting that its predictive ability was poorer than that of a simple mean-response model. These results indicate that, within the high-enhancement regime considered here, forward stepwise linear regression offers the most robust balance between interpretability and predictive performance.

Ultimately, five key descriptors were selected to construct an interpretable multivariate linear regression model (Fig. 4a): Neg_Average (average negative molecular surface electrostatic potential), HOMO_LUMO_Gap (HOMO–LUMO energy gap), Nu (charge imbalance), ESPmax (maximum electrostatic potential), and Quadrupole_Moment (molecular quadrupole moment). The resulting model achieved *R*^2^ = 0.92 and a mean absolute error (MAE) of 421, indicating strong predictive performance within the high-SEF subset studied here (Fig. 4a). To rule out chance correlation and further assess model robustness, 500 Y-randomization tests were performed on the forward stepwise linear regression model (Fig. S82). In each trial, the response values (SEFs) were randomly permuted while the descriptor matrix was kept unchanged. The randomized models yielded a mean *Q*^2^ value of −1.38, which was substantially lower than that of the original model (*Q*^2^ = 0.74), confirming that the predictive performance of the model did not arise from random statistical correlation.

**Fig. 4:**
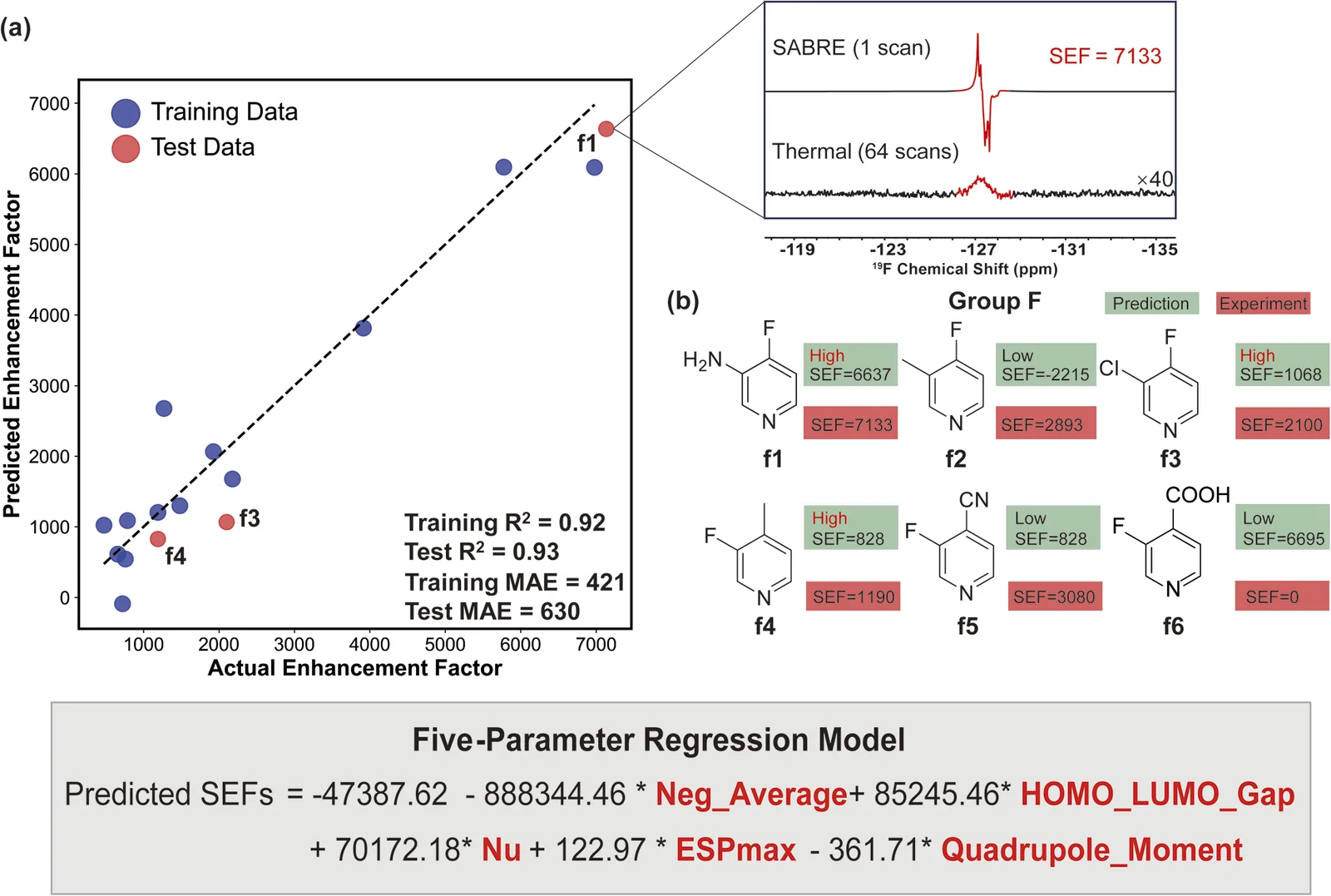
(a) Interpretable five-variable linear regression model for quantitative ranking of fluorinated pyridine substrates within the high-enhancement regime identified by the first-stage classifier. The training set (blue circles; SEF > 300) yields *R*^2^ = 0.92 with MAE = 421, while the external high-SEF test substrates (red circles) show *R*^2^ = 0.93 with MAE = 630. (b) Molecular structures of the external validation substrates (group F).

The multicollinearity of the five core descriptors was further examined using the variance inflation factor (VIF). All descriptors showed VIF values below 3, well below the conventional threshold of 10 for severe multicollinearity, and all corresponding *P* values were below 0.05 (Table S4). These results indicate that the selected descriptors are statistically significant and largely independent, supporting the stability and reliability of the regression model. Notably, all five selected variables are global molecular descriptors rather than local structural parameters, suggesting that ^19^F SABRE hyperpolarization efficiency is governed primarily by the overall electronic structure and electrostatic properties of fluorinated pyridine substrates.

Within the model, Neg_Average shows a significant negative correlation with the enhancement factor, indicating that a lower average negative surface electrostatic potential, that is, stronger nucleophilicity, favors coordination between the substrate and the iridium catalyst center. In contrast, both HOMO_LUMO_Gap and Nu exhibit positive correlations with the ^19^F enhancement factor. A larger HOMO_LUMO_Gap reflects higher kinetic stability and lower chemical reactivity, which may facilitate stabilization of key catalytic intermediates as σ-donor substrates and prolong longitudinal relaxation times (*T*_1_), thereby improving polarization transfer efficiency.^36^ A higher Nu value indicates greater charge imbalance, implying enhanced electron-donating ability of electron-rich regions—particularly the pyridine nitrogen atom—thus promoting substrate coordination to the catalyst. Quadrupole_Moment is negatively correlated with the enhancement factor, suggesting that molecules with more uniform charge distributions (smaller quadrupole moments) form more symmetric and stable coordination environments with the iridium catalyst. Conversely, ESPmax exhibits a positive correlation with the enhancement factor. We found that higher ESPmax values (ESPmax > 40) are associated with pyridine substituents capable of coordinating to the iridium catalyst, such as –OH and –NH_2_ groups (Fig. S83). These high-ESPmax sites promote localization of electron density at coordination atoms, thereby facilitating metal–substrate bond formation and efficient polarization-transfer pathways.

To assess whether the molecular descriptors provide predictive information beyond established chemical knowledge, we constructed a chemically informed baseline model using the measured ^19^F *T*_1_ values and substrate dissociation rates (*k*_d_). For the high-enhancement fluoropyridine subset, the baseline model yielded *R*^2^ = 0.54 by direct fitting and a leave-one-out cross-validation (LOOCV) *Q*^2^ = 0.14 (Table S5 and Fig. S84). These results indicate that, although relaxation and exchange kinetics are mechanistically relevant, these variables alone are insufficient to explain the variation in enhancement among the substrates. By comparison, the forward stepwise linear regression model showed substantially better fitting and cross-validation performance (*R*^2^ = 0.92 and *Q*^2^ = 0.74). The selected molecular descriptors may collectively capture information on the overall electronic structure, molecular-surface electrostatic potential, and charge distribution of the substrates. These properties may jointly influence substrate coordination, complex stability, and the polarization-transfer environment, thereby providing complementary predictive information beyond *T*_1_ and substrate dissociation rate alone.

After construction of the linear regression model, its predictive capability was further evaluated through external validation. A set of candidate molecules was selected as the external validation set, including 3-amino-4-fluoropyridine (f1), 3-methyl-4-fluoropyridine (f2), 3-chloro-4-fluoropyridine (f3), 3-fluoro-4-methylpyridine (f4), 3-fluoro-4-cyanopyridine (f5) and 3-fluoropyridine-4-carboxylic acid (f6) (Fig. 4b and S34–S39). The classification model divided these six substrates into high- and low-enhancement groups, after which their ^19^F SABRE enhancement factors were predicted using the linear regression model and compared with the experimental results (Fig. S85–S90). For the low-enhancement substrates, the predicted SEF values deviated markedly from the experimental values (Table S6). By contrast, the predicted SEF values for the high-enhancement substrates were in much better agreement with experiment, likely because the linear regression model was trained exclusively on substrates with SEFs greater than 300. Overall, the predicted and experimental SEFs showed good agreement for the high-SEF substrates in the external test set (Fig. 4a), yielding *R*^2^ = 0.93 and MAE = 630. These results demonstrate the practical utility of the model for prioritizing highly efficient hyperpolarizable substrates within the high-enhancement regime. Notably, 3-amino-4-fluoropyridine (f1) exhibited outstanding hyperpolarization performance, with an experimentally measured enhancement factor of 7133, corresponding to a ^19^F polarization of 3.2%. This value exceeded the highest enhancement observed in the training set. These results further support the practical utility of the two-stage workflow for prioritizing and quantitatively ranking high-enhancement candidates.

### Multiplexed ^19^F SABRE hyperpolarization

To demonstrate the feasibility of multiplexed ^19^F detection, 15 fluorinated pyridine substrates were manually selected from the training and external test sets based on their measured SEFs and chemical-shift separation. The substrates (10 mM each) and iridium catalyst (5 mM) were dissolved in deuterated methanol (700 µL). After catalyst activation, the sample was polarized in the Earth's magnetic field and transferred to a 1.4 T benchtop NMR spectrometer for single-scan ^19^F NMR acquisition (Fig. 5). All 15 hyperpolarized signals were clearly resolved and assigned over a chemical-shift range of −106 to −153 ppm. This experiment therefore serves as a downstream application demonstration rather than an independent validation of the machine-learning model.

**Fig. 5:**
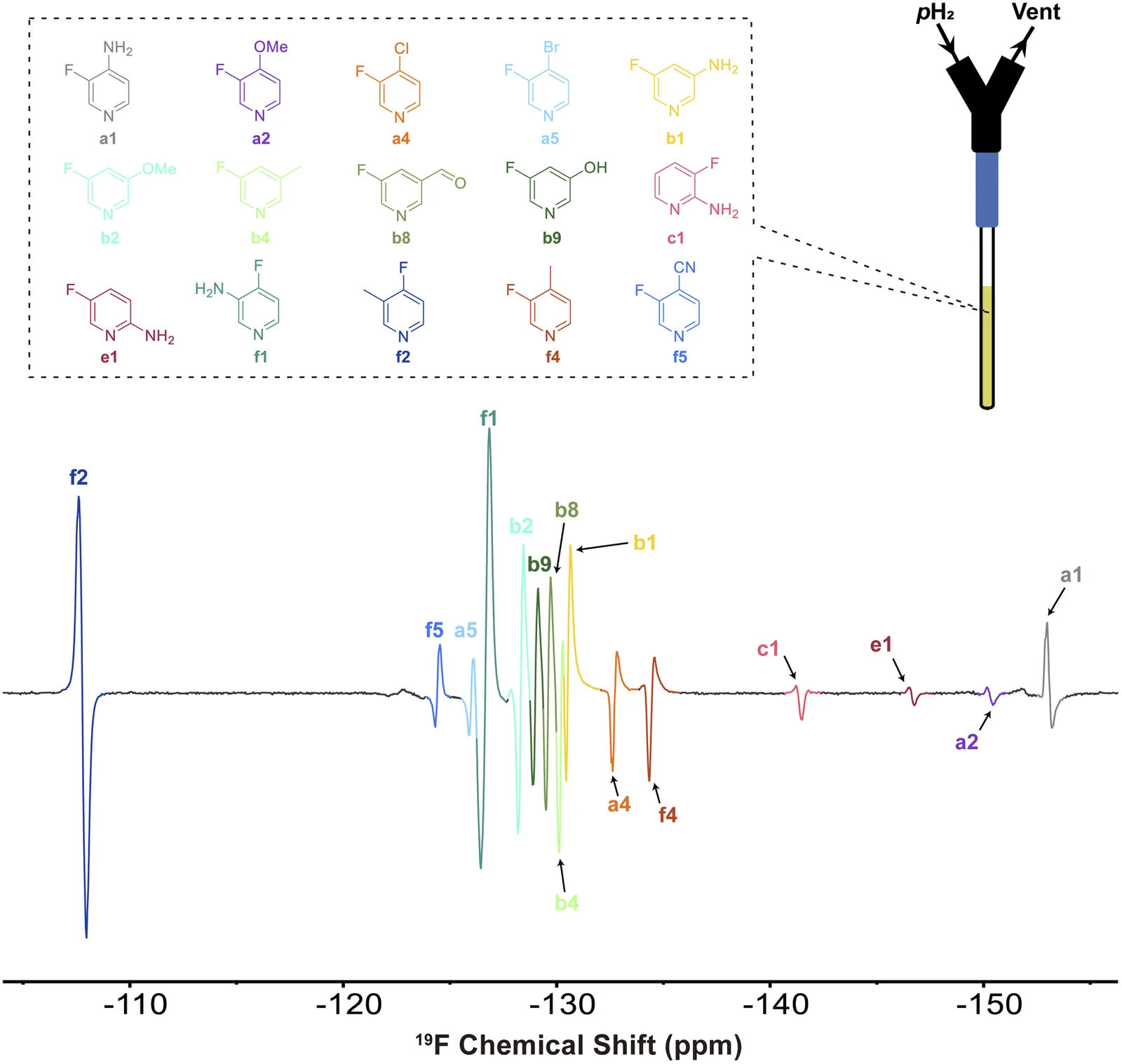
Multiplexed ^19^F SABRE hyperpolarized spectrum of a mixture of fifteen fluorinated pyridine substrates (10 mM each).

## Conclusions

In this work, we integrated data science, machine learning, and SABRE hyperpolarization experiments to systematically elucidate the molecular factors governing ^19^F SABRE hyperpolarization efficiency. By constructing a chemical space comprising more than 180 000 fluorinated N-heterocyclic compounds and combining principal component analysis with experimental sampling, we established a representative training set that enabled efficient exploration of structure–performance relationships with minimal experimental effort. The resulting workflow operates in two stages: a classification model first identifies fluorinated pyridine substrates with a high likelihood of strong SABRE enhancement, and a second-stage interpretable linear regression model then provides quantitative ranking within this high-enhancement regime. Through RF classification, we identified descriptors that discriminate between high- and low-enhancement substrates, and by focusing the regression analysis on substrates with SEFs greater than 300 we obtained a five-descriptor quantitative structure–activity relationship for high-SEF candidates. Accordingly, this regression model should not be regarded as a general predictor of SABRE performance across all substrate classes. This model successfully extrapolated to 3-amino-4-fluoropyridine, which exhibited a ^19^F signal enhancement of 7133-fold, corresponding to a polarization level of approximately 3.2% at 1.4 T without co-ligands, in strong agreement with the model prediction. In conclusion, this study establishes a machine-learning-assisted framework for screening and ranking fluorinated pyridine substrates within the investigated chemical space.

Beyond single-substrate optimization, the proposed approach was further applied to multiplexed SABRE hyperpolarization, enabling single-scan detection of 15 fluorinated pyridine substrates on a benchtop NMR spectrometer. Collectively, this study establishes an interpretable and experimentally efficient strategy for SABRE substrate discovery within fluorinated pyridine-type substrates under the present experimental conditions. More broadly, the workflow may provide a basis for future data-driven optimization of hyperpolarization processes and could be extended to other nuclei after further validation. However, expanding the scope of SABRE cannot rely solely on structural screening of N-heterocycles that already exhibit favorable coordination behavior. Previous studies have shown that co-ligands such as DMSO, DPSO, acetonitrile, and allylamine can improve the SABRE polarization of weakly coordinating substrates by modifying the catalyst coordination environment and exchange kinetics.^49–51^ Future data-driven screening should therefore be extended beyond substrate descriptors to include catalyst structure, co-ligand identity, and reaction conditions. Such an integrated strategy may broaden the present framework from ranking known polarizable substrates to identifying suitable conditions for poorly polarizable substrates and more diverse molecular classes. On the computational side, the present study is limited by the statistical constraints of a high-dimensional, small-sample dataset, and future efforts should therefore expand both the size and diversity of the dataset. A larger dataset would also provide a basis for applying deep-learning approaches, such as artificial neural networks, to capture more complex nonlinear relationships between molecular descriptors and hyperpolarization performance. In addition, integrating active-learning-based sample selection may enable more accurate prediction and rational design of high-performance fluoropyridines across a broader chemical space. A practical limitation of large-scale, data-driven SABRE substrate optimization is that most candidate molecules are not commercially available; consequently, even substrates identified computationally as highly promising may be difficult to procure or synthesize. Furthermore, the current study represents substrate molecules using one-dimensional SMILES strings. Future work could explore two-dimensional or three-dimensional molecular representations, potentially in combination with Bayesian optimization, to further improve predictive accuracy and accelerate molecular discovery.

## Author contributions

Qiwei Peng, Li Zheng, Xiaohong Cui, Zhong Chen and Xinchang Wang designed research; Qiwei Peng, Li Zheng, Huijun Sun, Yubin Xiong and Zeyu Zheng performed research; Qiwei Peng analyzed data; Qiwei Peng and Xinchang Wang wrote the paper; and all authors reviewed and approved the manuscript.

## Conflicts of interest

The authors declare no competing interest.

## Supplementary Material

SC-017-D6SC04732G-s001

## Data Availability

All relevant code is available on GitHub at https://github.com/Wang-Group-Parahydrogen/19F-SABRE-Substrates-Filter. Additional data supporting the findings of this study are available in the article and its supplementary information (SI). Supplementary information: experimental details, molecular descriptors, model-validation results, and additional NMR spectra. See DOI: https://doi.org/10.1039/d6sc04732g.
