# Supplementary material for: Machine learning-assisted molecular design for efficient 19F hyperpolarization
Source: Chem Sci. 2026 Aug 27;17(40):19428–37. doi: 10.1039/d6sc04732g (PMC13531425; doi:10.1039/d6sc04732g)
Supplement: Supplementary file 1 [file SC-017-D6SC04732G-s001.pdf]

Supplementary Information (SI) for Chemical Science.
This journal is © The Royal Society of Chemistry 2026

Electronic Supplementary Information for

Machine learning-assisted molecular design for efficient ^{19}F hyperpolarization

Qiwei Peng[‡], Li Zheng[‡], Huijun Sun, Yubin Xiong, Zeyu Zheng, Xiaohong Cui, Zhong Chen, Xinchang Wang[*]

[*]Corresponding author. Email: xcwang@xmu.edu.cn

[‡]These authors contributed equally to this work.

Table of Contents

1 Experimental Materials S3

2 ^{19}F SABRE Experiments S4

3 Computational Details Based on Gaussian 16 and Multiwfn S6

4 ^{19}F NMR Chemical Shifts of SABRE Substrates (Group A–F) S7

5 Single Substrate SABRE Hyperpolarized ^{19}F NMR Spectra S27

6 Figures and Tables for Machine Learning S44

7 Chemically Informed Parameters Relevant to ^{19}F SABRE Enhancement S51

8 Hyperpolarized NMR Spectra and Predicted Results for Group F (Test Set) S53

1 Experimental Materials

A total of thirty-nine substrates, including **a1–a5**, **b1–b9**, **c1–c7**, **d1–d6**, **e1–e6**, and **f1–f6**, were purchased from commercial suppliers (Bide Pharmatech, Energy Chemical, Meryer Chemical Technology, and Acmec Biochemical) and used as received without further purification. All ^{19}F SABRE hyperpolarization experiments were performed on a 60 MHz benchtop NMR spectrometer (Oxford Instruments, Tubney Woods, Abingdon, Oxfordshire, OX13 5QX, United Kingdom). The ^{19}F NMR chemical shifts of individual substrates were determined using a Bruker AVANCE NEO 600 MHz nuclear magnetic resonance spectrometer. A series of 1D EXSY experiments was performed at 298 K by selectively exciting the resonance of the substrate bound to the iridium catalyst. The evolution of the free-substrate signal was then monitored as a function of the mixing time to probe substrate dissociation and exchange kinetics.[1–3]

2 ^{19}F SABRE Experiments

Parahydrogen with a purity of 96% was generated by passing hydrogen gas over an FeO(OH) catalyst at 35 K using a liquid-helium-based cryogenic compression refrigerator. The enriched parahydrogen was stored in a high-pressure steel cylinder prior to use. All hyperpolarization samples were prepared inside a glovebox. Custom-made NMR tubes were fabricated by attaching a polytetrafluoroethylene (PTFE) tube (approximately 4 cm in length and 6 mm in inner diameter) to the front end of a 5 mm NORELL NMR tube. A Y-shaped three-way valve was installed at the front of the PTFE tube, and one-way gas valves were mounted at both the inlet and outlet to prevent backflow of parahydrogen.

For single-substrate ^{19}F SABRE experiments, the fluorinated substrate (50 mM) and the Ir-based catalyst [IrCl(COD)(IMes)] (5 mM) were co-dissolved in 700 μL of deuterated methanol. For multiplexed ^{19}F SABRE experiments, each substrate was dissolved at a concentration of 10 mM together with 5 mM of the Ir-based catalyst in 700 μL of deuterated methanol. Prior to data acquisition, 3 bar of parahydrogen was rapidly introduced into the NMR tube within 20 s, followed by vigorous shaking in the Earth's magnetic field for approximately 30 s to fully activate the catalyst. During the experiment, the parahydrogen used for activation was first vented, after which fresh parahydrogen (3 bar) was reintroduced into the NMR tube within 20 s. The sample was then shaken vigorously in the Earth's magnetic field for 10 s and immediately transferred to the benchtop NMR spectrometer for signal acquisition. The transfer time of the NMR tube was approximately 2 s.

The ^{19}F NMR signal enhancement factor and nuclear polarization were calculated as follows:

$$\varepsilon_{SNR} = \frac{SNR(pol)}{SNR(unpol)}\sqrt{\frac{ns(unpol)}{ns(pol)}}$$

where $\varepsilon_{\mathrm{SNR}}$ is the enhancement factor, SNR(pol) and SNR(unpol) denote the signal-to-noise ratios of the hyperpolarized and thermal equilibrium samples, respectively, ns(unpol) and ns(pol) represent the number of scans used for the thermal equilibrium and hyperpolarized spectra. For enhancement calculations, both hyperpolarized and thermal equilibrium spectra were acquired on the same NMR spectrometer using identical pulse sequences, acquisition parameters, excitation angles, and receiver gains. The only difference was the number of scans: 64 scans were used for the thermal equilibrium spectrum, whereas a single scan was used for the hyperpolarized spectrum.

The ^{19}F nuclear polarization (P) was calculated using the following expression:

$$P = \varepsilon \frac{\gamma B_0 \hbar}{2 k_B T}$$

where ε is the enhancement factor, γ is the gyromagnetic ratio, B_0 is the magnetic field strength, $\hbar$ is the reduced Planck constant, k_B is the Boltzmann constant, and T is the absolute temperature. All ^{19}F NMR data were processed using Mestrelab Research Mnova (version 14.0.0). Signal-to-noise ratios were calculated using the built-in SNR calculation function in Mnova software. Prior to SNR calculation, all spectra were processed with a 1 Hz exponential line broadening function to optimize spectral resolution.

3 Computational Details Based on Gaussian 16 and Multiwfn

All calculations were performed using the Gaussian 16 software package (Revision C.01)[4] within the framework of density functional theory (DFT). Geometry optimizations were carried out using an implicit solvent model with the B3LYP[5] hybrid functional, augmented by Grimme's DFT-D3 dispersion correction with Becke–Johnson damping (GD3-BJ).[6] Given the relatively small size of the systems studied, the def2-TZVP[7] basis set was employed for all atoms.

All optimized structures were confirmed to be local minima on the potential energy surface, as evidenced by the absence of imaginary vibrational frequencies. Wavefunction calculations were performed at the B3LYP/def2-TZVP level, and Kohn–Sham wavefunctions were generated in Gaussian 16. Natural bond orbital (NBO) analysis was conducted to obtain electronic structure information. The resulting wavefunction files were subsequently used as input for the Multiwfn[8,9] program to extract global electronic structure descriptors, surface distribution properties, and local reactivity-related descriptors of the molecules.

4 ^{19}F NMR Chemical Shifts of SABRE Substrates (Group A–F)

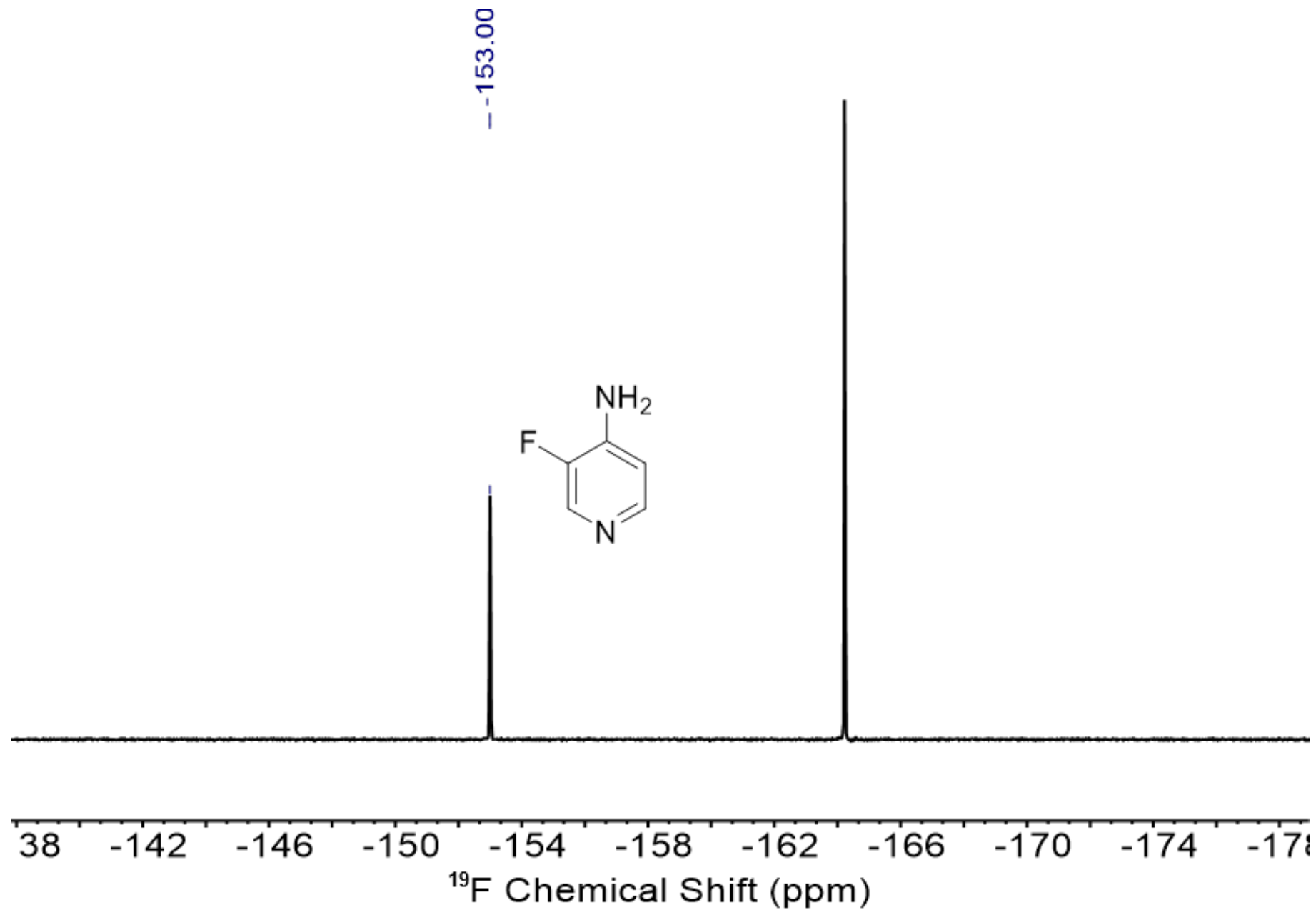

Fig. S1. ^{19}F NMR spectrum of substrate **a1**.

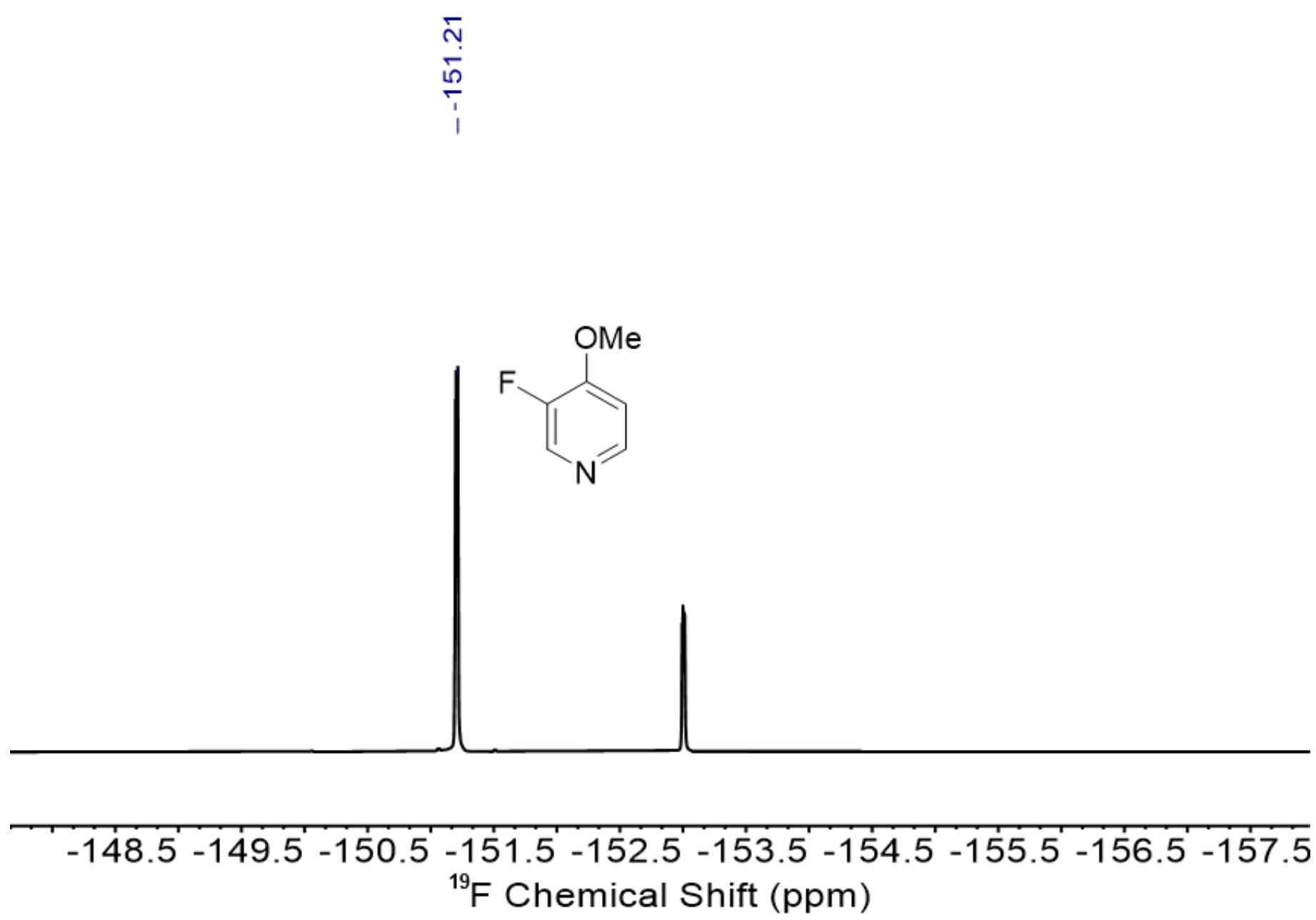

Fig. S2. ^{19}F NMR spectrum of substrate **a2**.

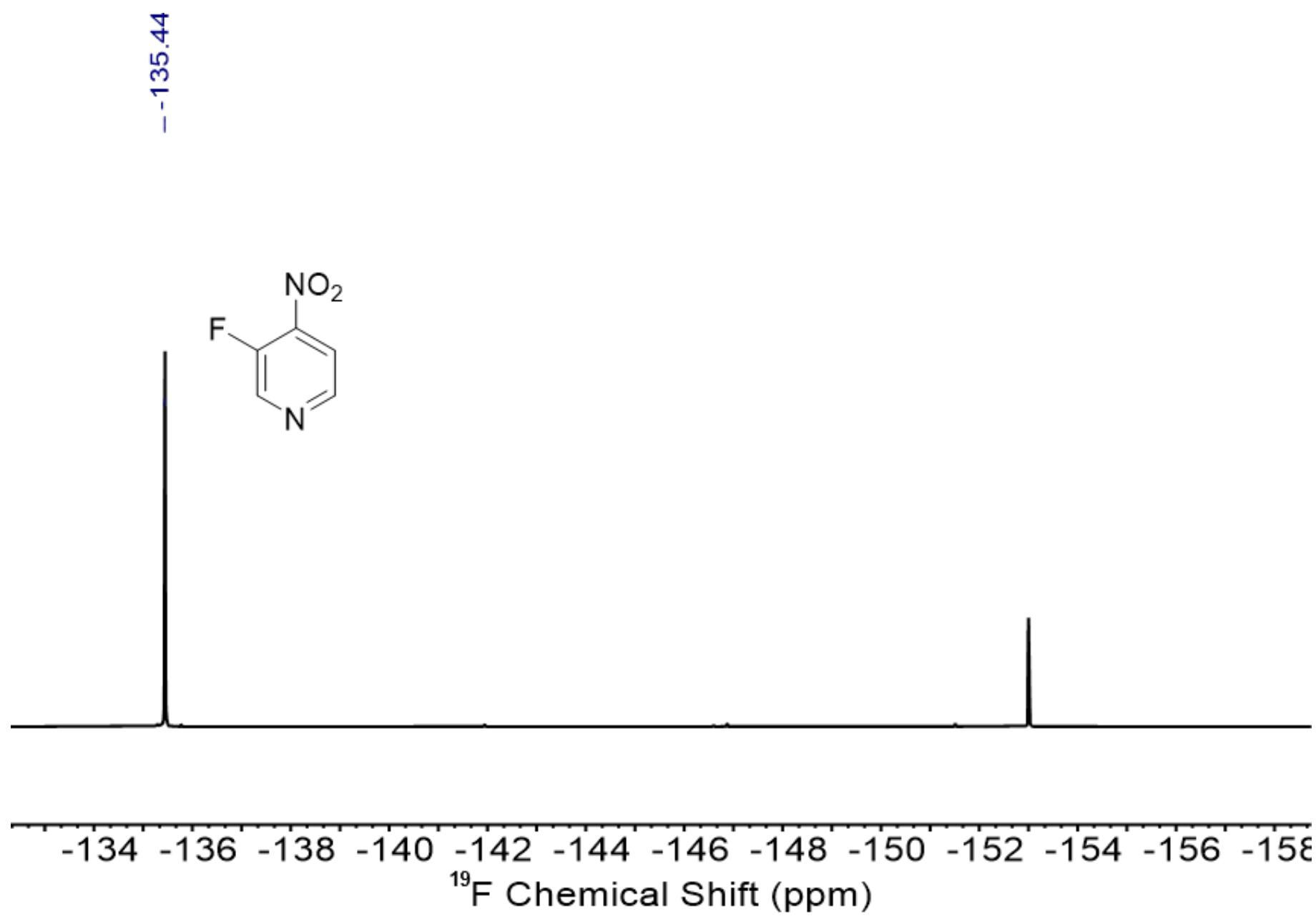

Fig. S3. ^{19}F NMR spectrum of substrate **a3**.

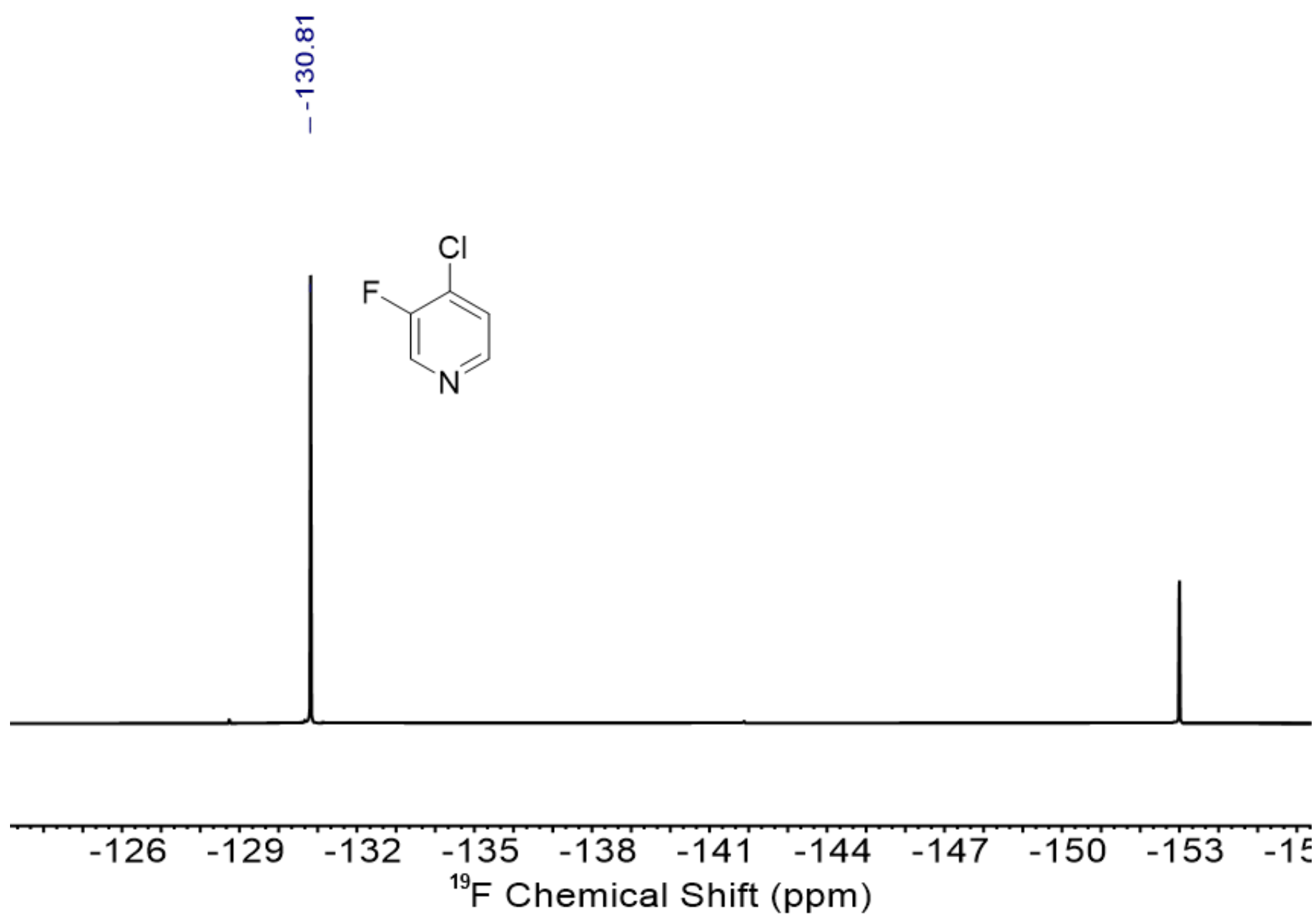

Fig. S4. ^{19}F NMR spectrum of substrate **a4**.

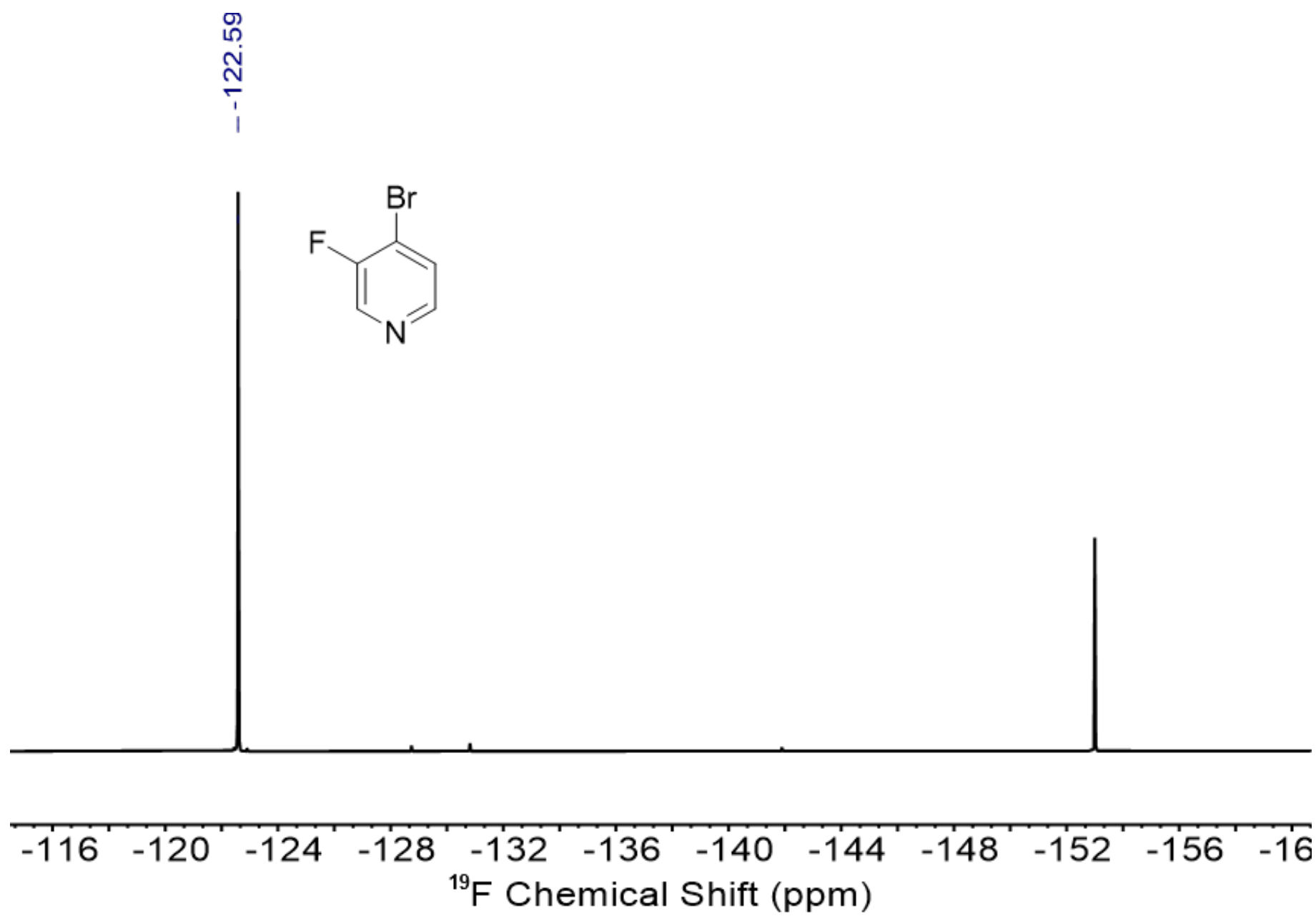

Fig. S5. ^{19}F NMR spectrum of substrate **a5**.

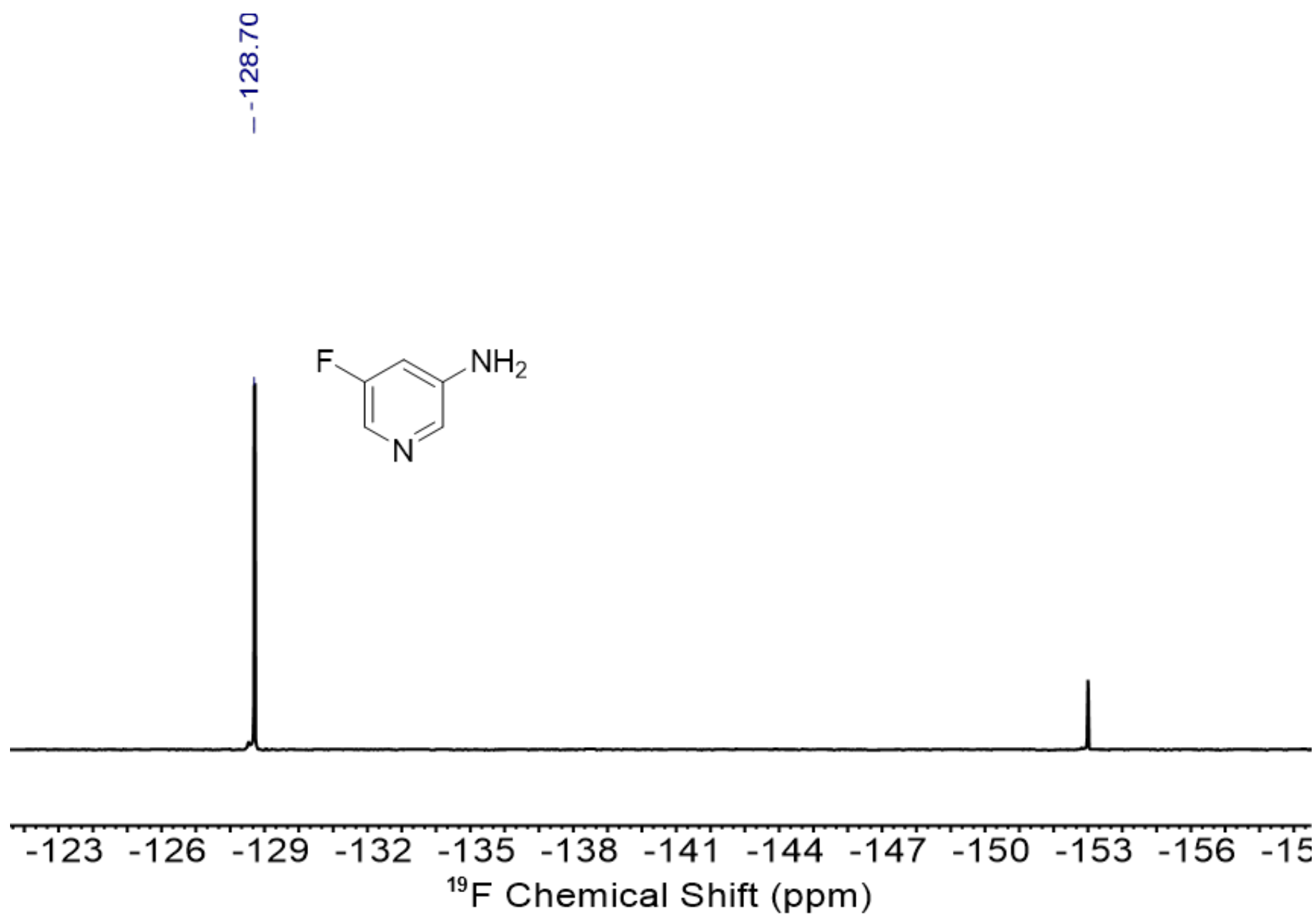

Fig. S6. ^{19}F NMR spectrum of substrate **b1**.

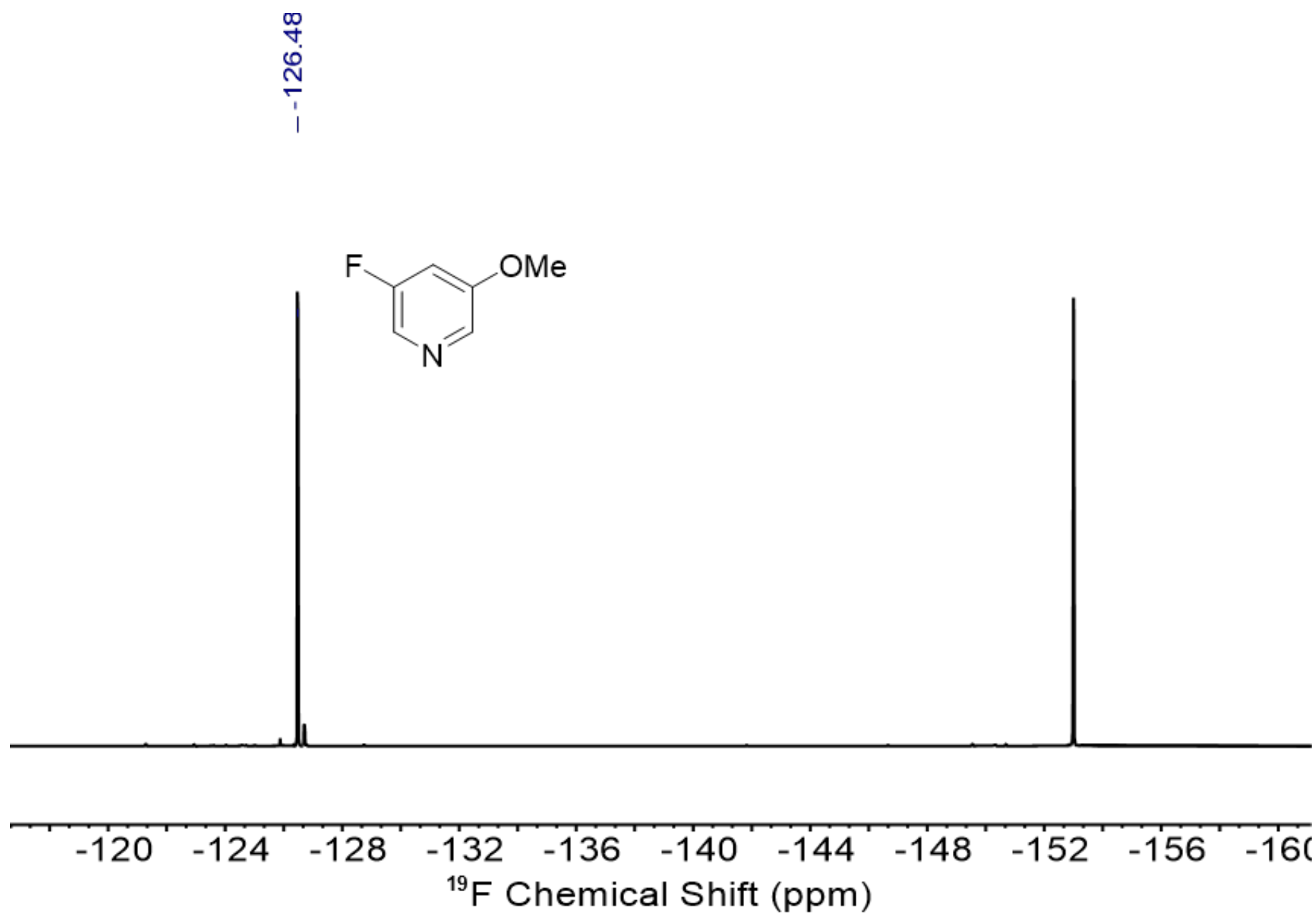

Fig. S7. ^{19}F NMR spectrum of substrate **b2**.

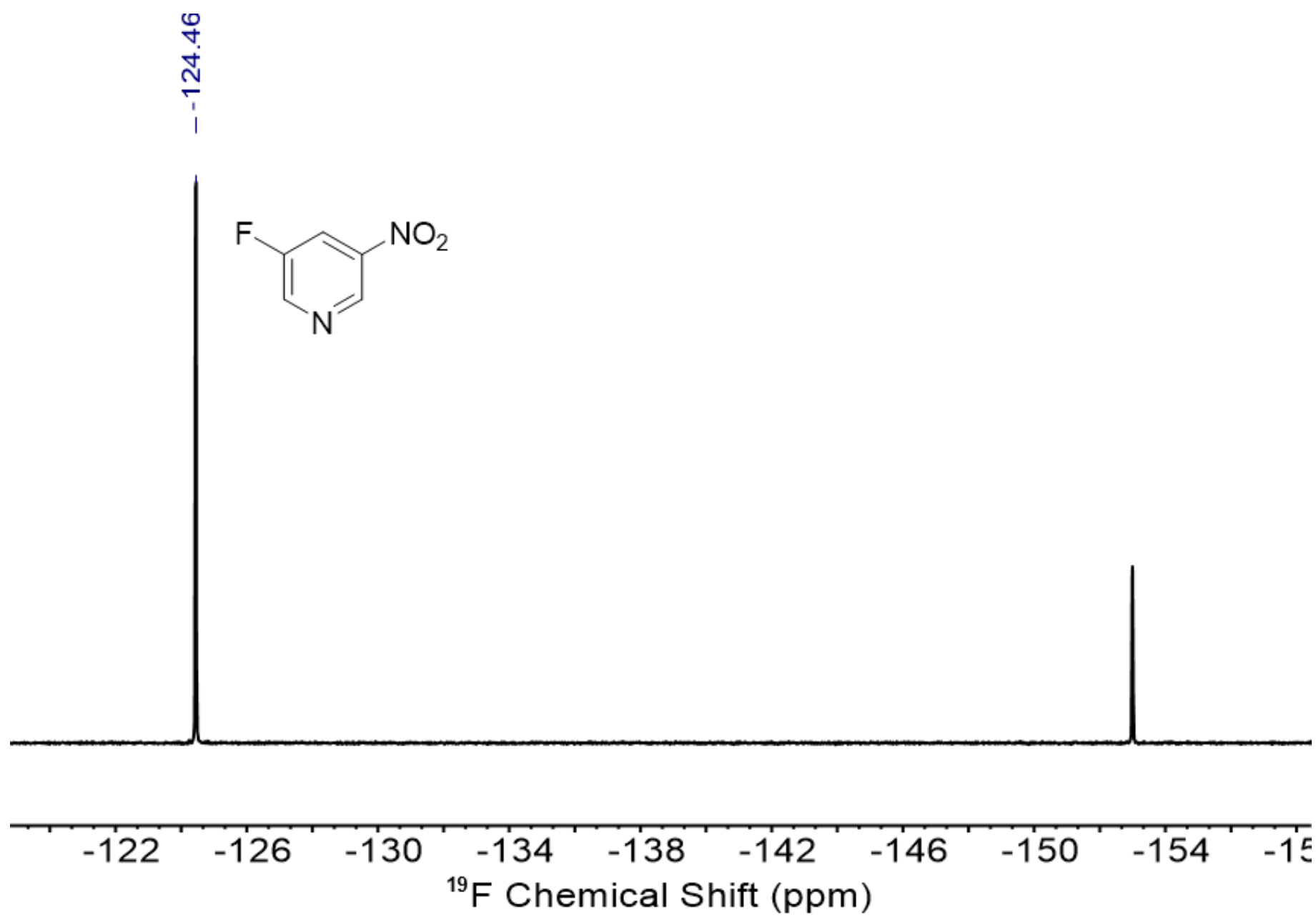

Fig. S8. ^{19}F NMR spectrum of substrate **b3**.

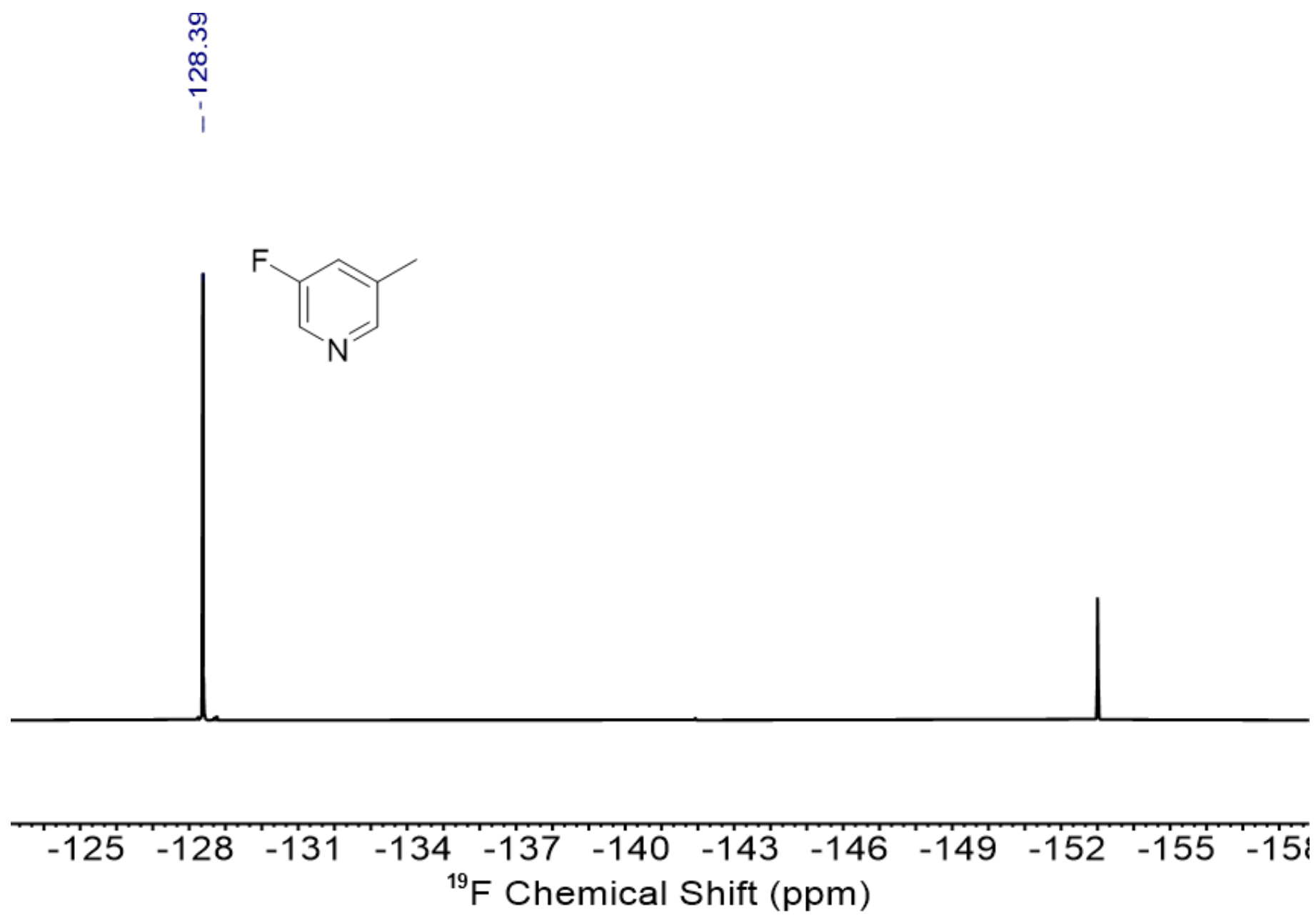

Fig. S9. ^{19}F NMR spectrum of substrate **b4**.

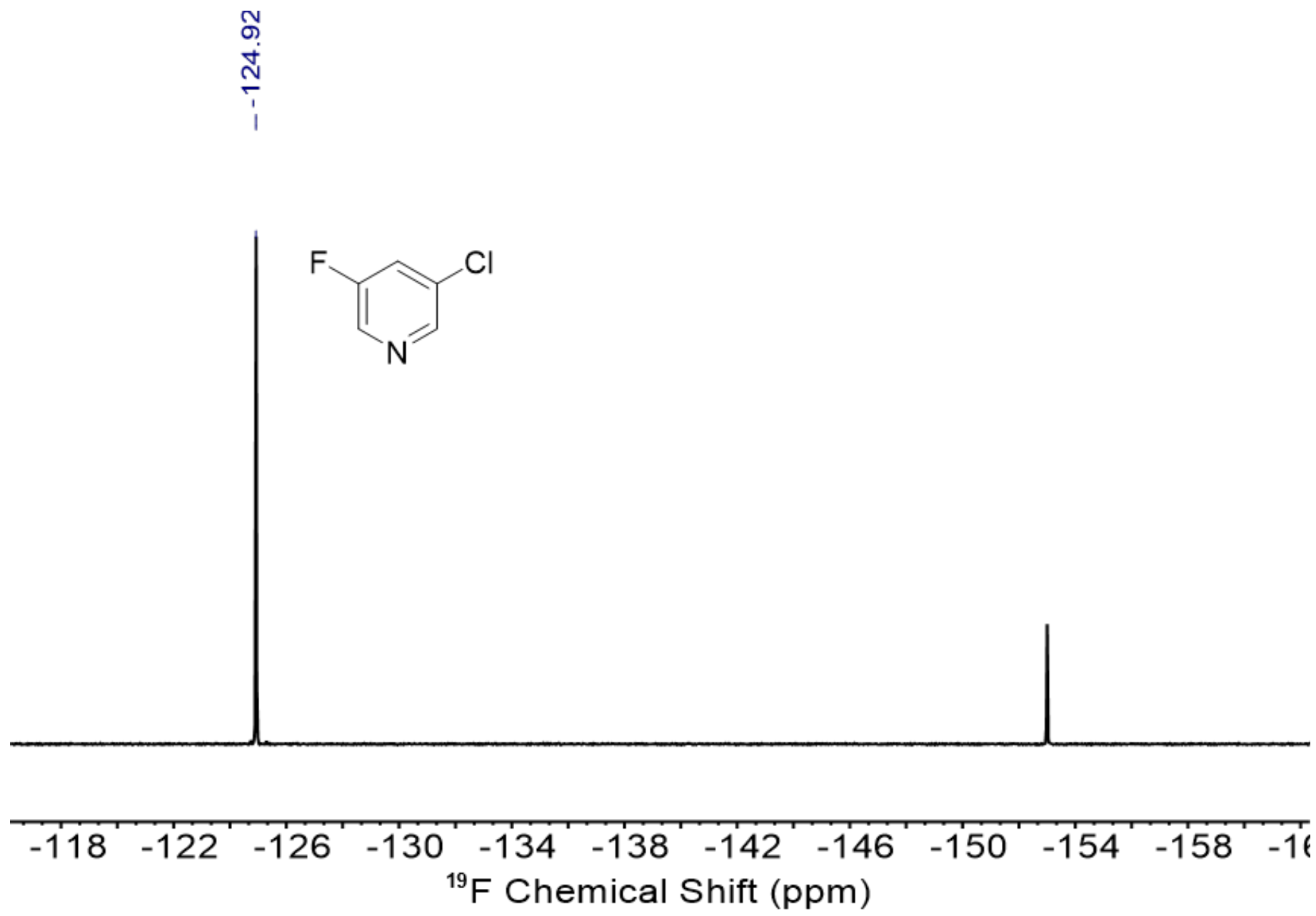

Fig. S10. ^{19}F NMR spectrum of substrate **b5**.

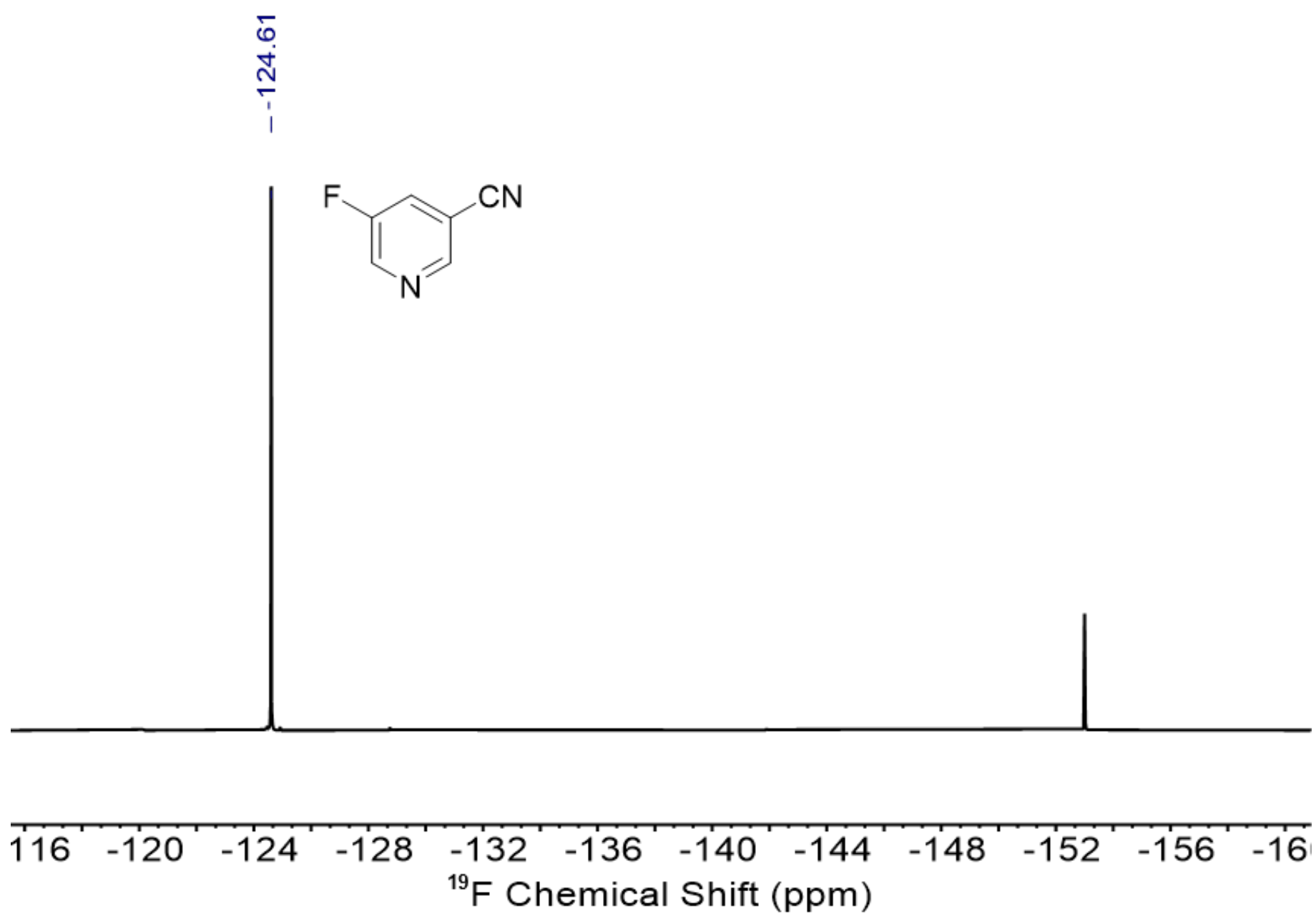

Fig. S11. ^{19}F NMR spectrum of substrate **b6**.

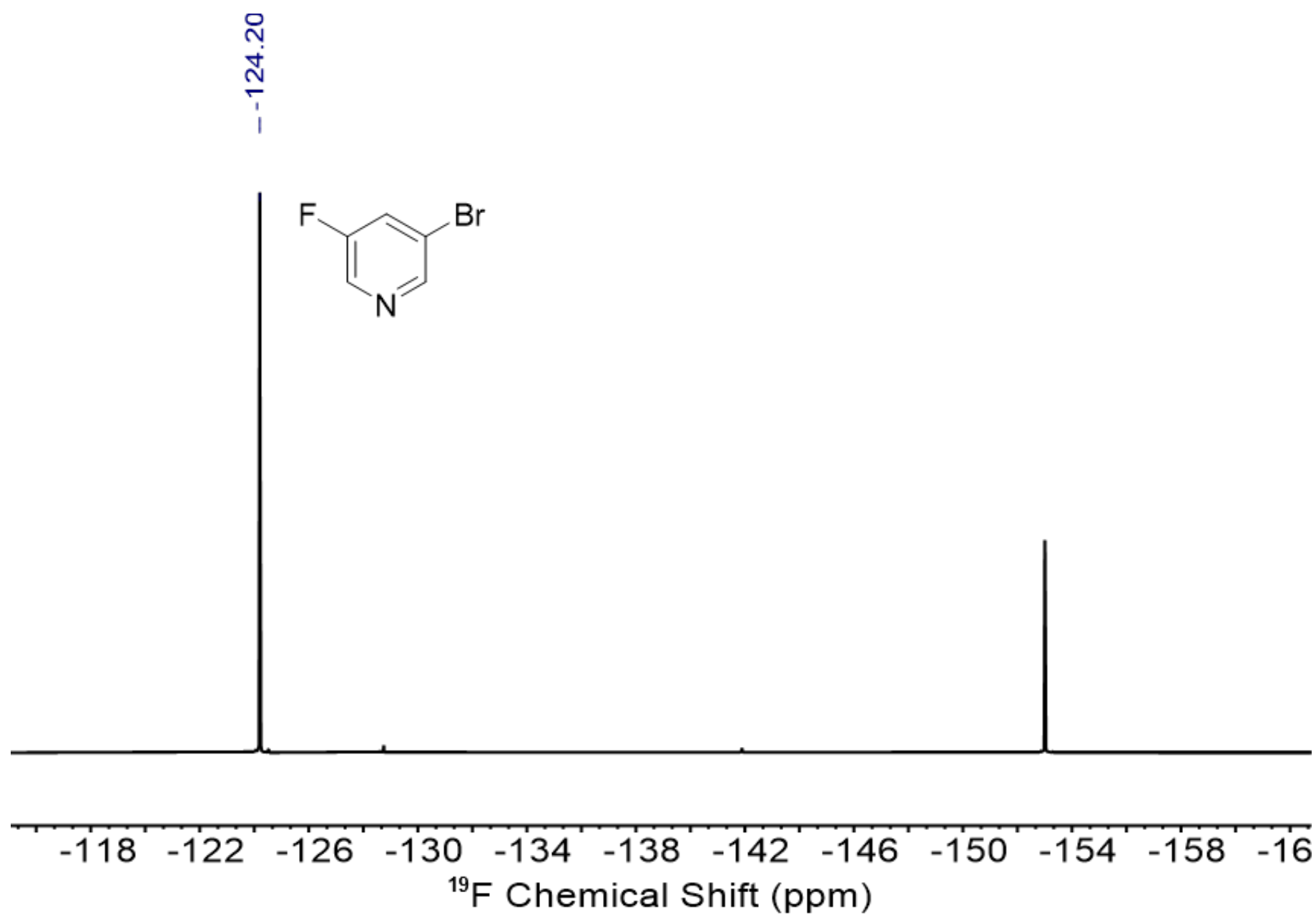

Fig. S12. ^{19}F NMR spectrum of substrate **b7**.

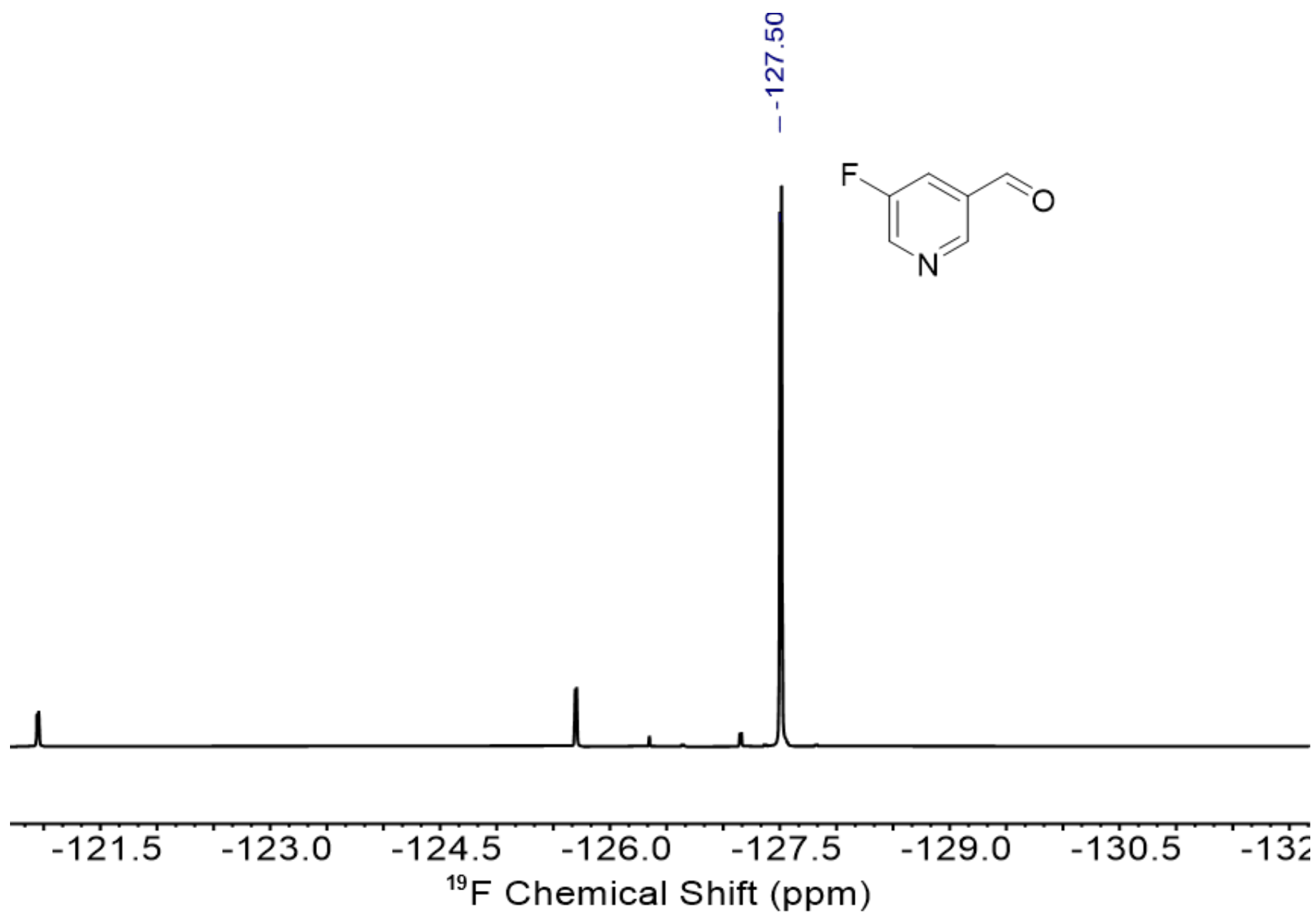

Fig. S13. ^{19}F NMR spectrum of substrate **b8**.

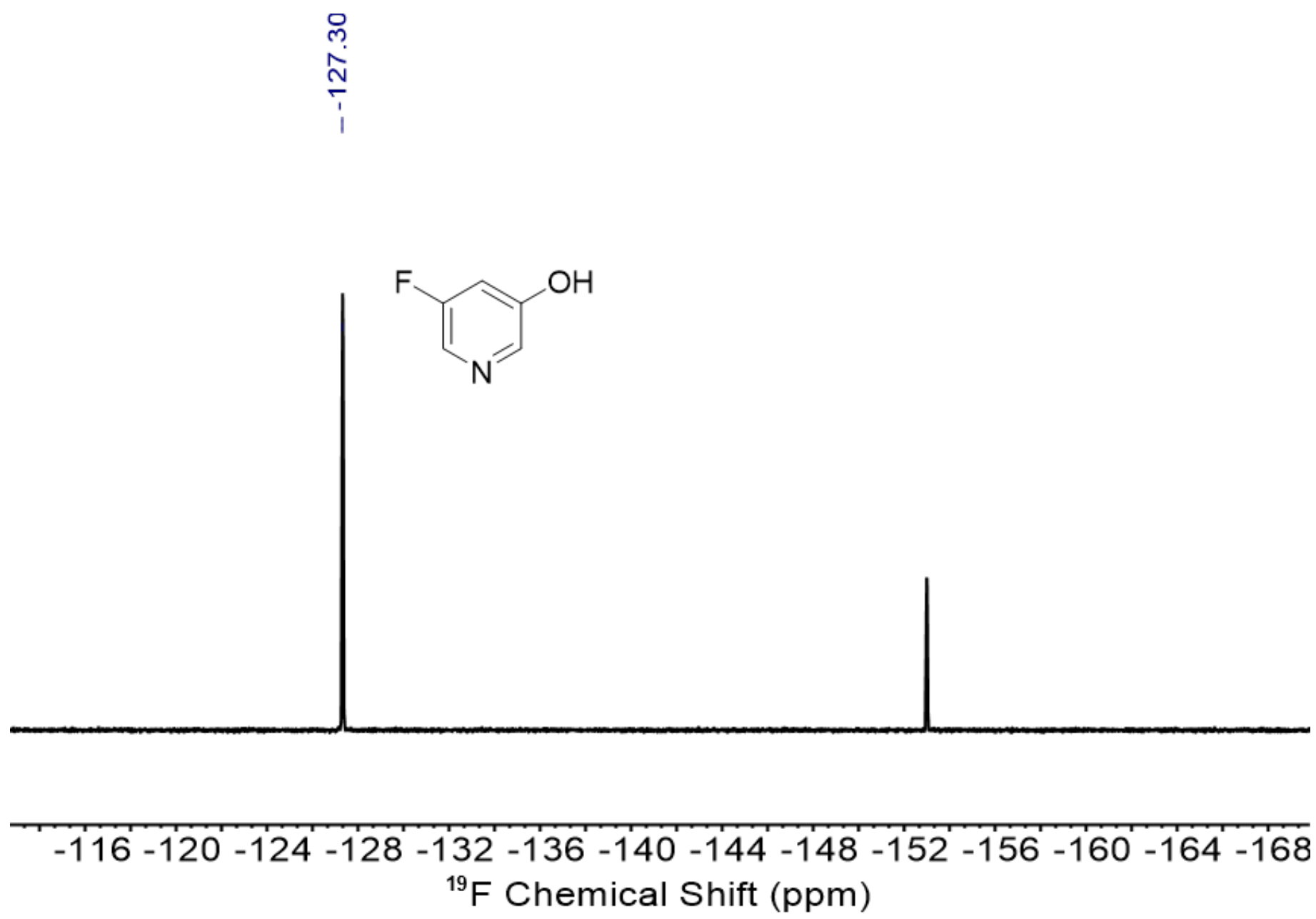

Fig. S14. ^{19}F NMR spectrum of substrate **b9**.

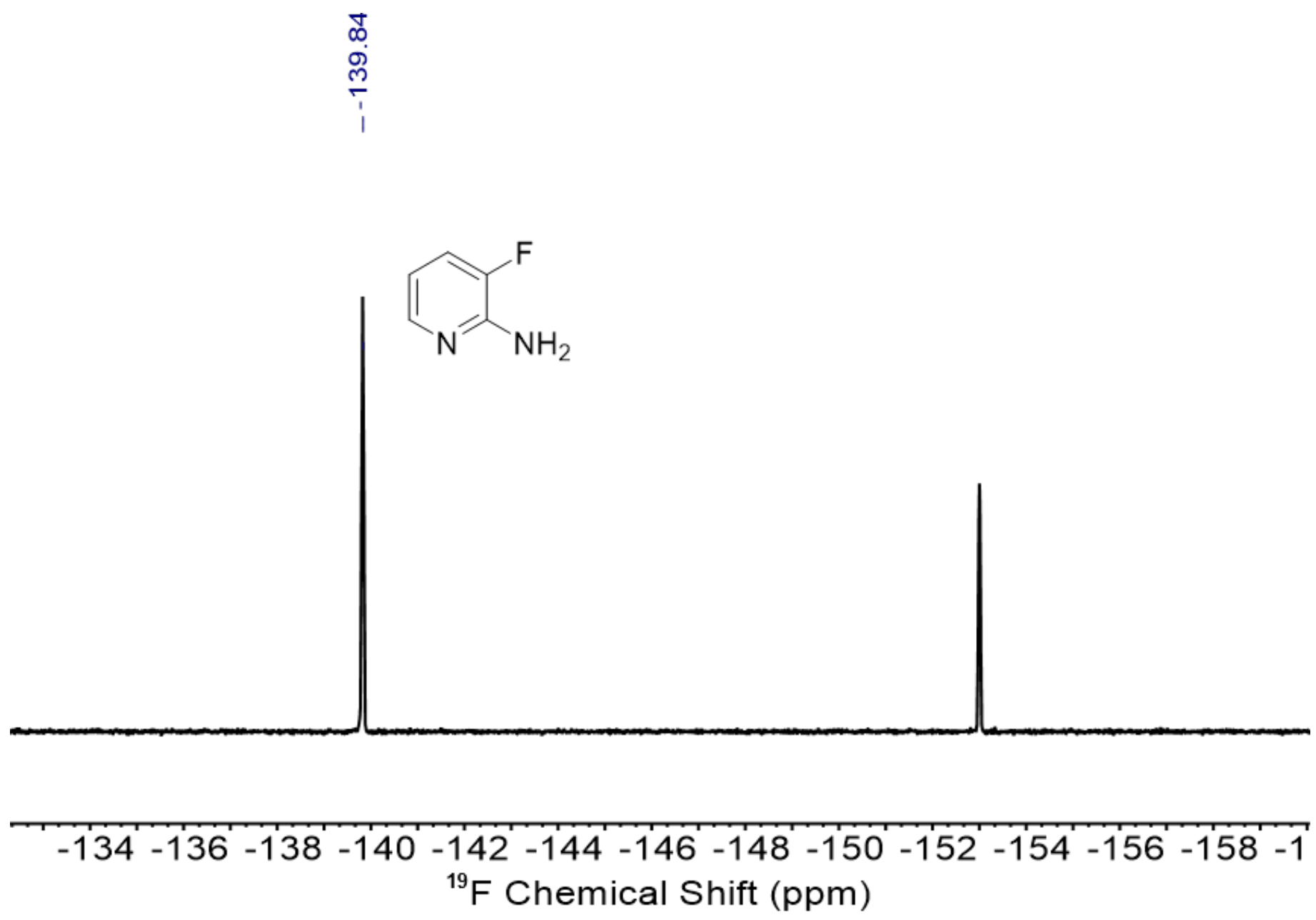

Fig. S15. ^{19}F NMR spectrum of substrate **c1**.

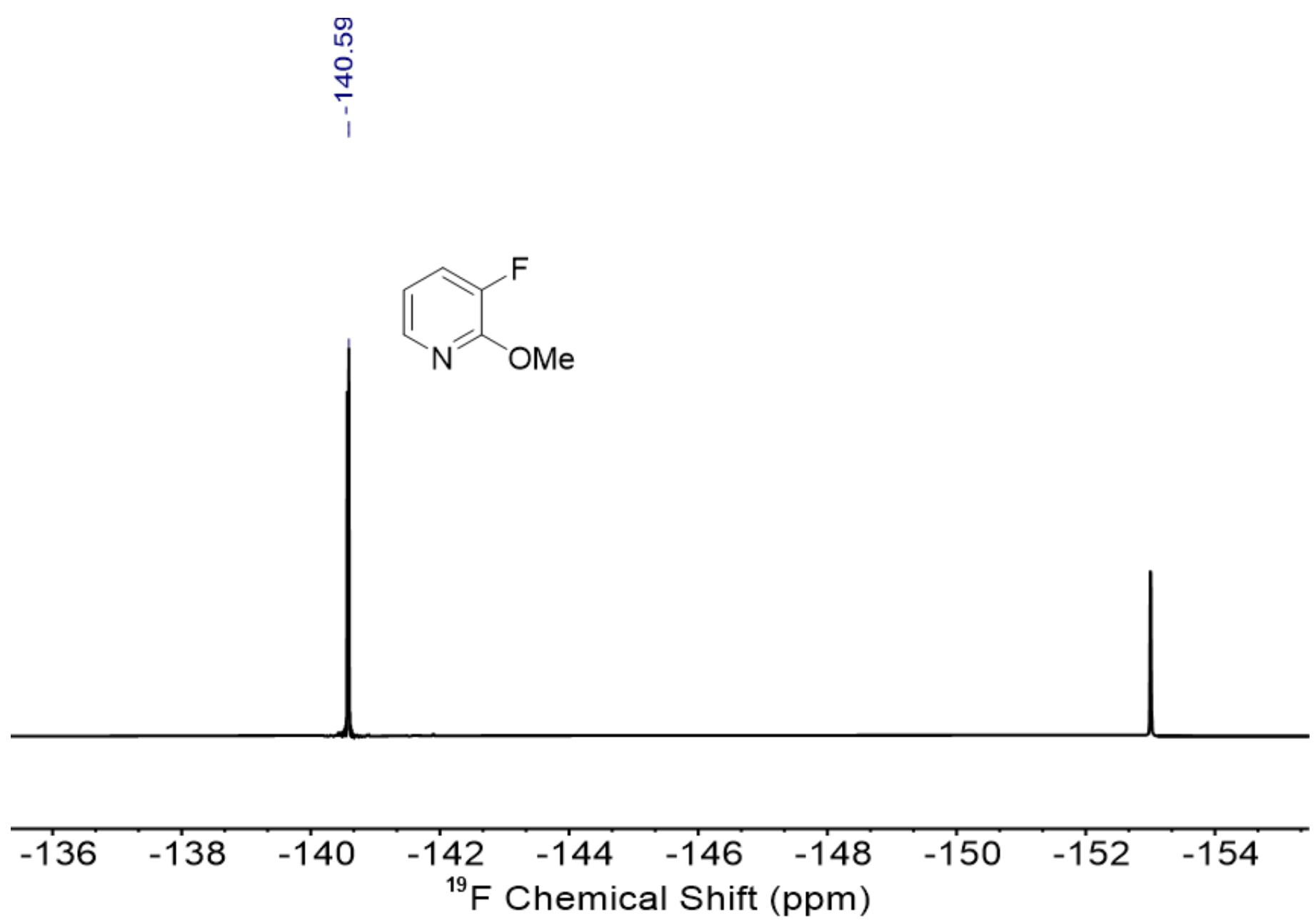

Fig. S16. ^{19}F NMR spectrum of substrate **c2**.

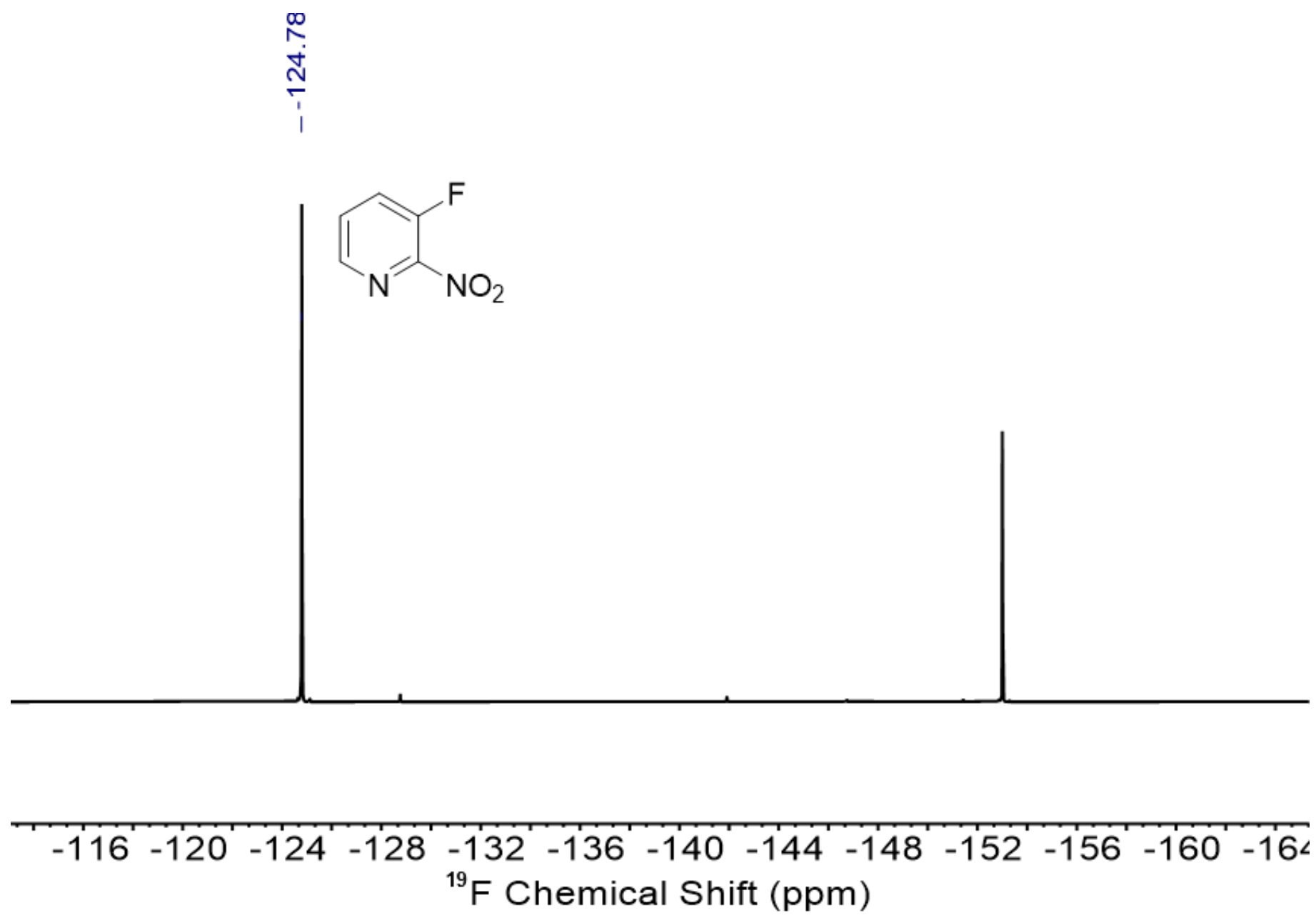

Fig. S17. ^{19}F NMR spectrum of substrate **c3**.

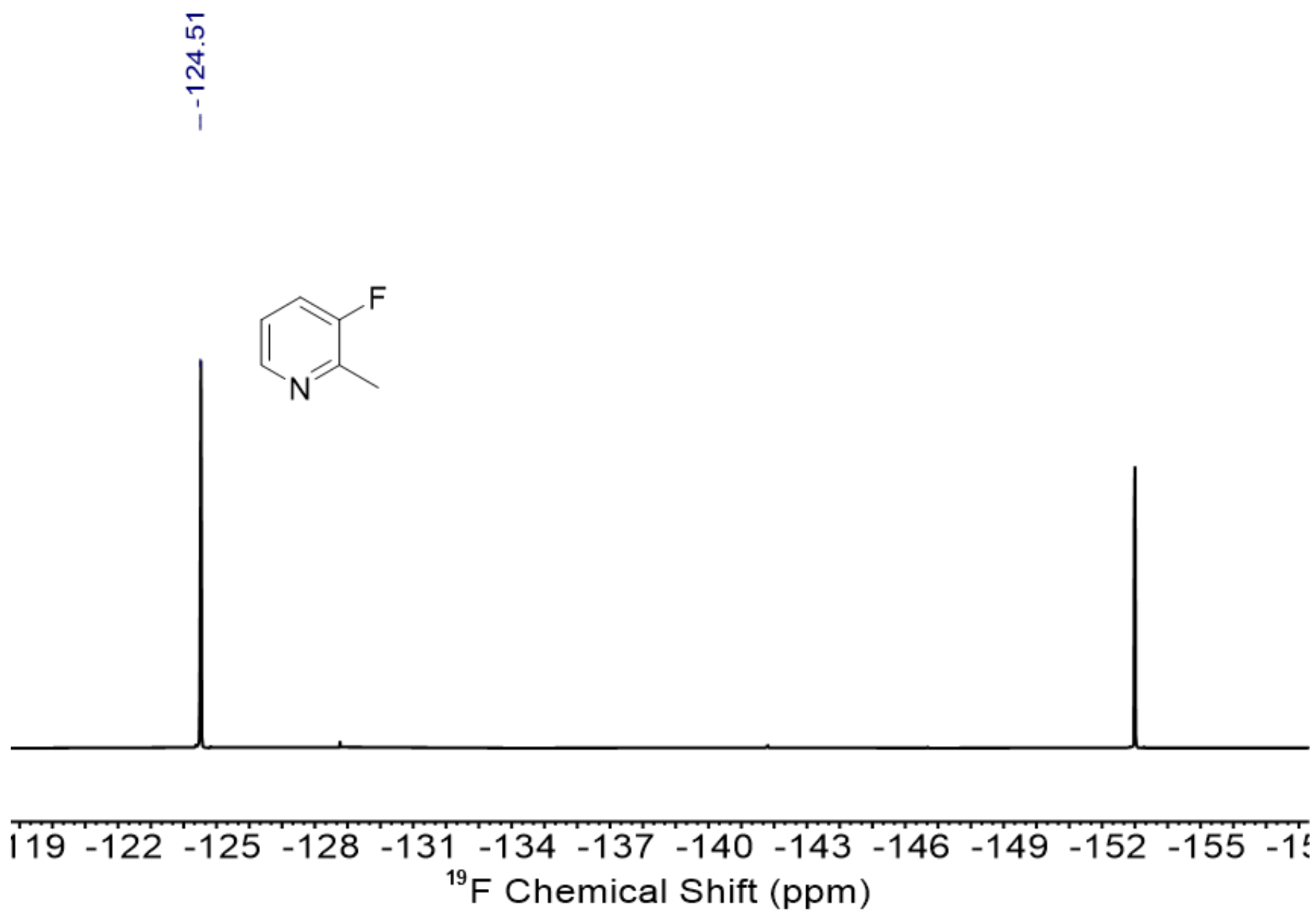

Fig. S18. ^{19}F NMR spectrum of substrate **c4**.

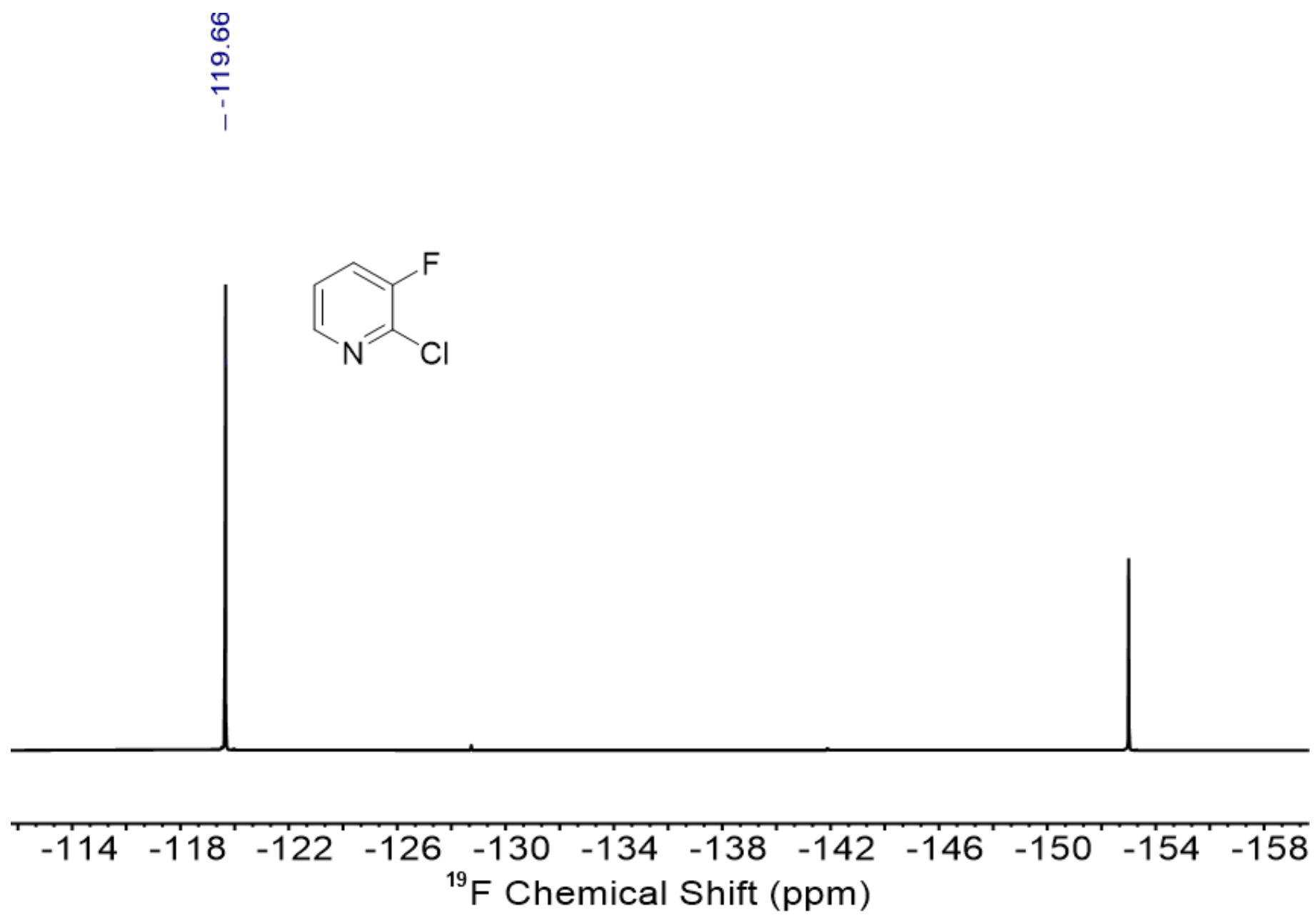

Fig. S19. ^{19}F NMR spectrum of substrate **c5**.

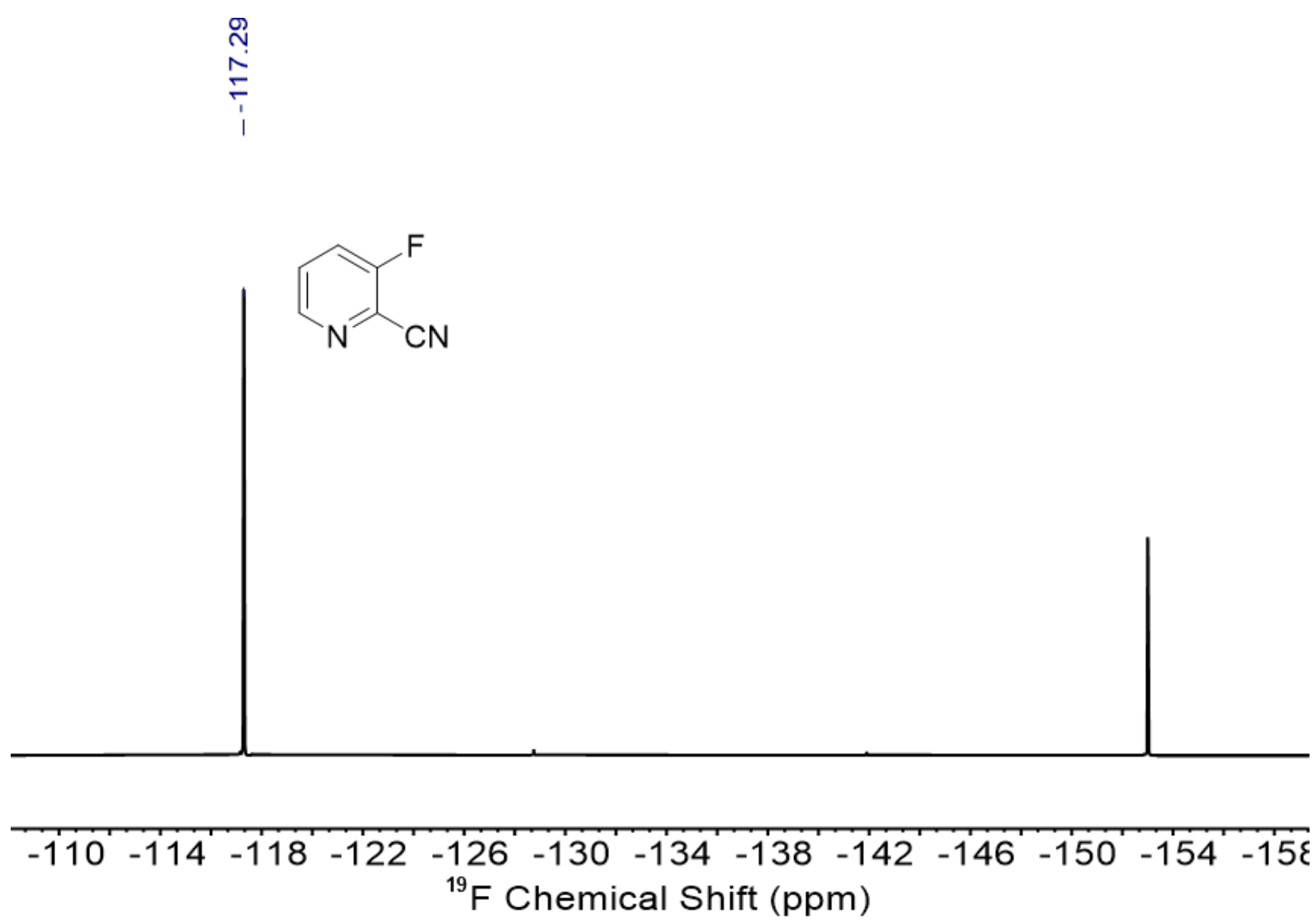

Fig. S20. ^{19}F NMR spectrum of substrate **c6**.

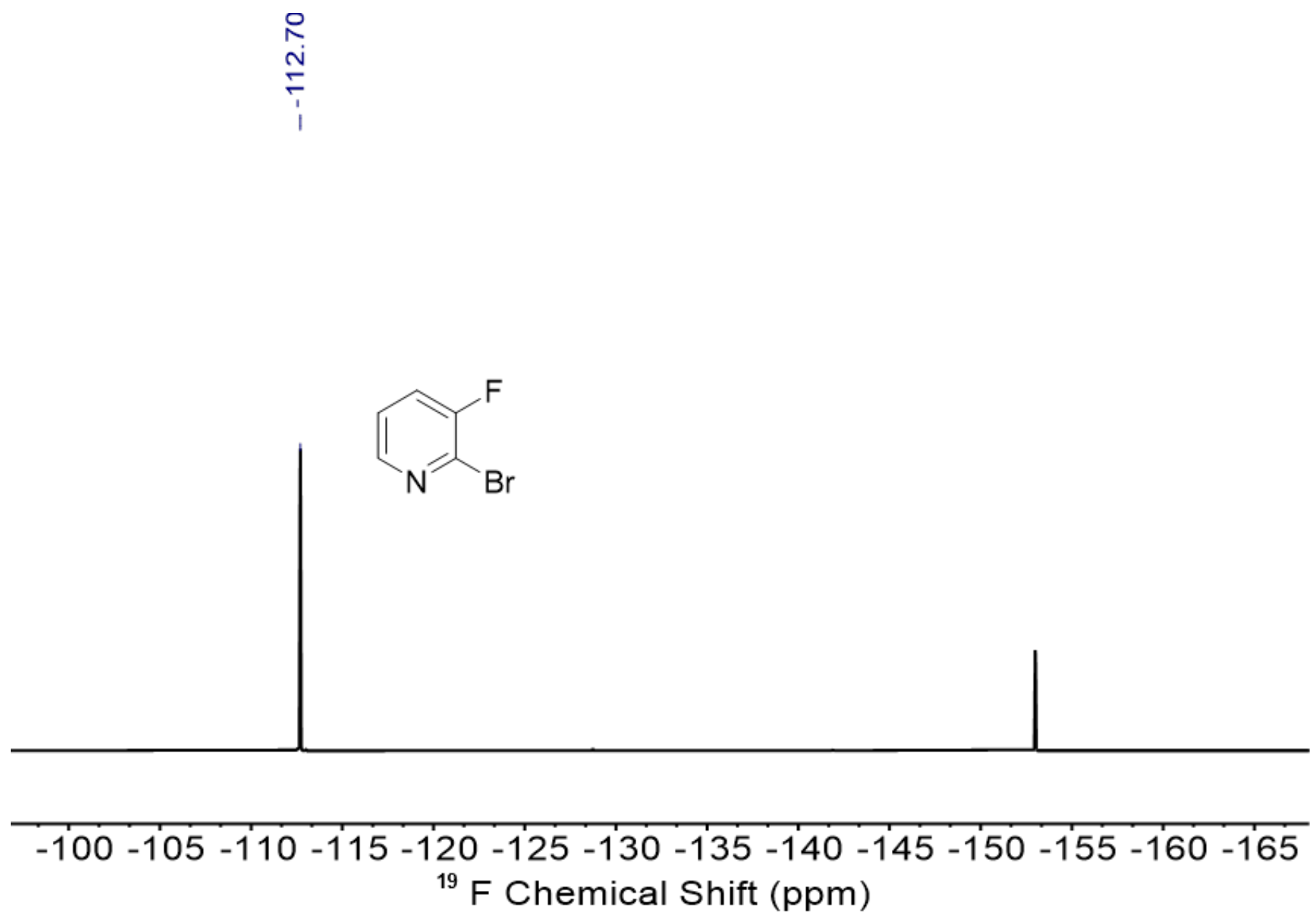

Fig. S21. ^{19}F NMR spectrum of substrate **c7**.

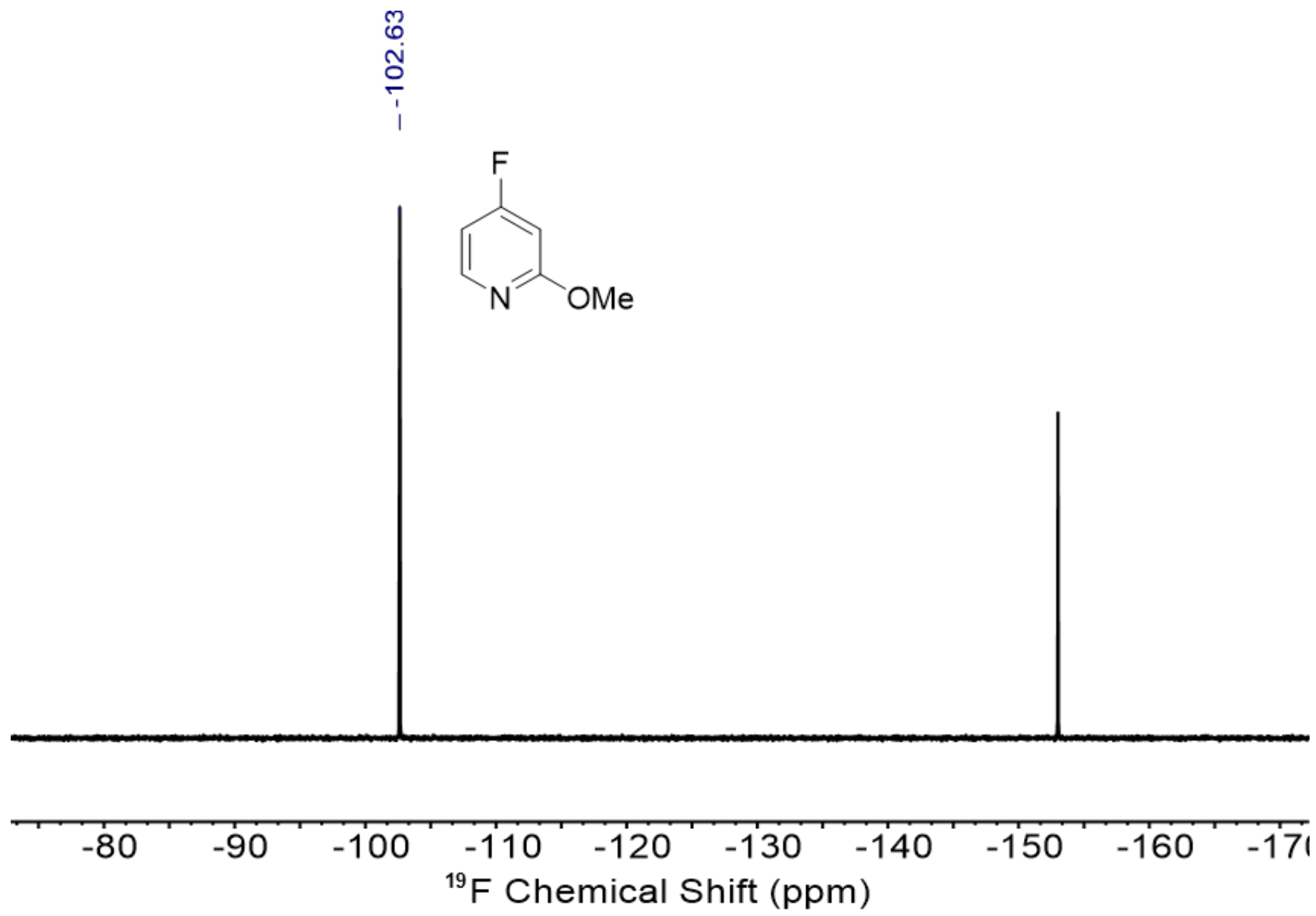

Fig. S22. ^{19}F NMR spectrum of substrate **d1**.

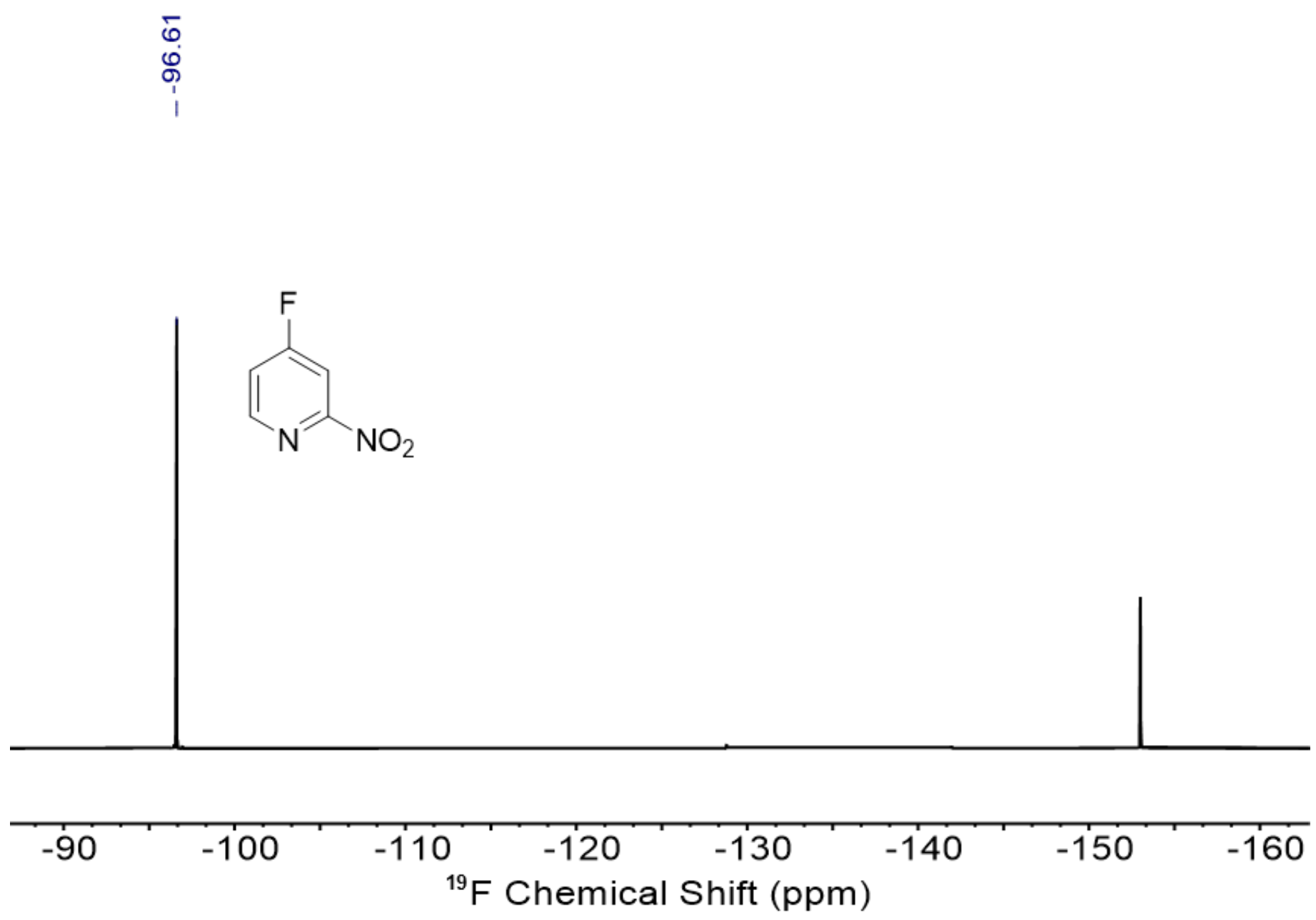

Fig. S23. ^{19}F NMR spectrum of substrate **d2**.

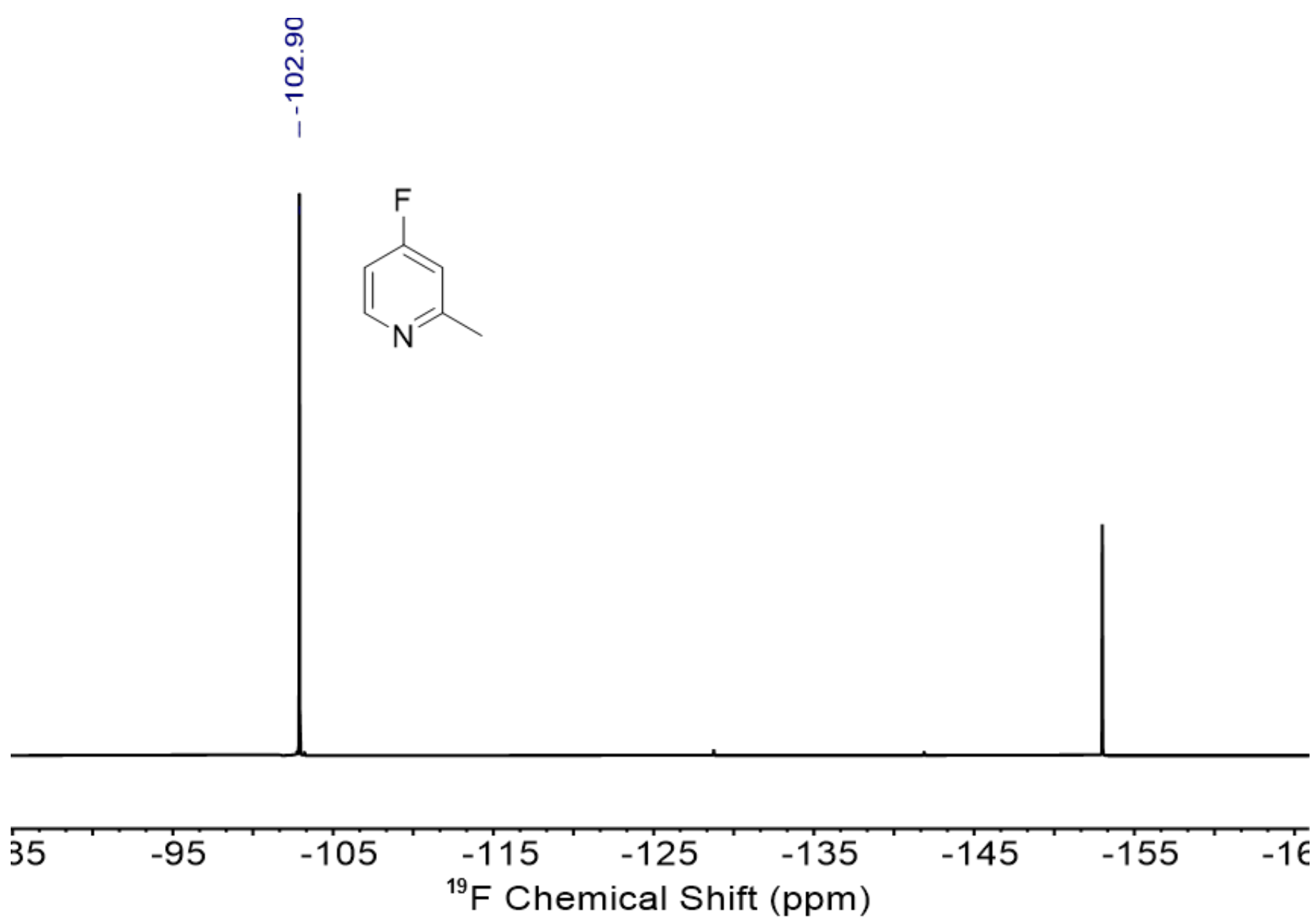

Fig. S24. ^{19}F NMR spectrum of substrate **d3**.

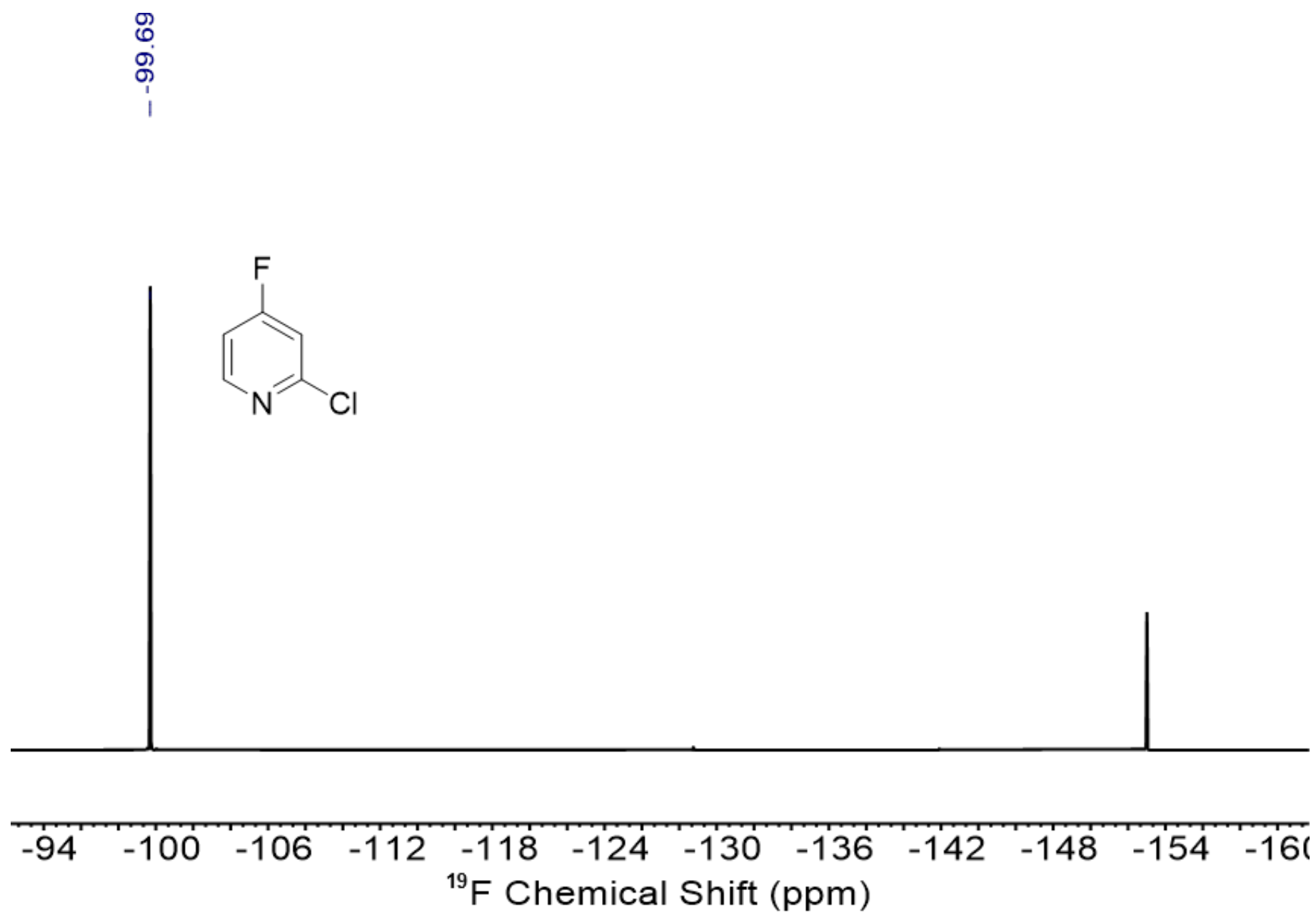

Fig. S25. ^{19}F NMR spectrum of substrate **d4**.

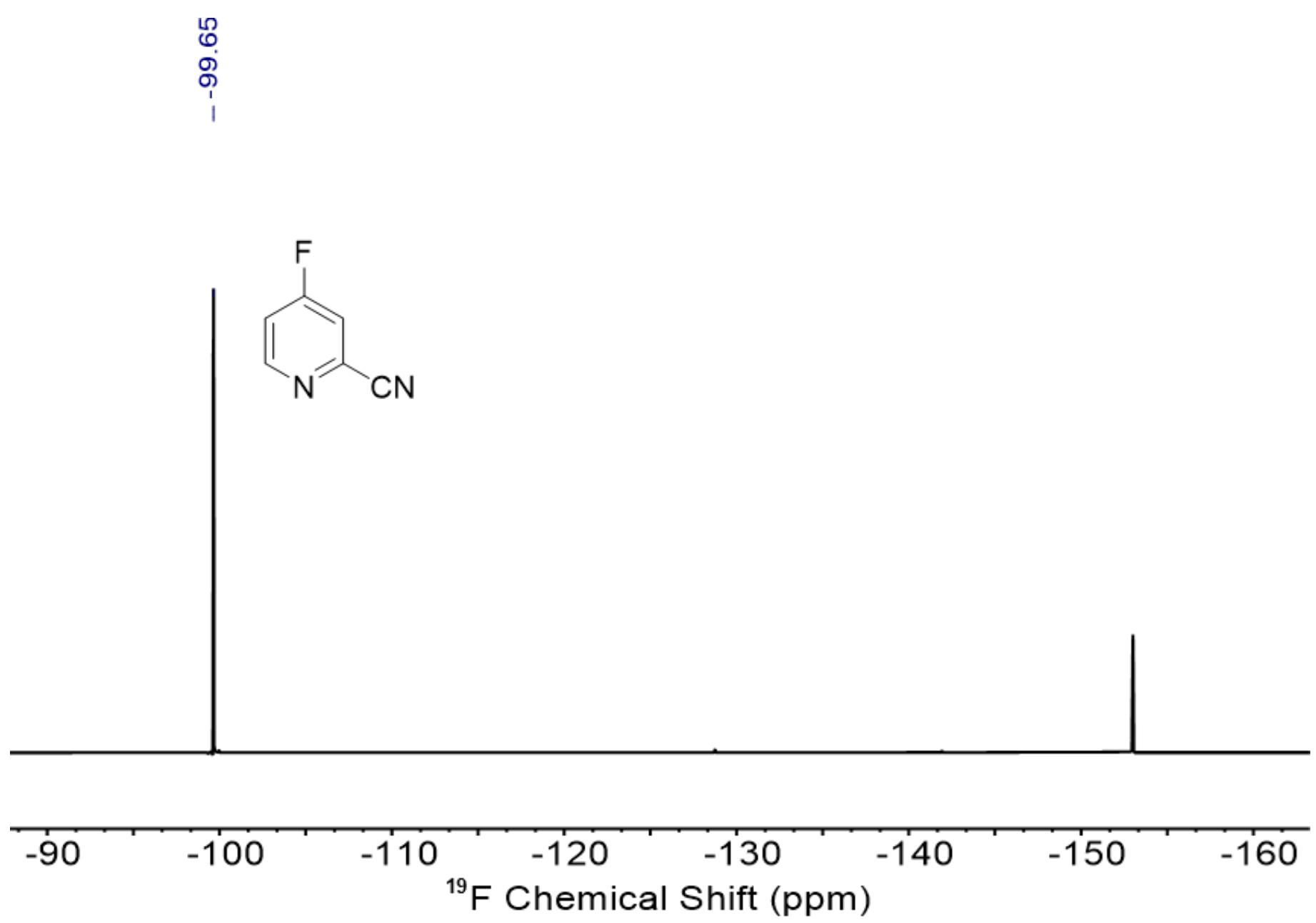

Fig. S26. ^{19}F NMR spectrum of substrate **d5**.

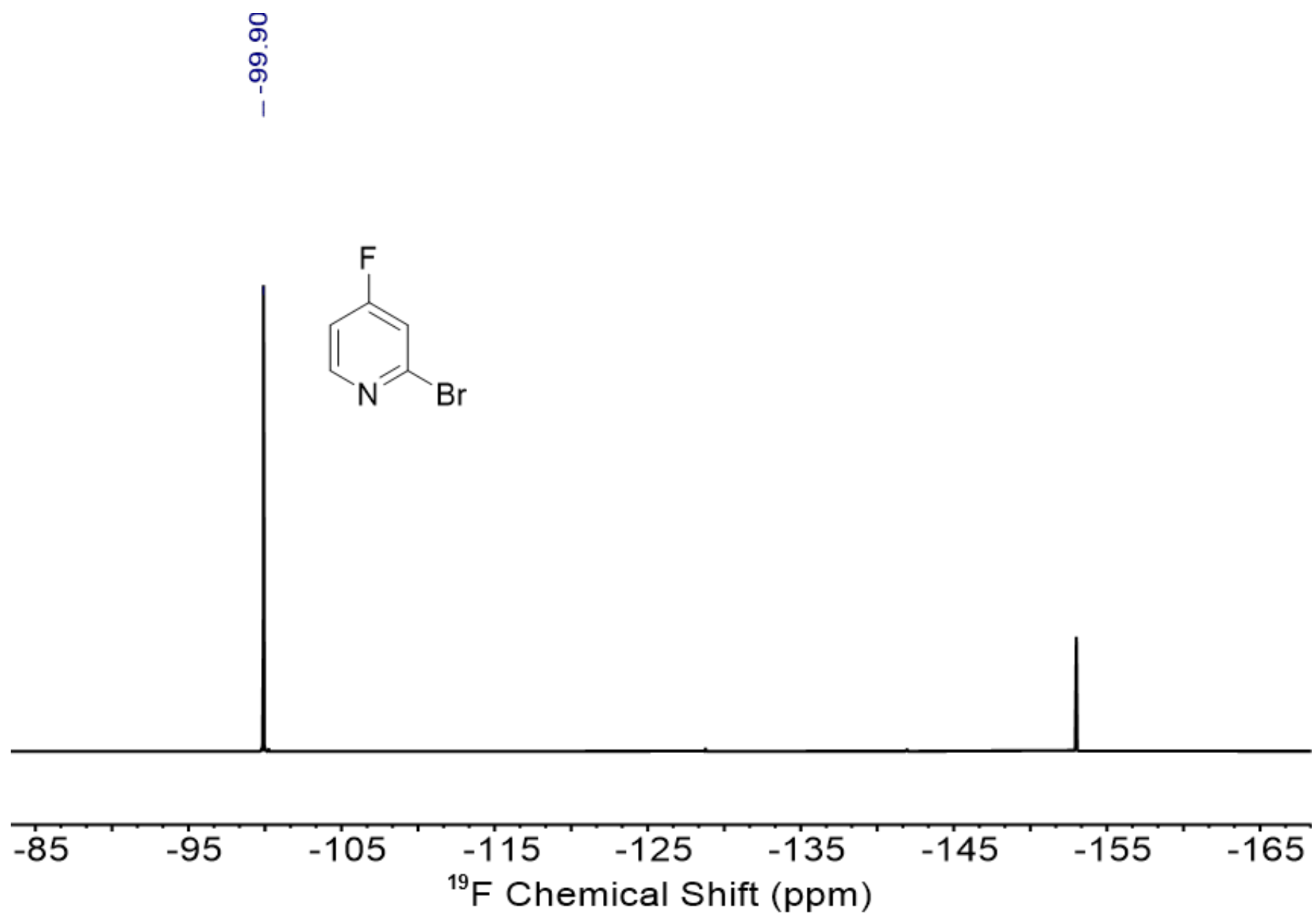

Fig. S27. ^{19}F NMR spectrum of substrate **d6**.

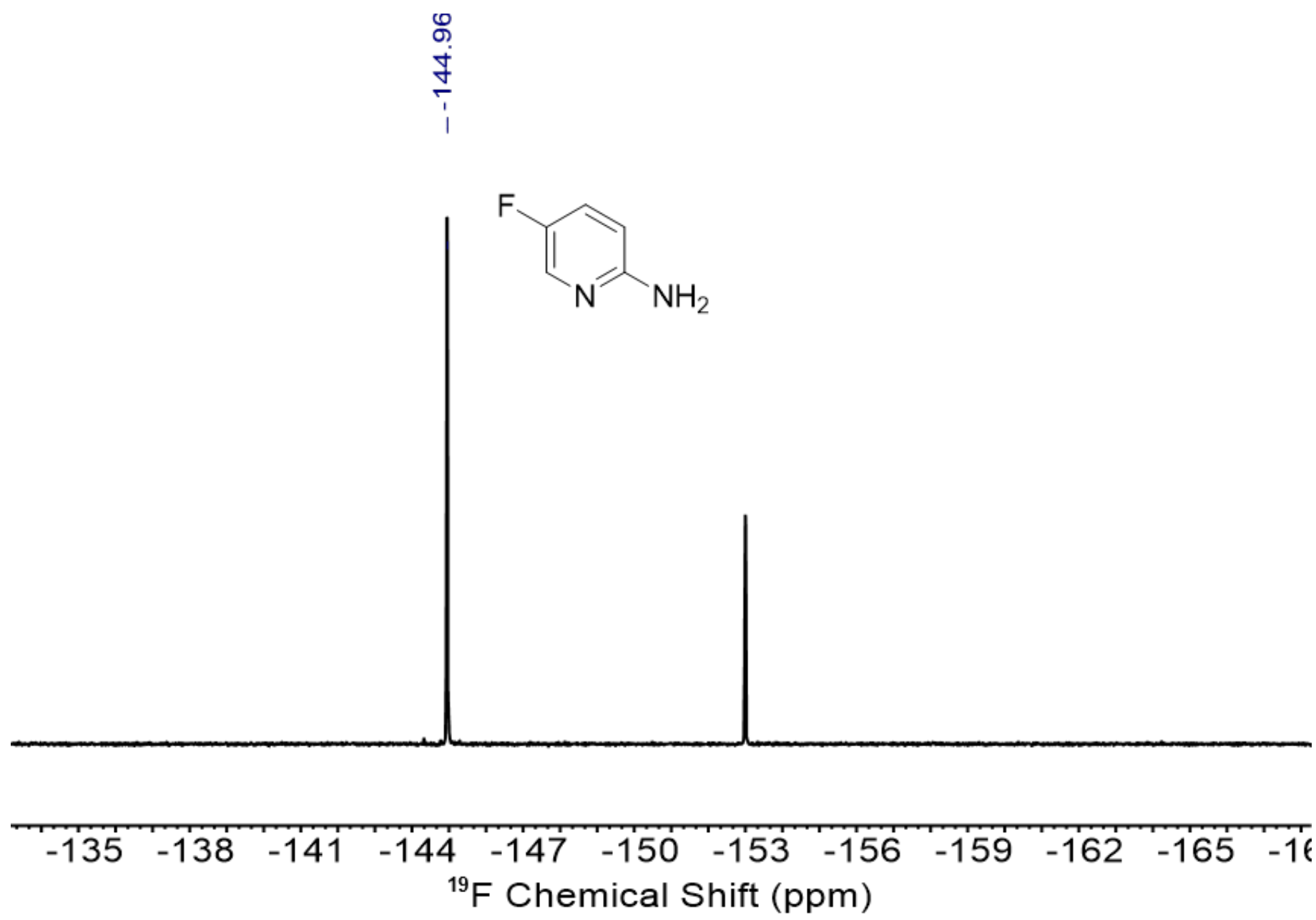

Fig. S28. ^{19}F NMR spectrum of substrate **e1**.

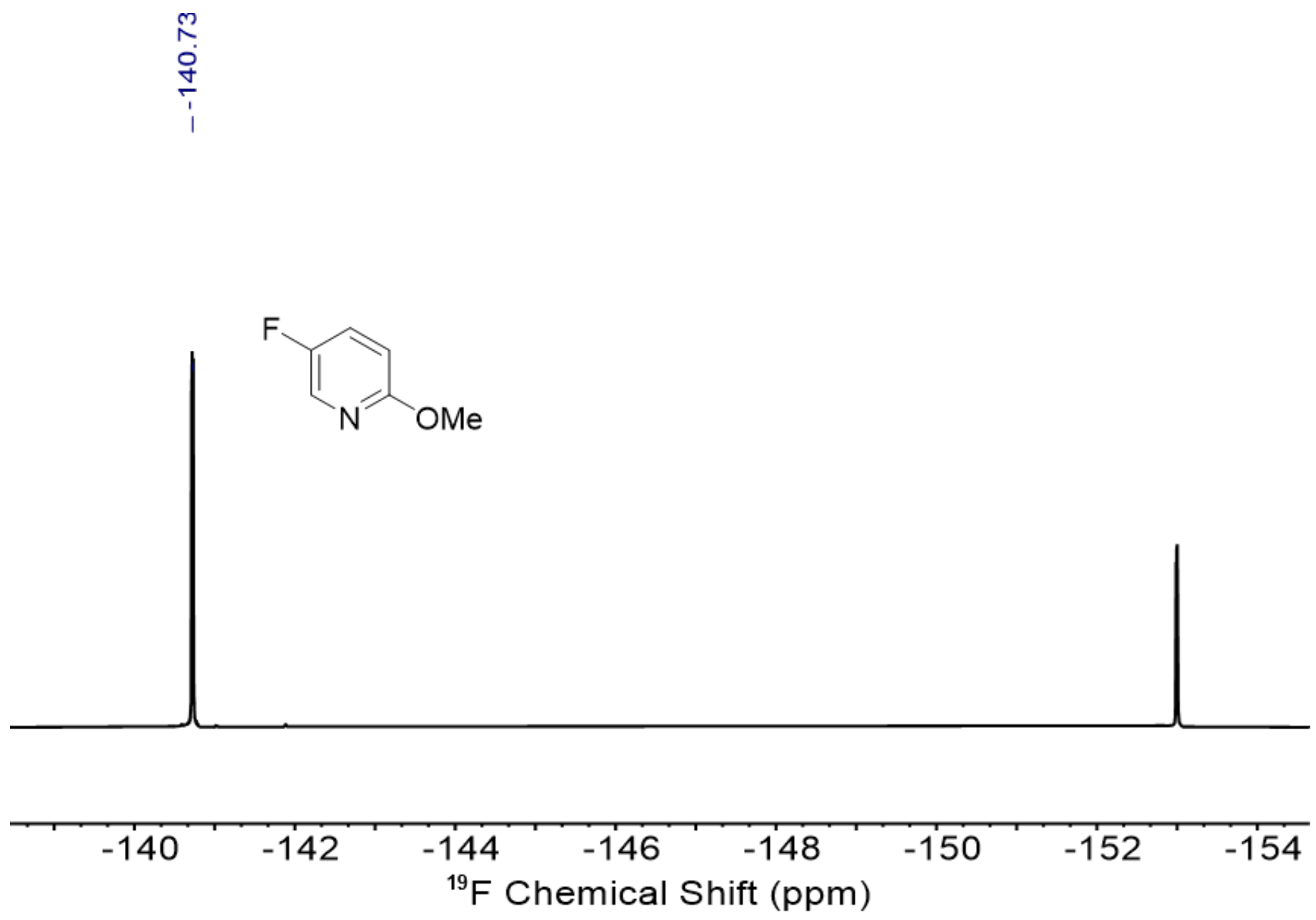

Fig. S29. ^{19}F NMR spectrum of substrate **e2**.

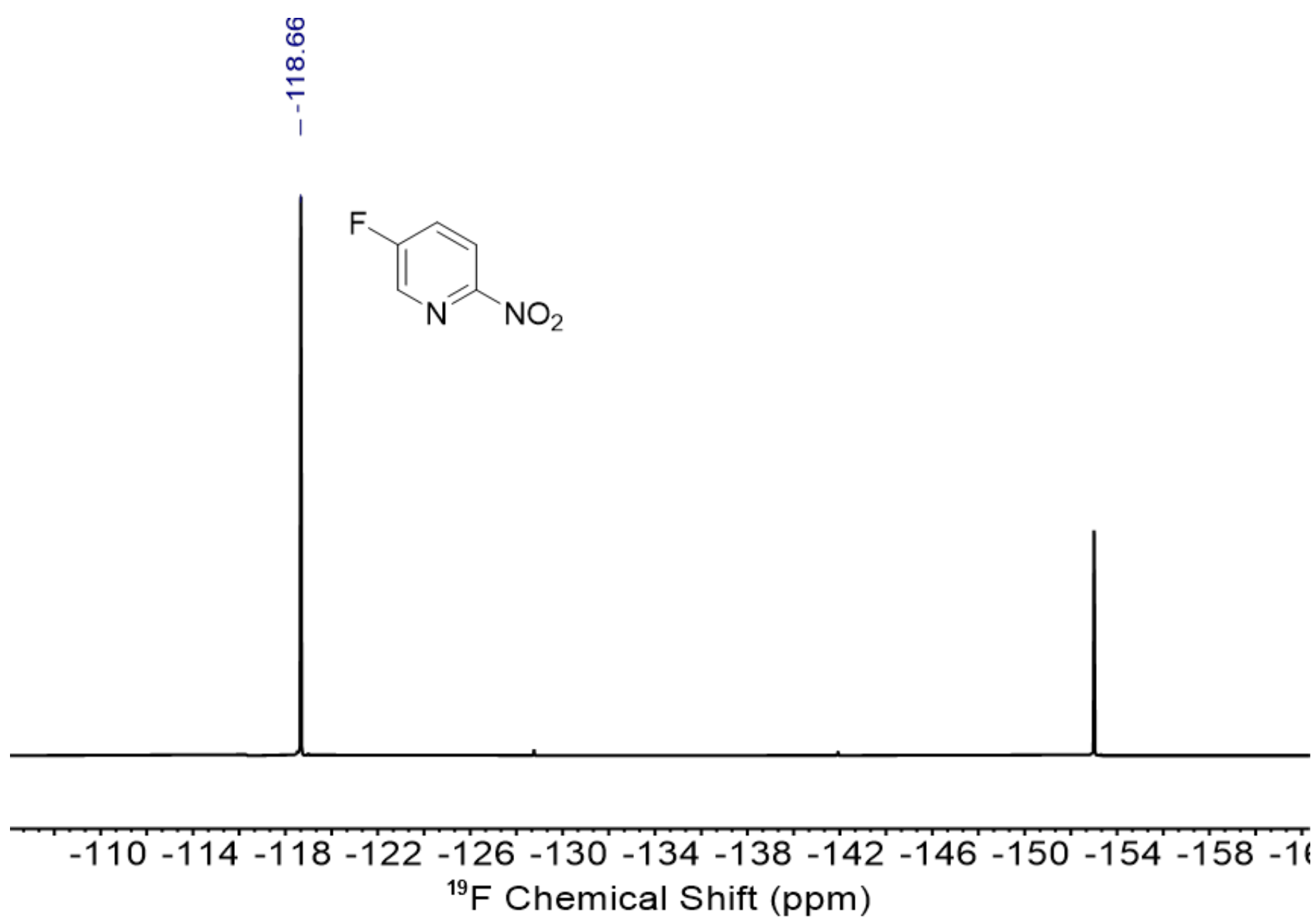

Fig. S30. ^{19}F NMR spectrum of substrate **e3**.

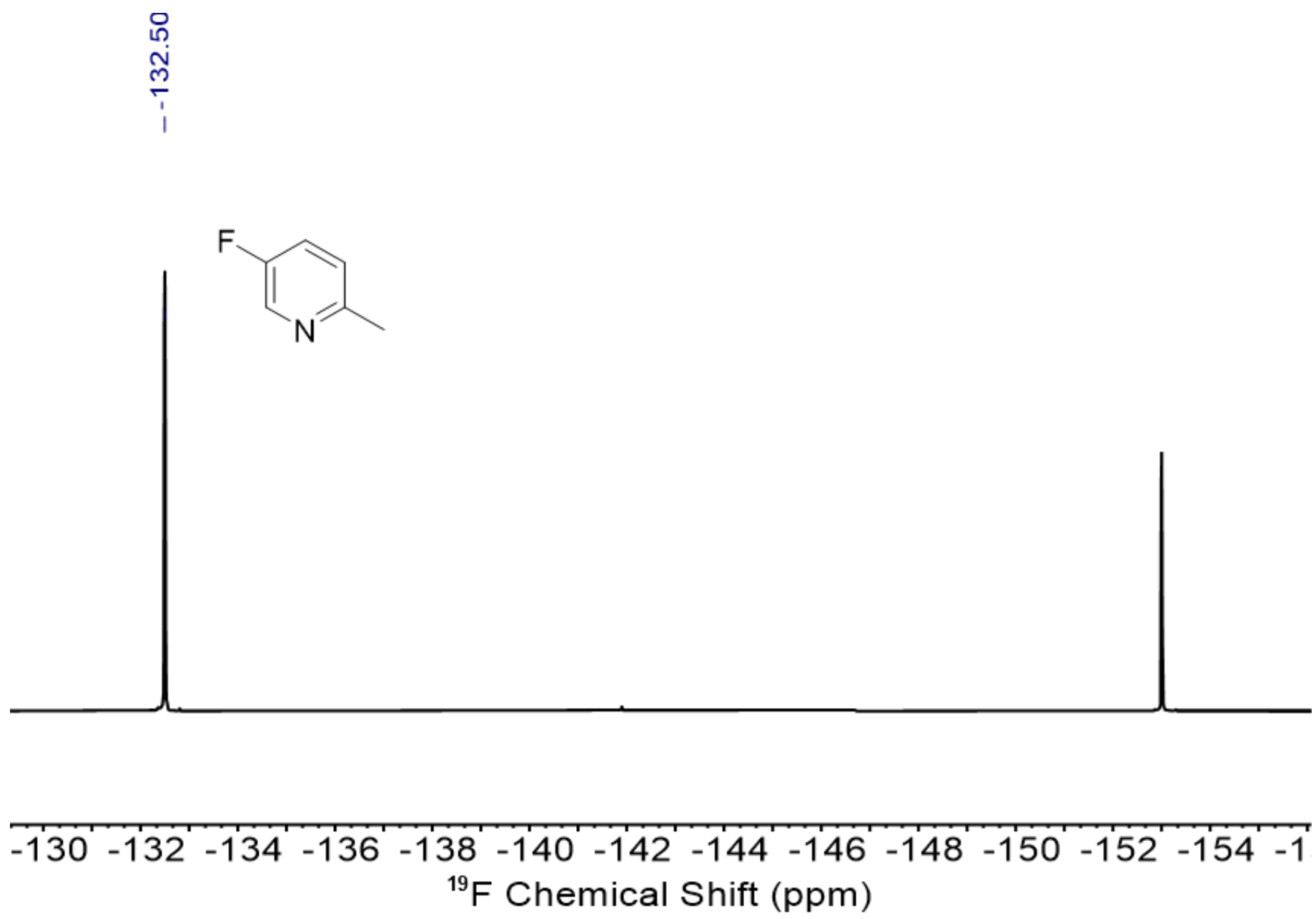

Fig. S31. ^{19}F NMR spectrum of substrate **e4**.

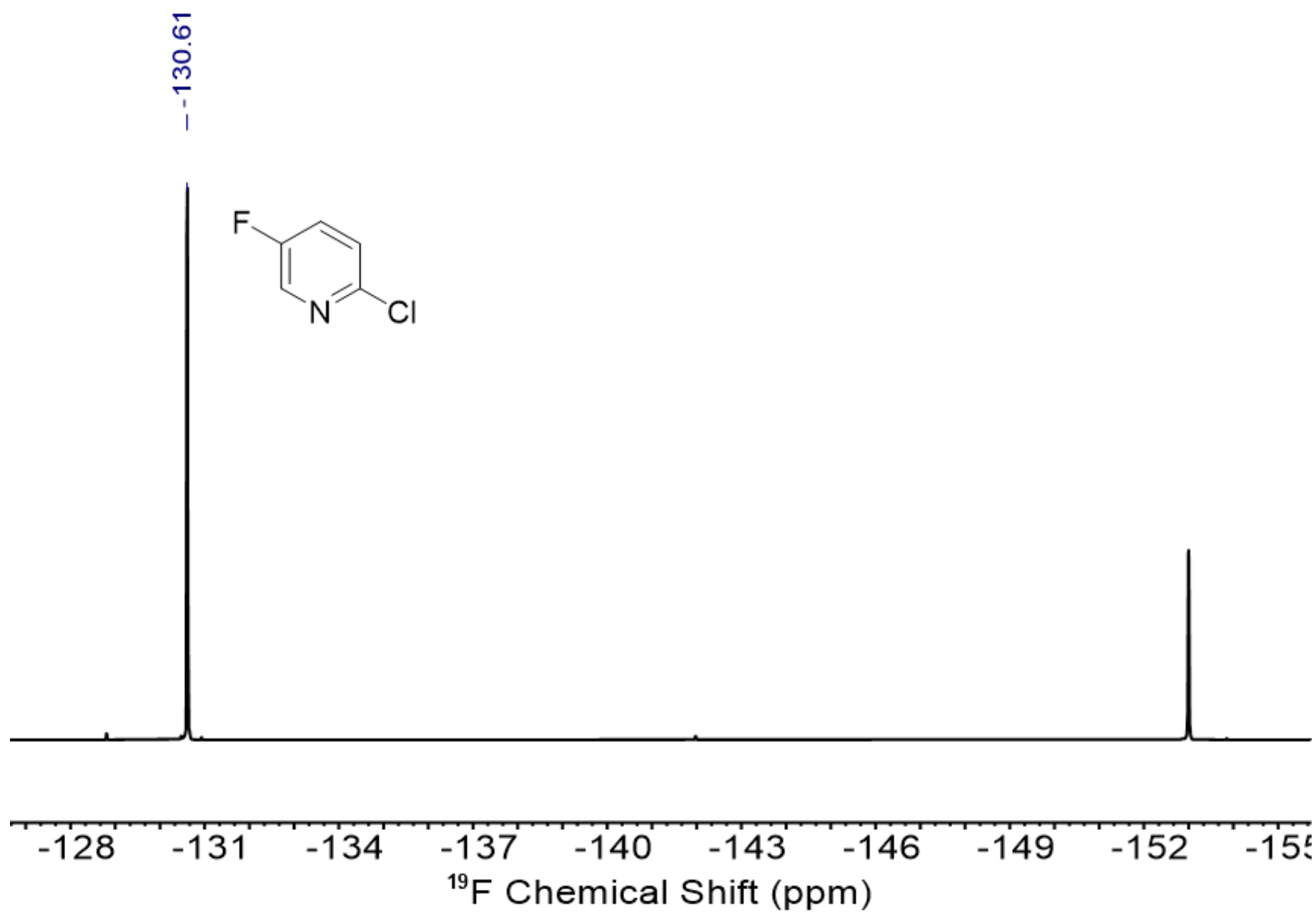

Fig. S32. ^{19}F NMR spectrum of substrate **e5**.

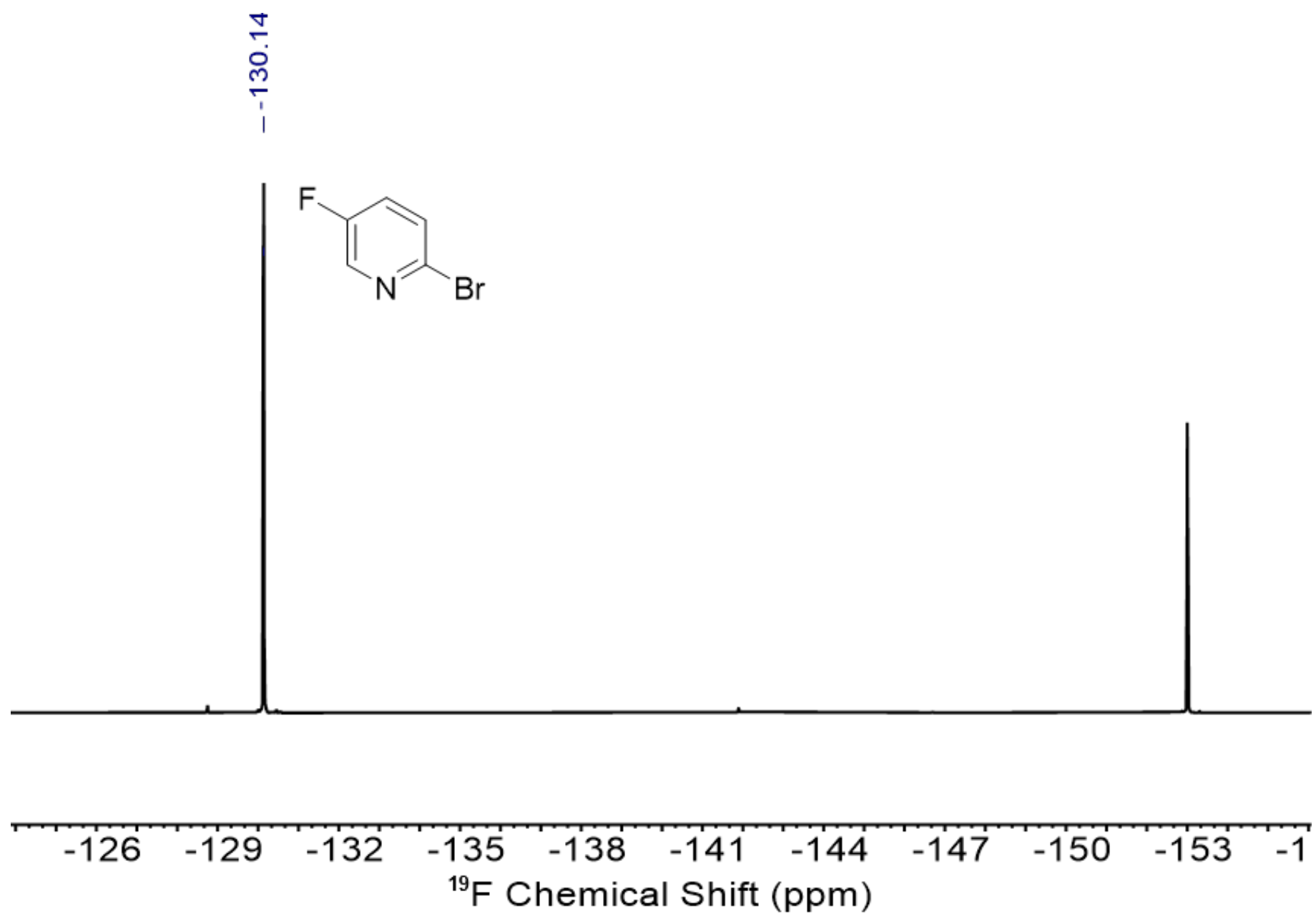

Fig. S33. ^{19}F NMR spectrum of substrate **e6**.

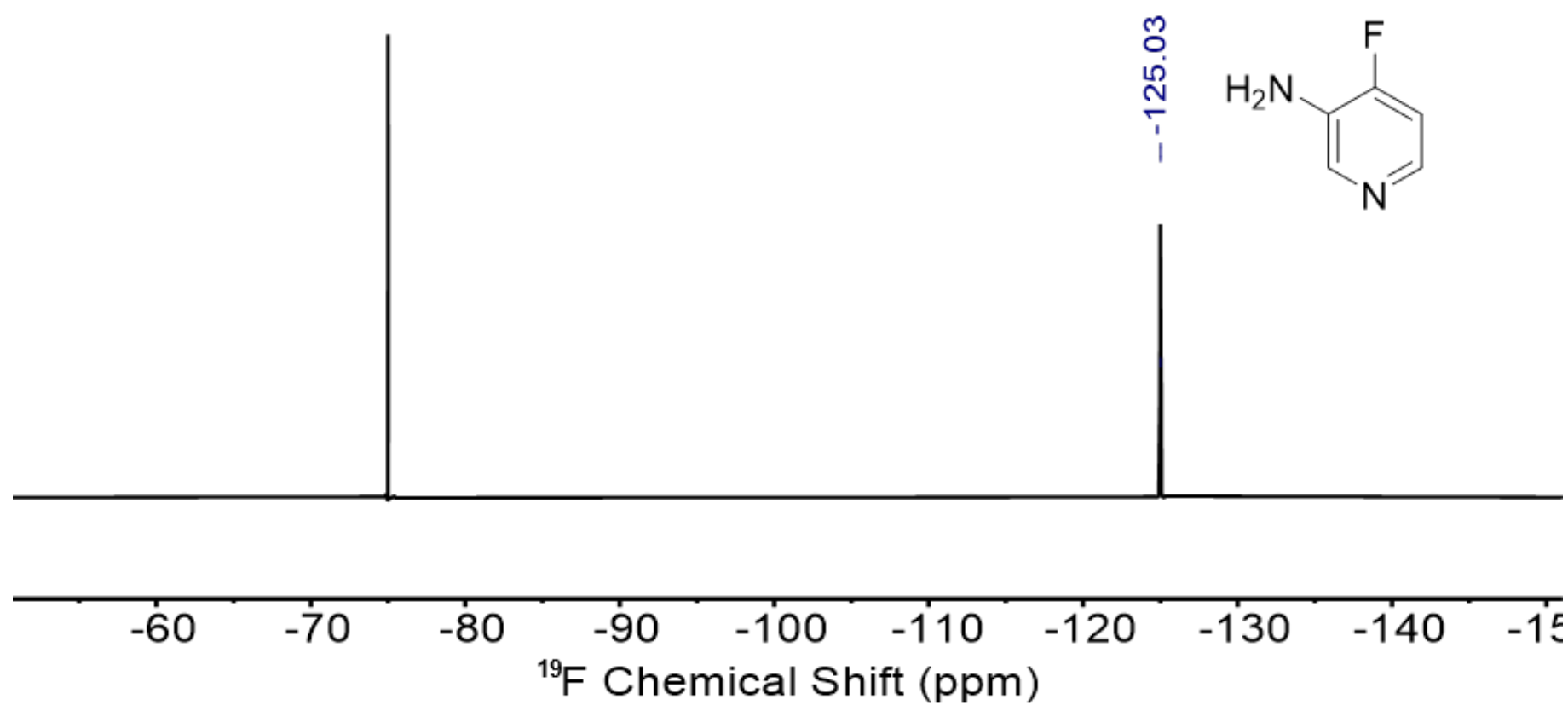

Fig. S34. ^{19}F NMR spectrum of substrate **f1**.

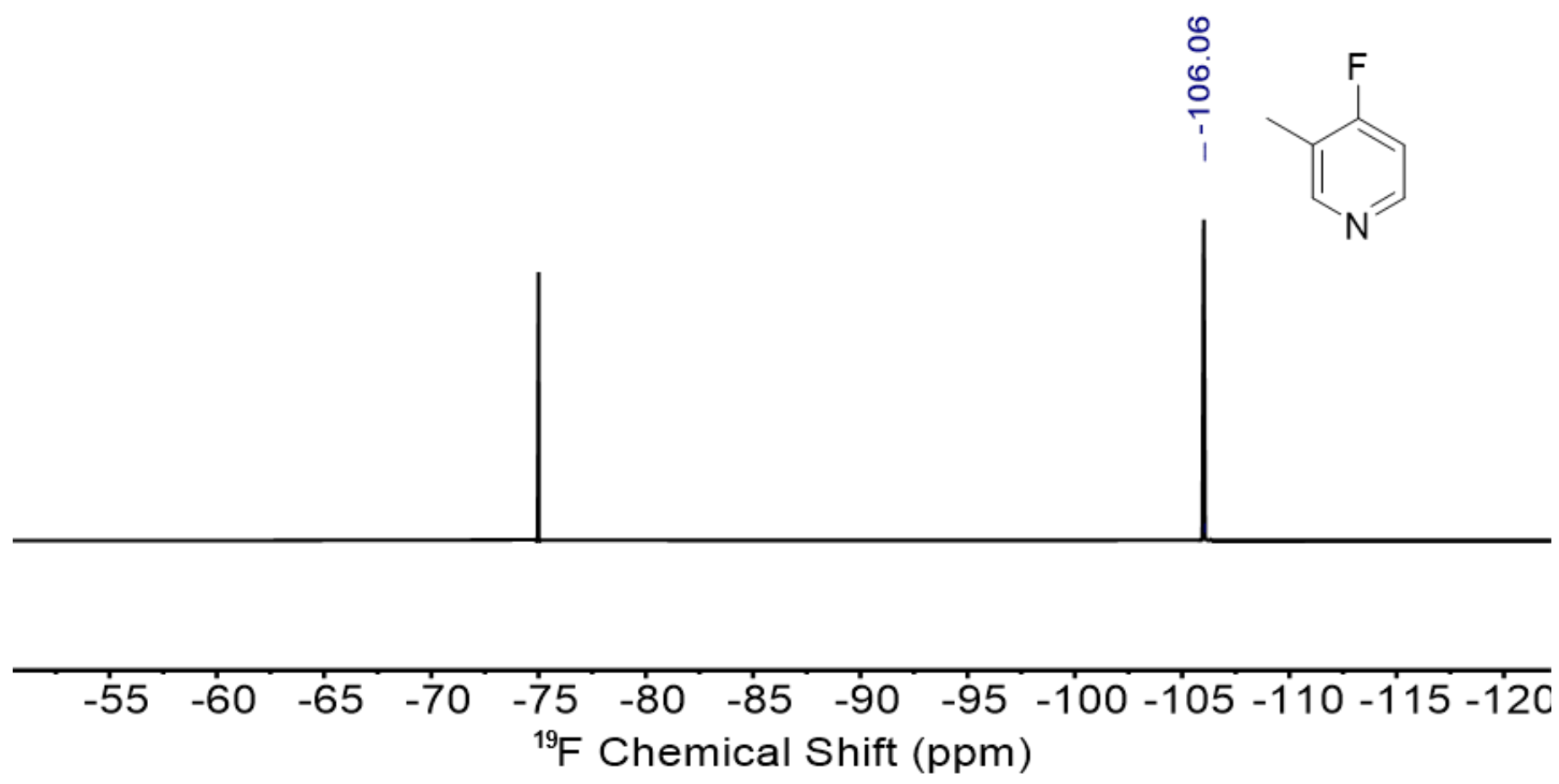

Fig. S35. ^{19}F NMR spectrum of substrate **f2**.

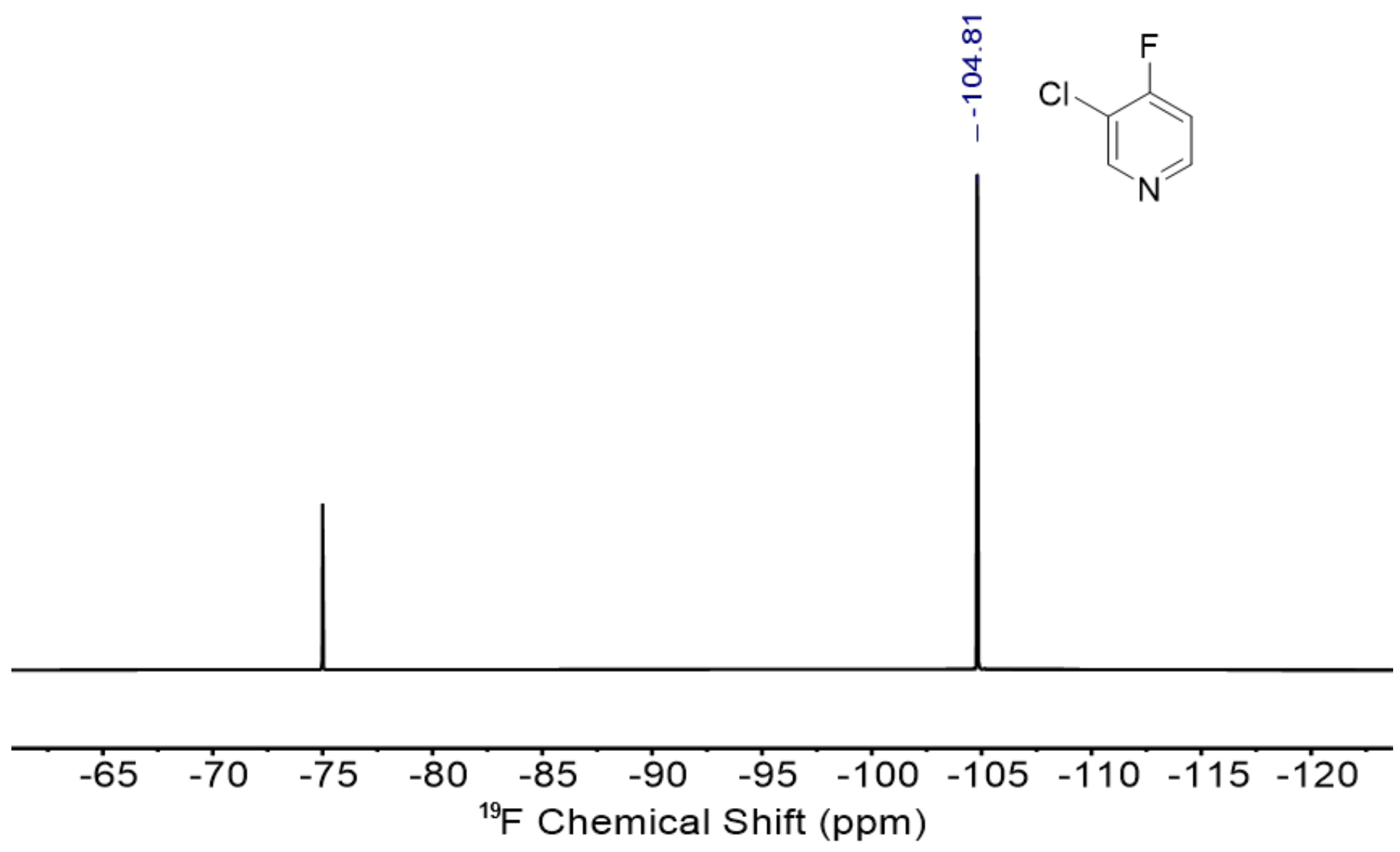

Fig. S36. ^{19}F NMR spectrum of substrate **f3**.

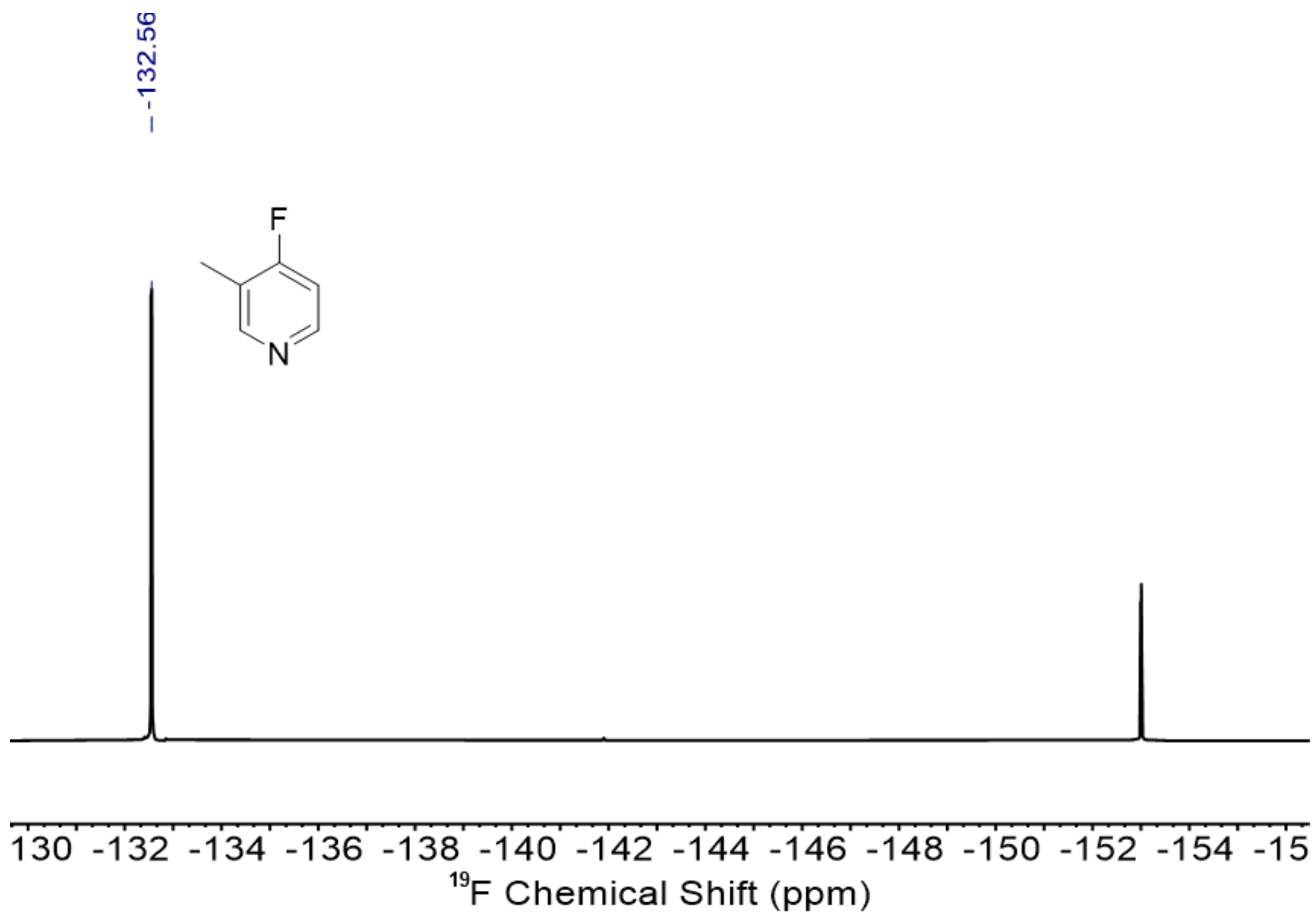

Fig. S37. ^{19}F NMR spectrum of substrate **f4**.

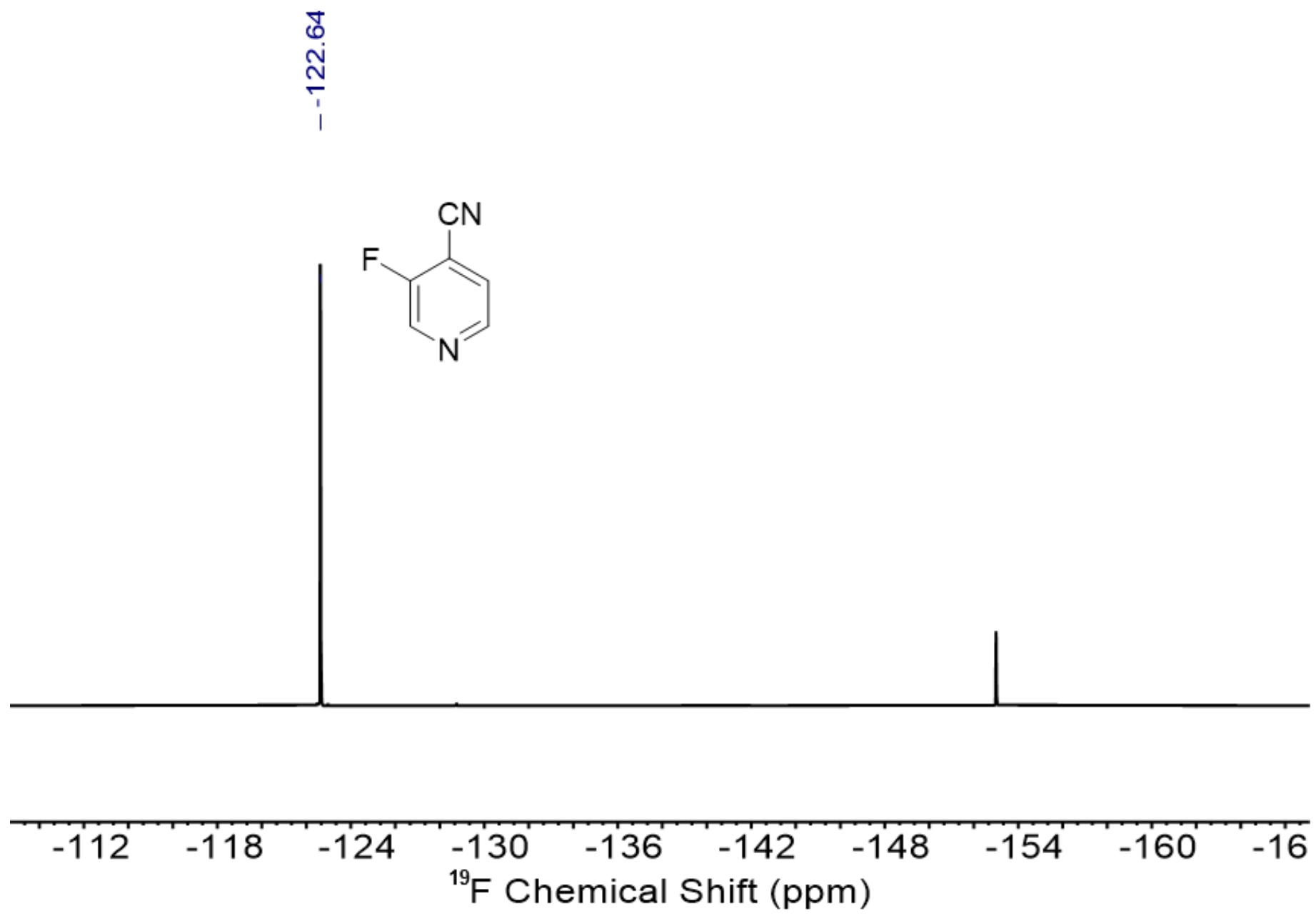

Fig. S38. ^{19}F NMR spectrum of substrate **f5**.

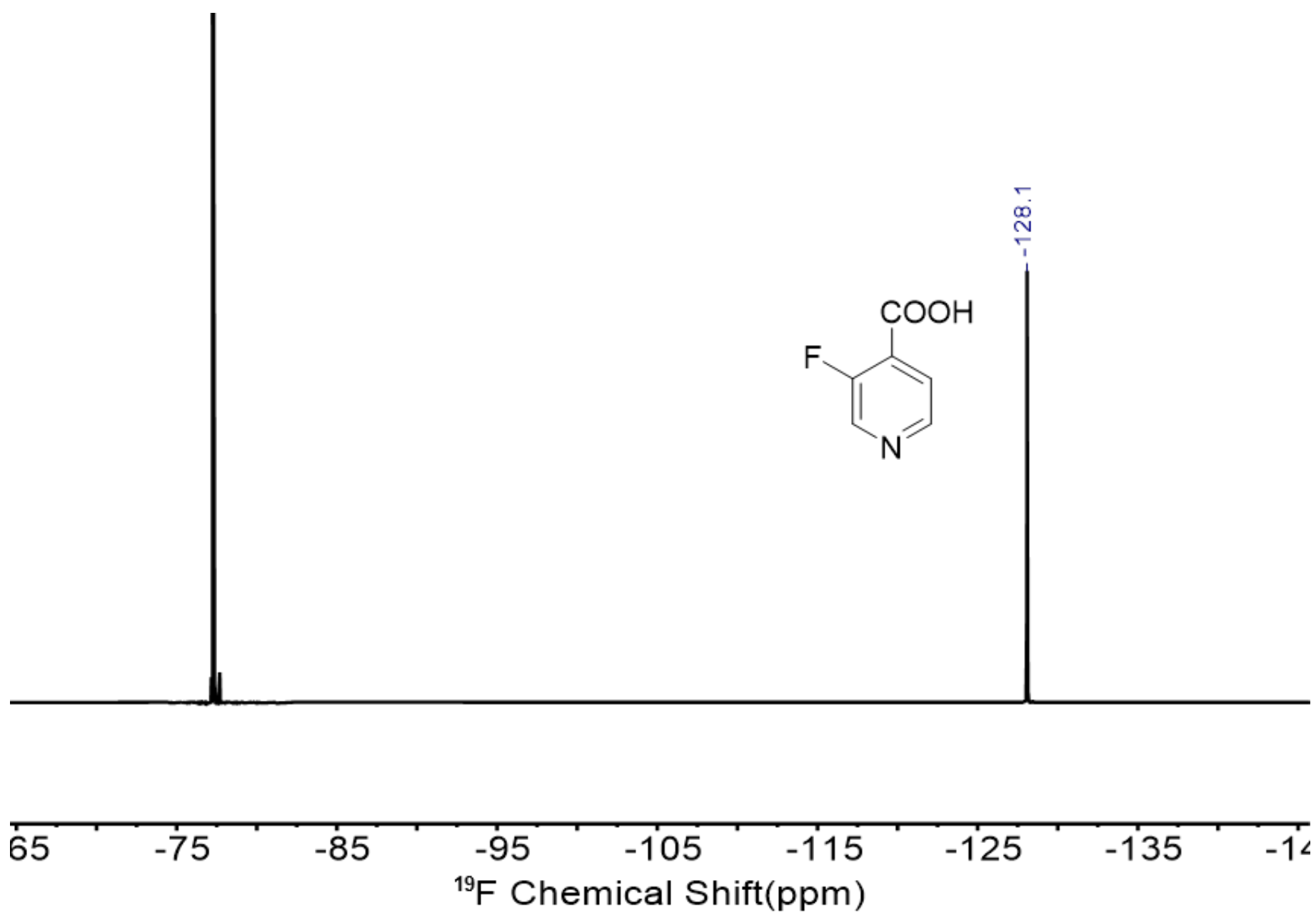

Fig. S39. ^{19}F NMR spectrum of substrate **f6**.

5 Single Substrate SABRE Hyperpolarized ^{19}F NMR Spectra

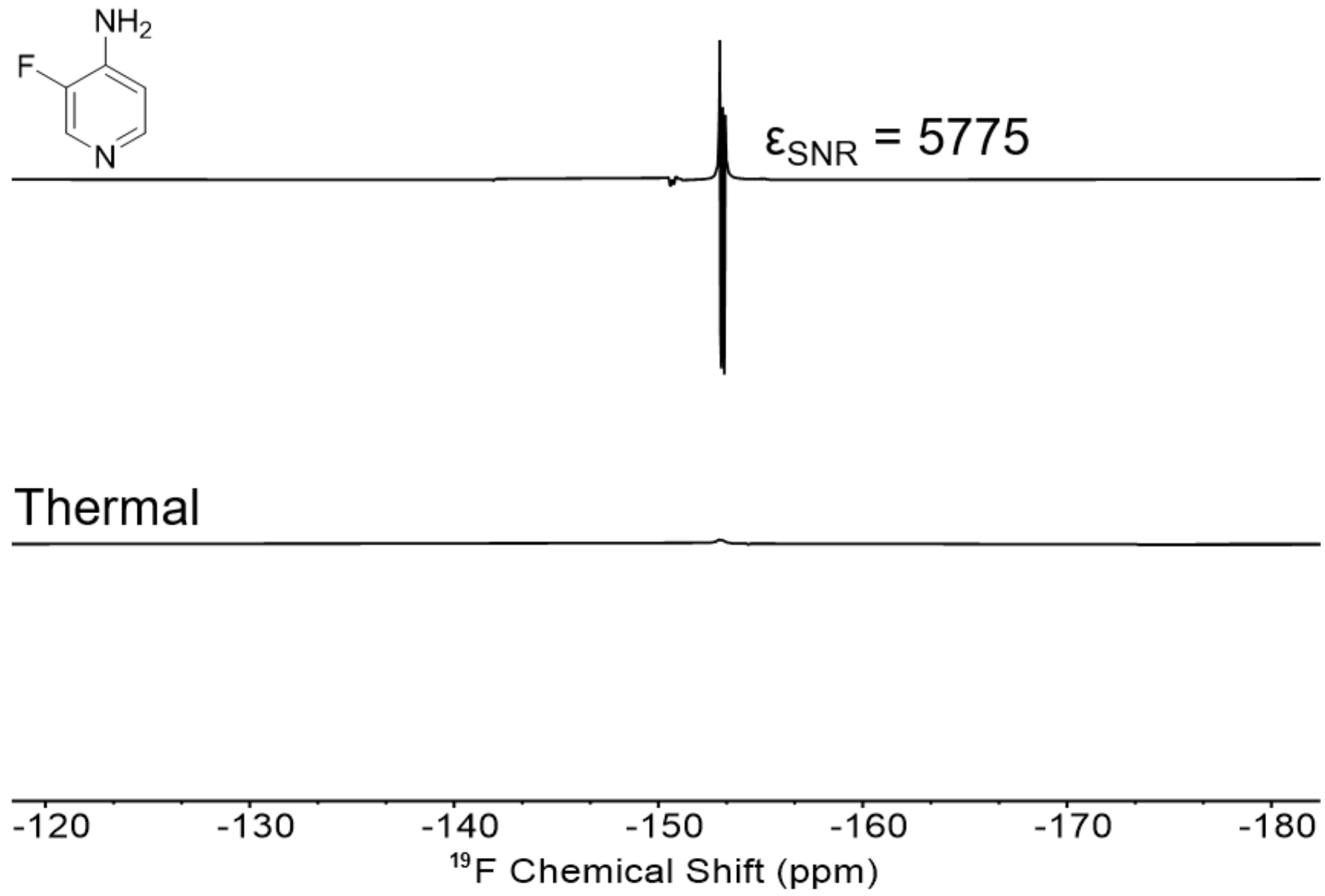

Fig. S40. ^{19}F NMR spectra of substrate **a1** at a concentration of 50 mM acquired under SABRE (top, 1 scan) and in the thermally polarized state (bottom, 64 scans).

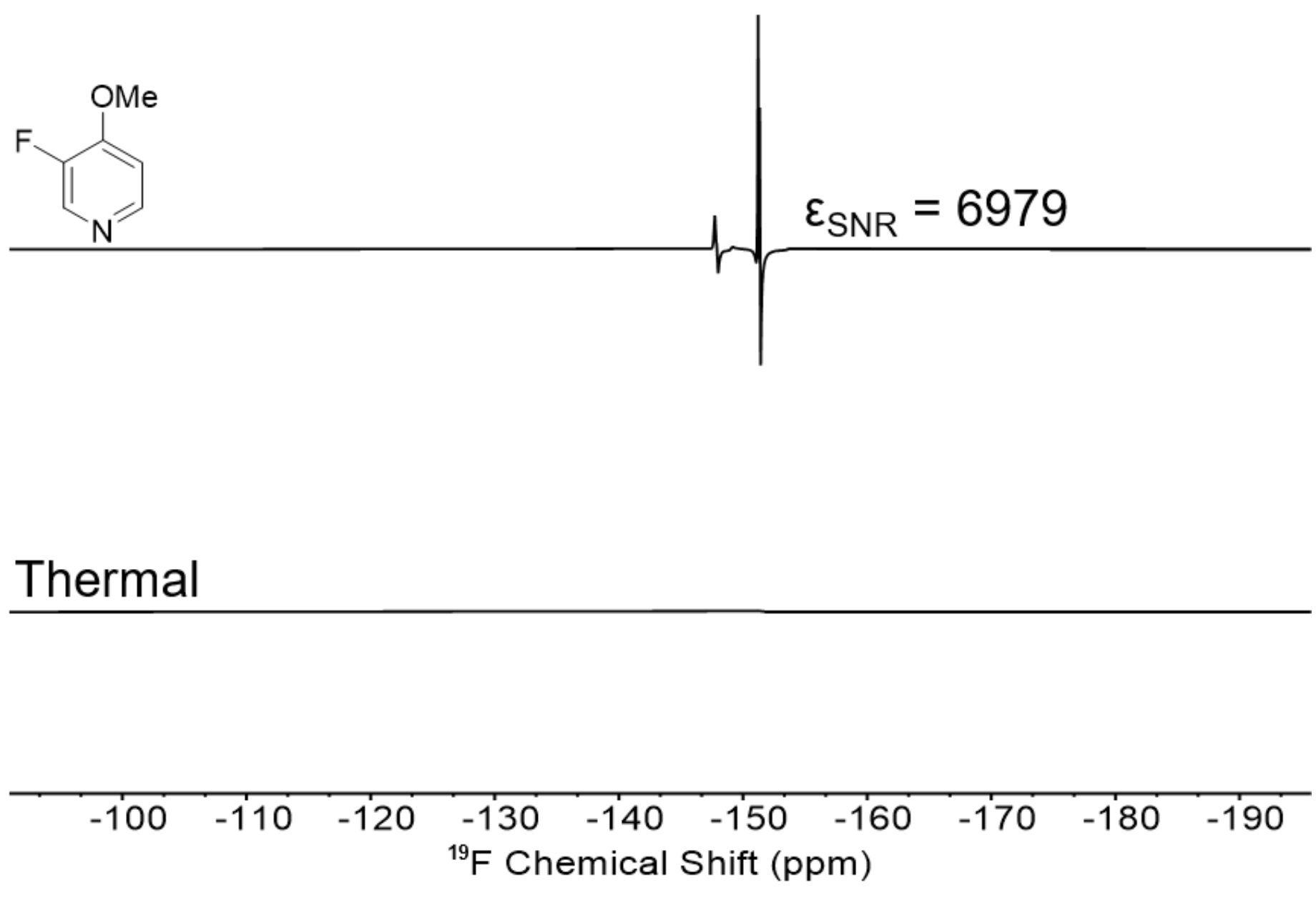

Fig. S41. ^{19}F NMR spectra of substrate **a2** at a concentration of 50 mM acquired under SABRE (top, 1 scan) and in the thermally polarized state (bottom, 64 scans).

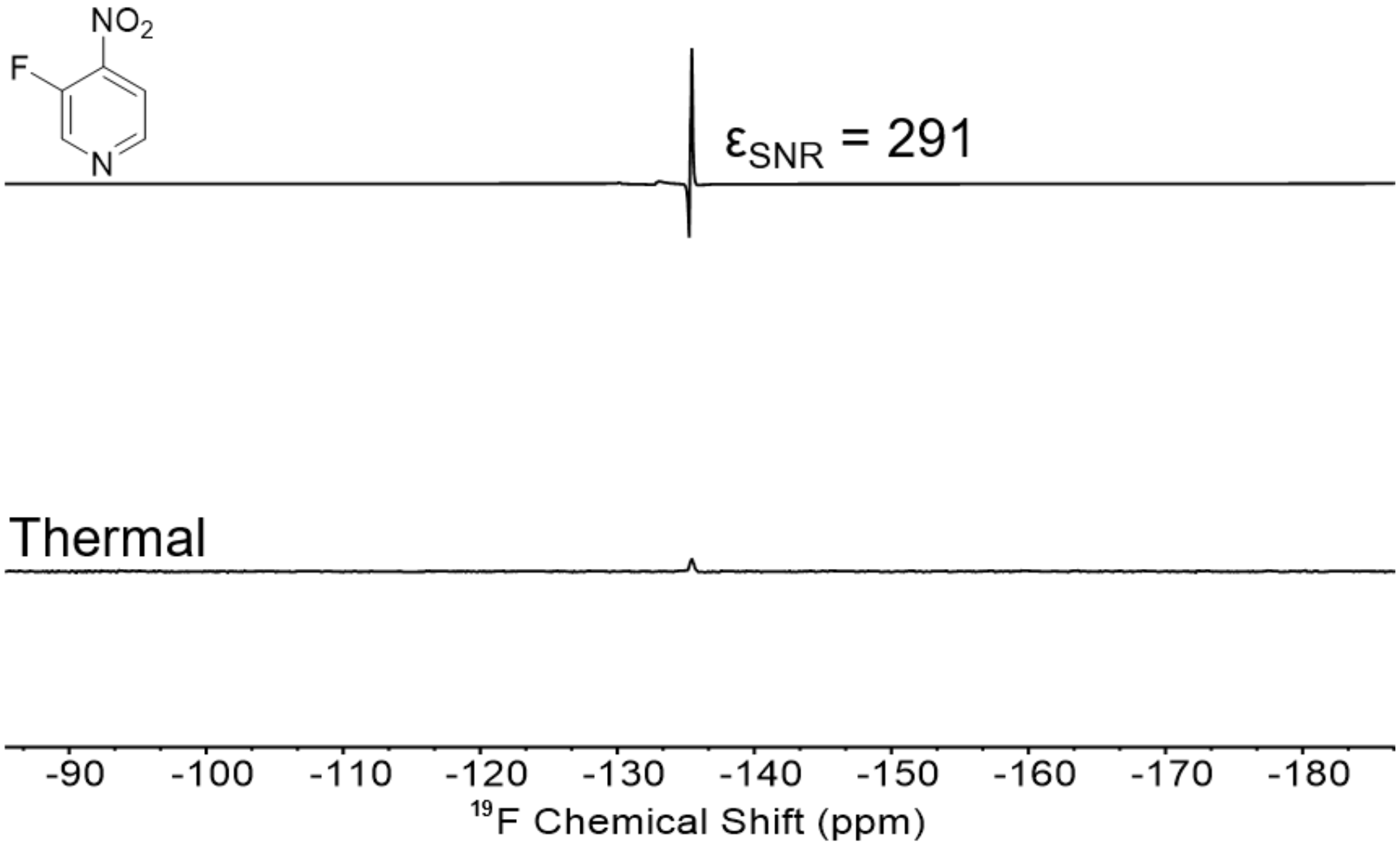

Fig. S42. ^{19}F NMR spectra of substrate **a3** at a concentration of 50 mM acquired under SABRE (top, 1 scan) and in the thermally polarized state (bottom, 64 scans).

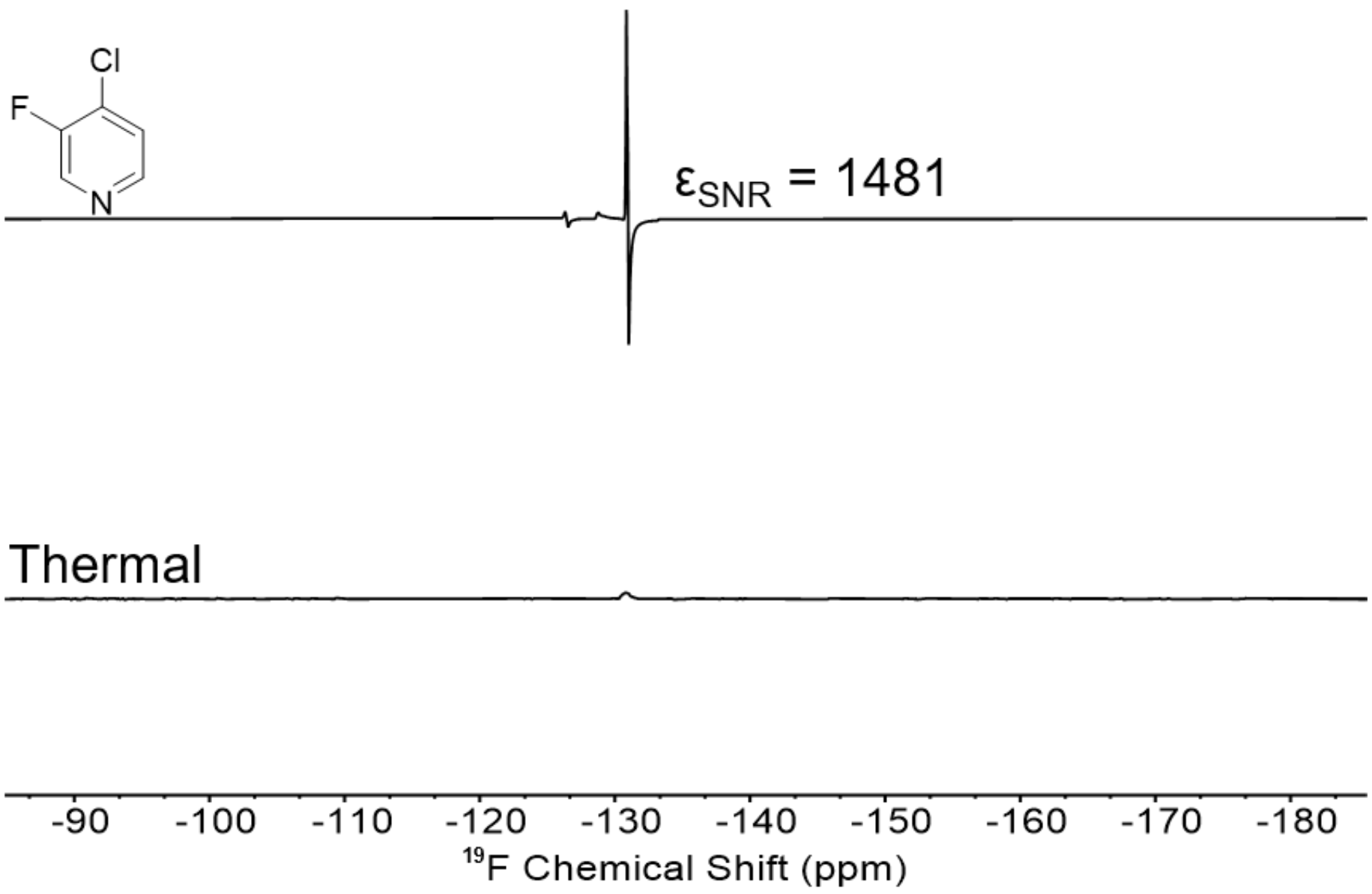

Fig. S43. ^{19}F NMR spectra of substrate **a4** at a concentration of 50 mM acquired under SABRE (top, 1 scan) and in the thermally polarized state (bottom, 64 scans).

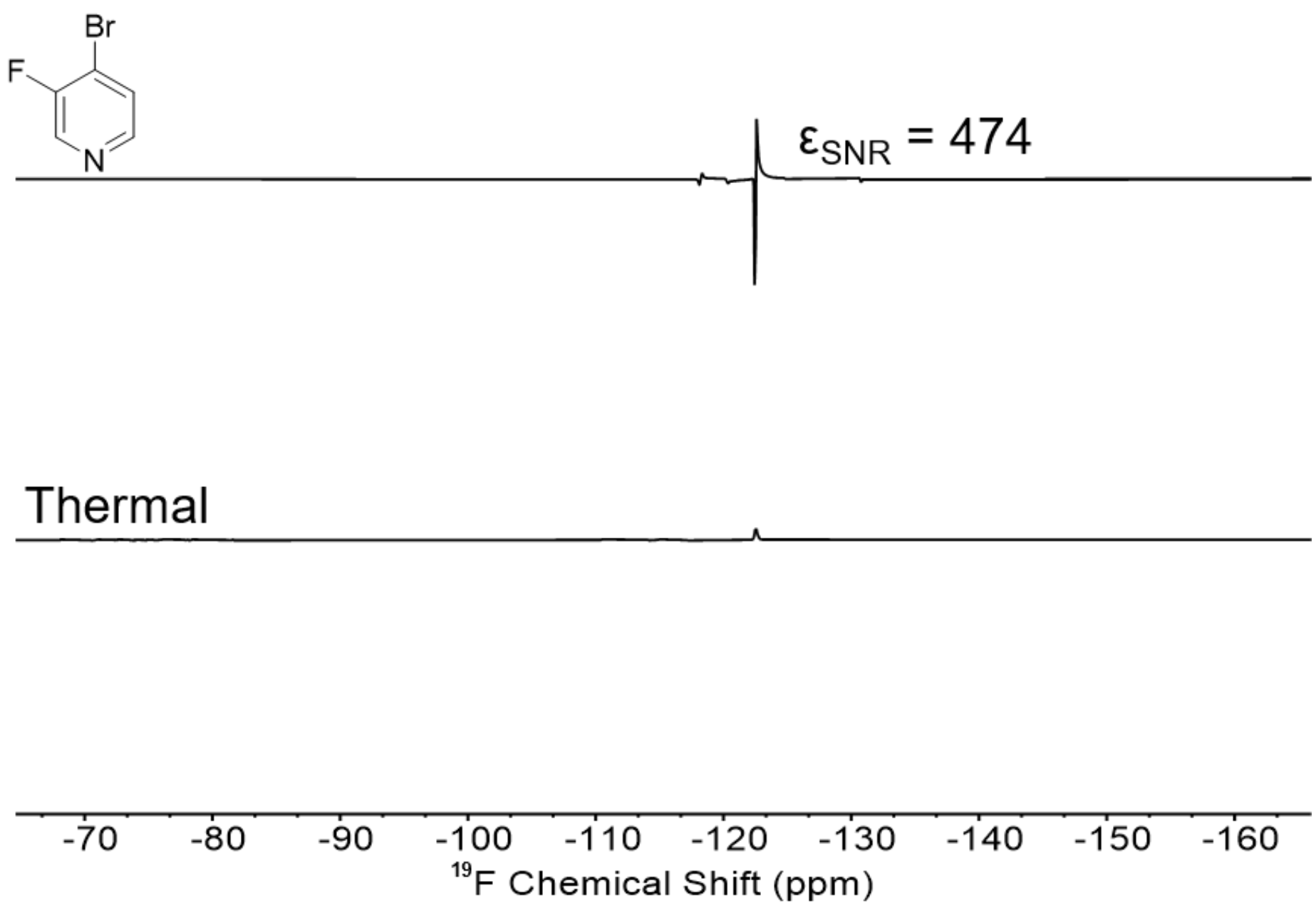

Fig. S44. ^{19}F NMR spectra of substrate **a5** at a concentration of 50 mM acquired under SABRE (top, 1 scan) and in the thermally polarized state (bottom, 64 scans).

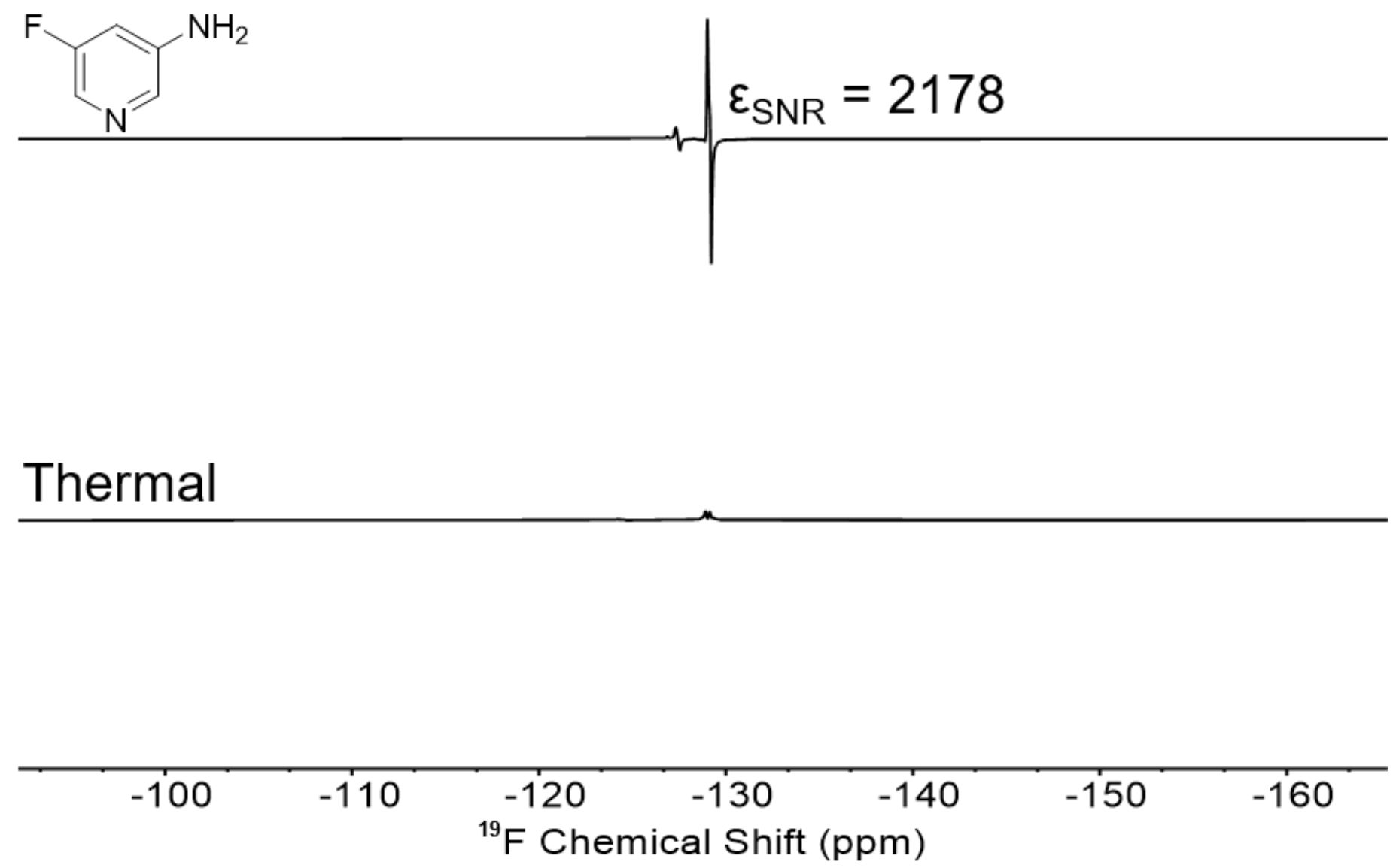

Fig. S45. ^{19}F NMR spectra of substrate **b1** at a concentration of 50 mM acquired under SABRE (top, 1 scan) and in the thermally polarized state (bottom, 64 scans).

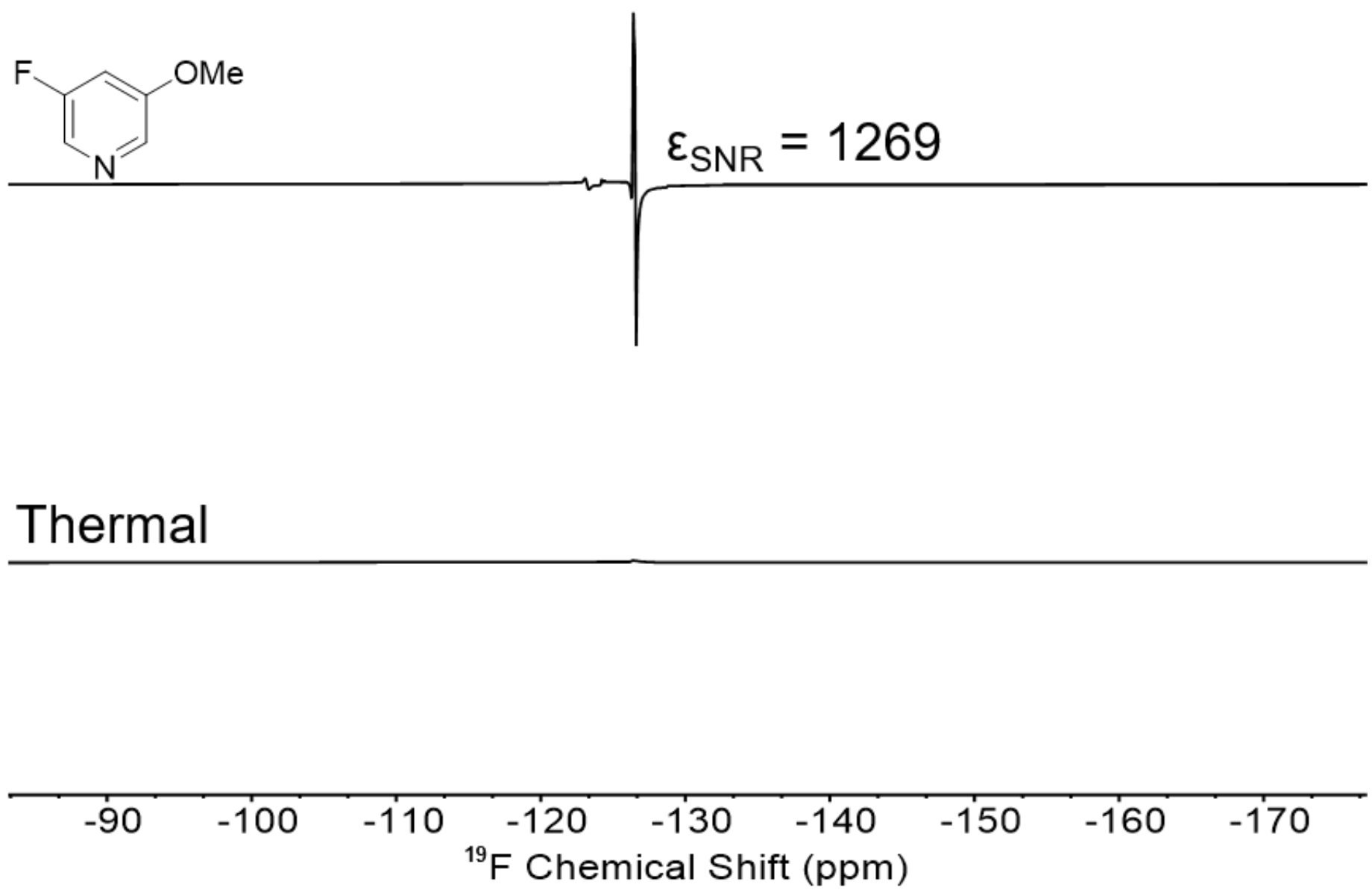

Fig. S46. ^{19}F NMR spectra of substrate **b2** at a concentration of 50 mM acquired under SABRE (top, 1 scan) and in the thermally polarized state (bottom, 64 scans).

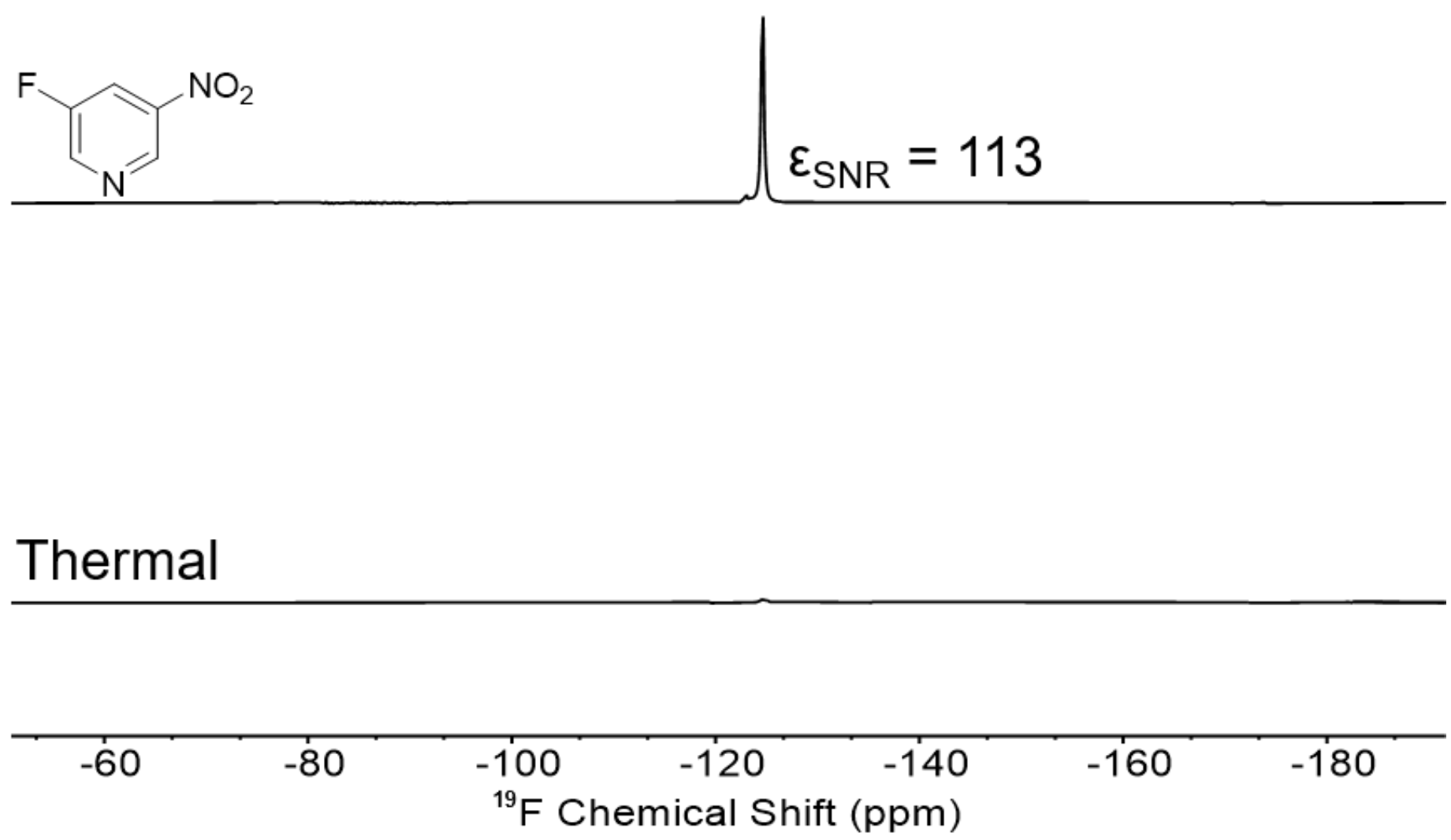

Fig. S47. ^{19}F NMR spectra of substrate **b3** at a concentration of 50 mM acquired under SABRE (top, 1 scan) and in the thermally polarized state (bottom, 64 scans).

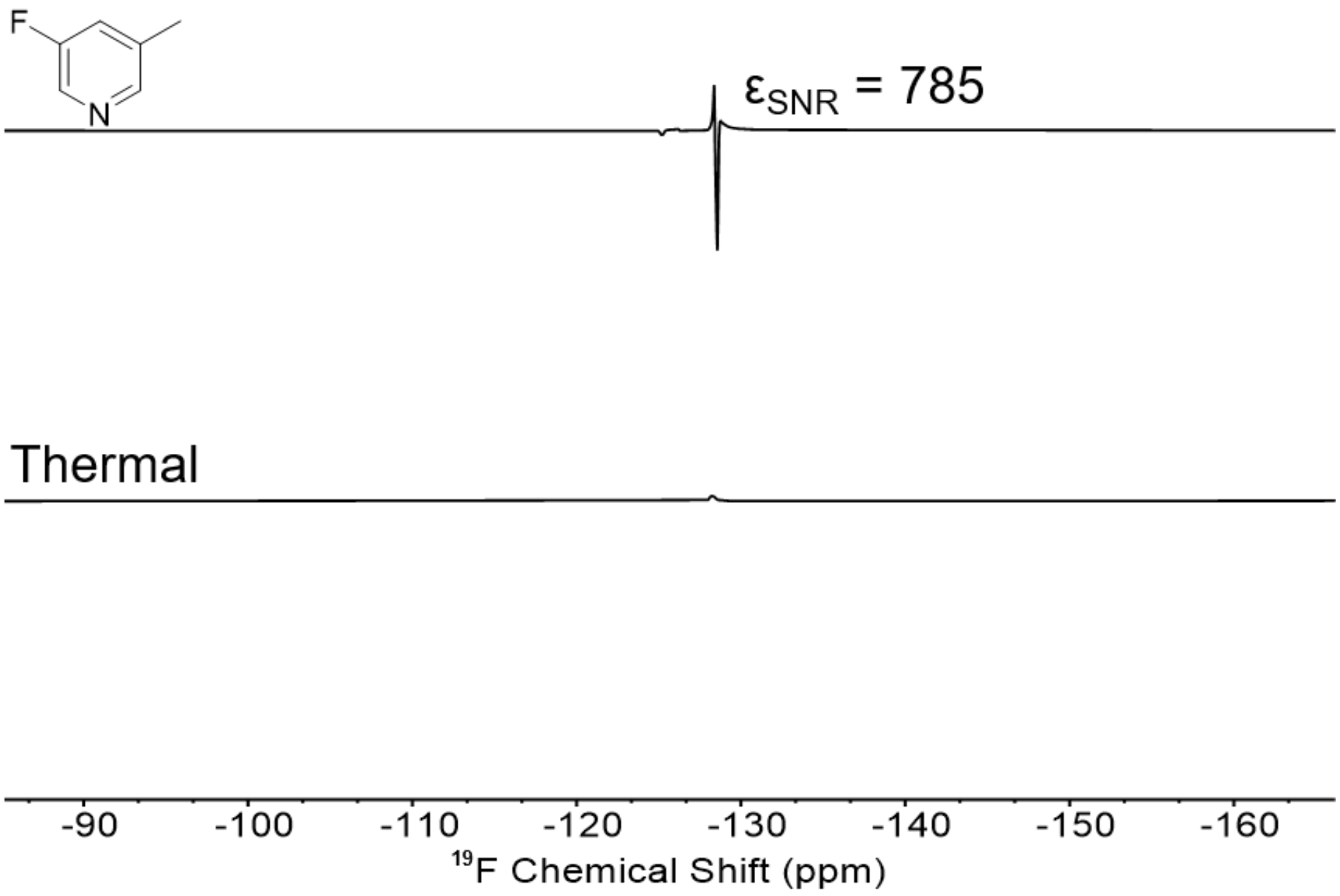

Fig. S48. ^{19}F NMR spectra of substrate **b4** at a concentration of 50 mM acquired under SABRE (top, 1 scan) and in the thermally polarized state (bottom, 64 scans).

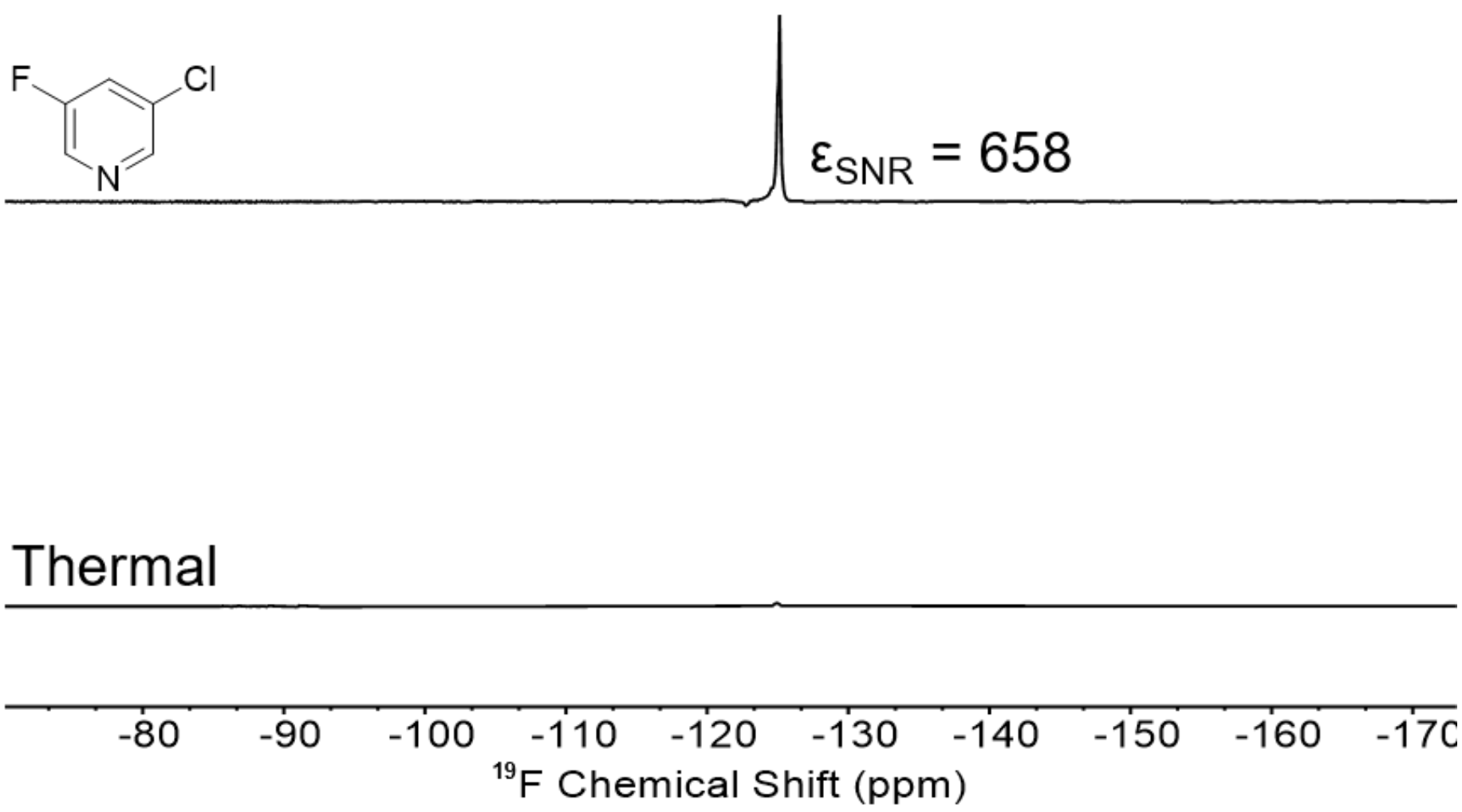

Fig. S49. ^{19}F NMR spectra of substrate **b5** at a concentration of 50 mM acquired under SABRE (top, 1 scan) and in the thermally polarized state (bottom, 64 scans).

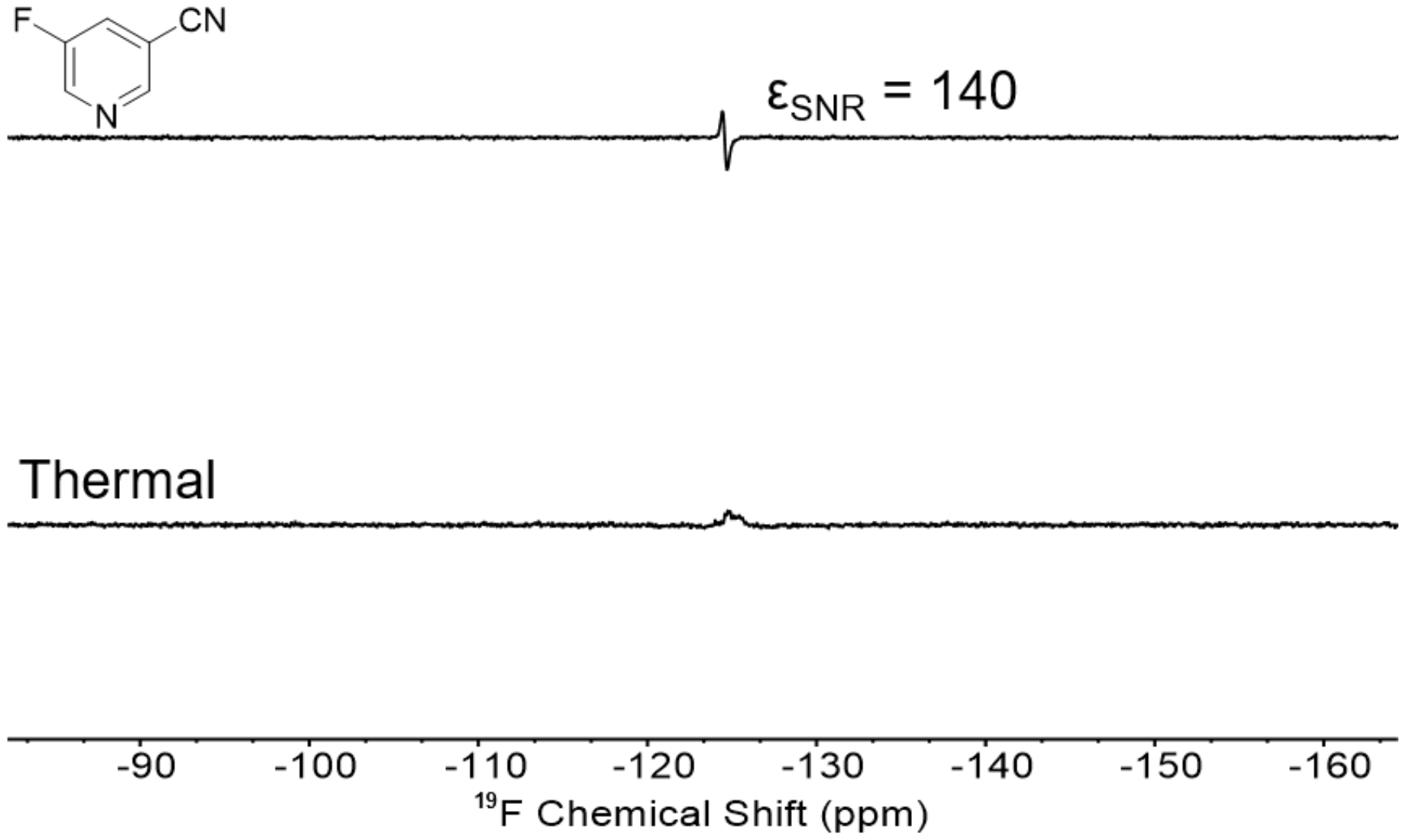

Fig. S50. ^{19}F NMR spectra of substrate **b6** at a concentration of 50 mM acquired under SABRE (top, 1 scan) and in the thermally polarized state (bottom, 64 scans).

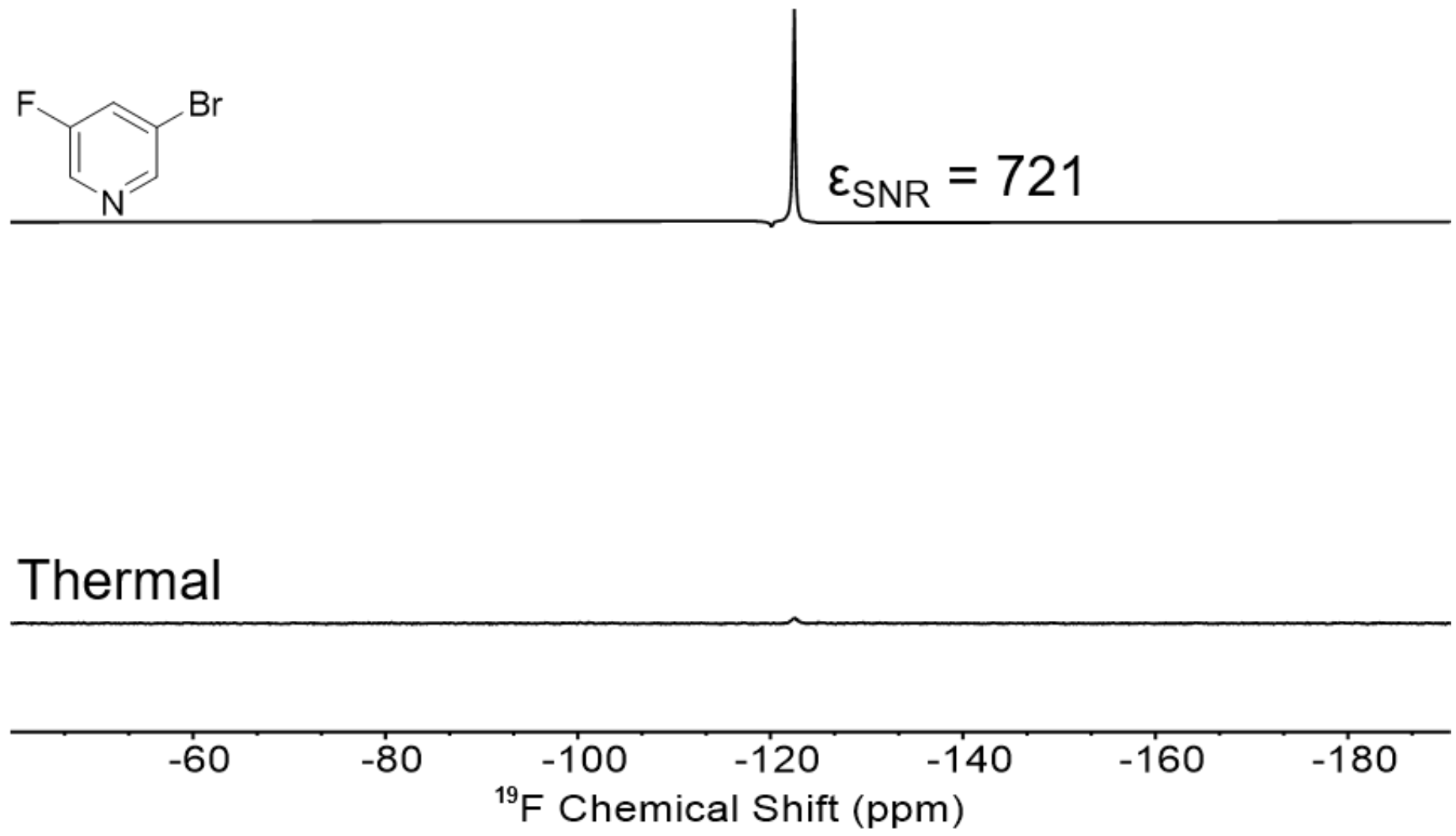

Fig. S51. ^{19}F NMR spectra of substrate **b7** at a concentration of 50 mM acquired under SABRE (top, 1 scan) and in the thermally polarized state (bottom, 64 scans).

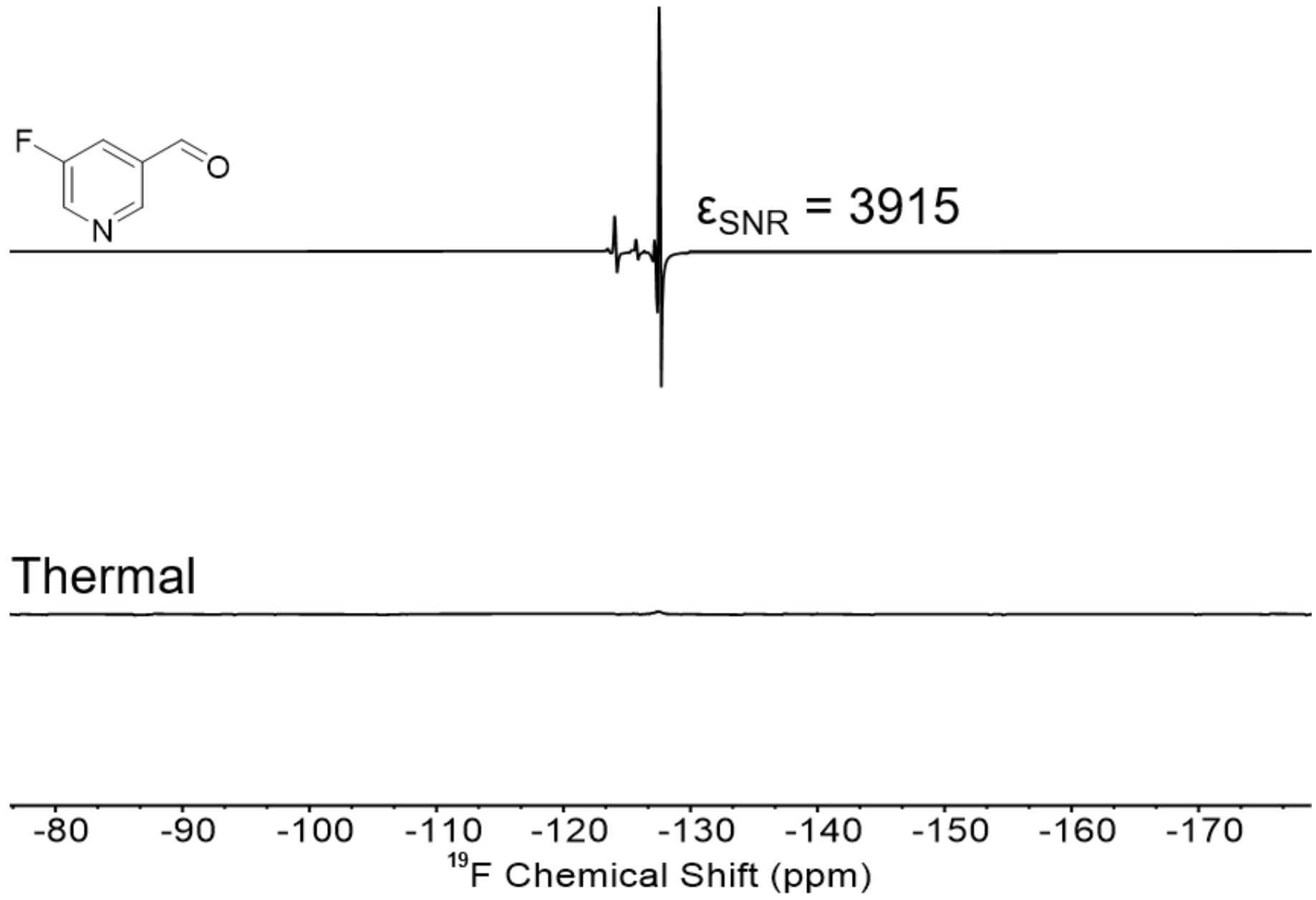

Fig. S52. ^{19}F NMR spectra of substrate **b8** at a concentration of 50 mM acquired under SABRE (top, 1 scan) and in the thermally polarized state (bottom, 64 scans).

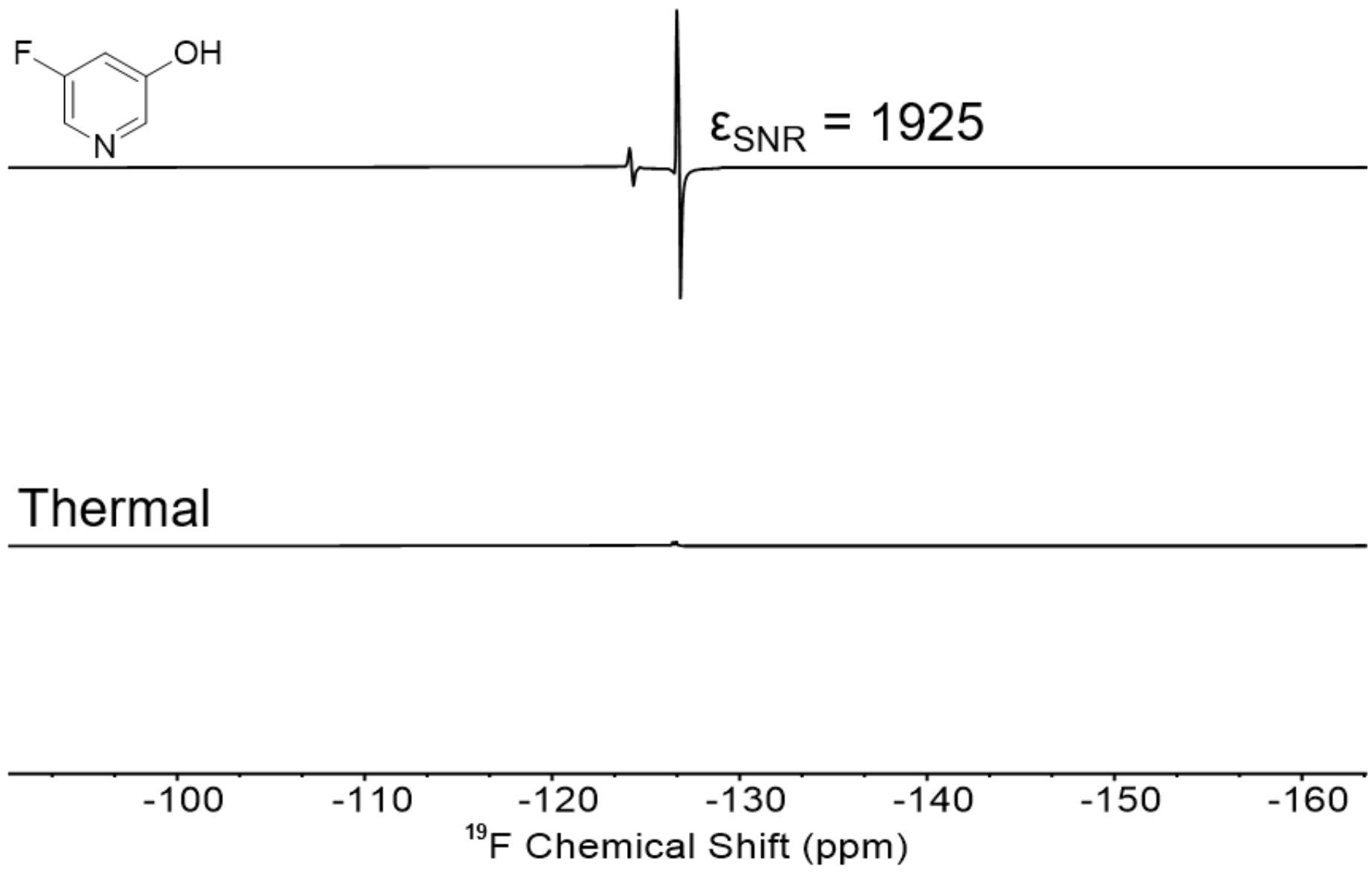

Fig. S53. ^{19}F NMR spectra of substrate **b9** at a concentration of 50 mM acquired under SABRE (top, 1 scan) and in the thermally polarized state (bottom, 64 scans).

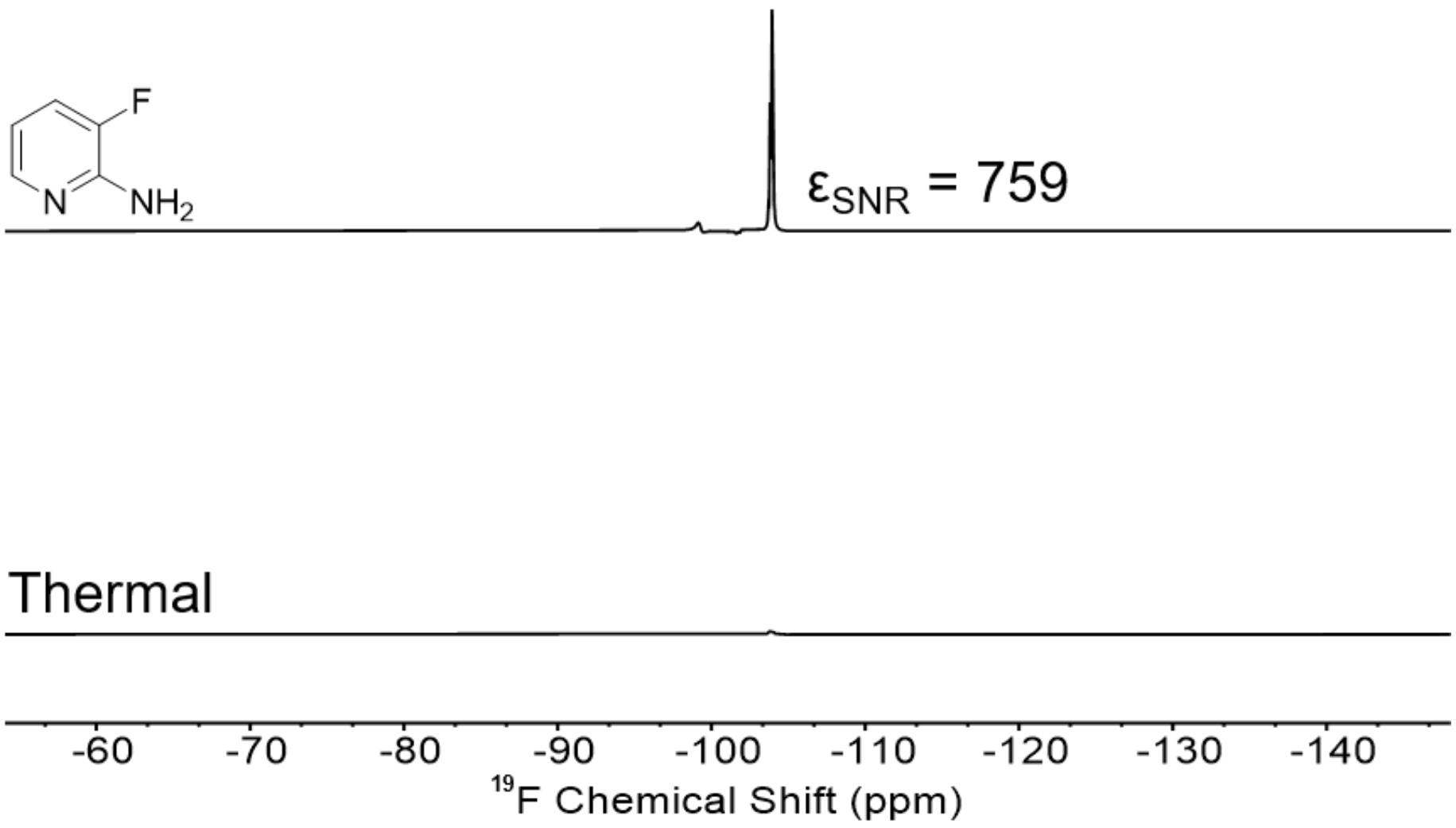

Fig. S54. ^{19}F NMR spectra of substrate **c1** at a concentration of 50 mM acquired under SABRE (top, 1 scan) and in the thermally polarized state (bottom, 64 scans).

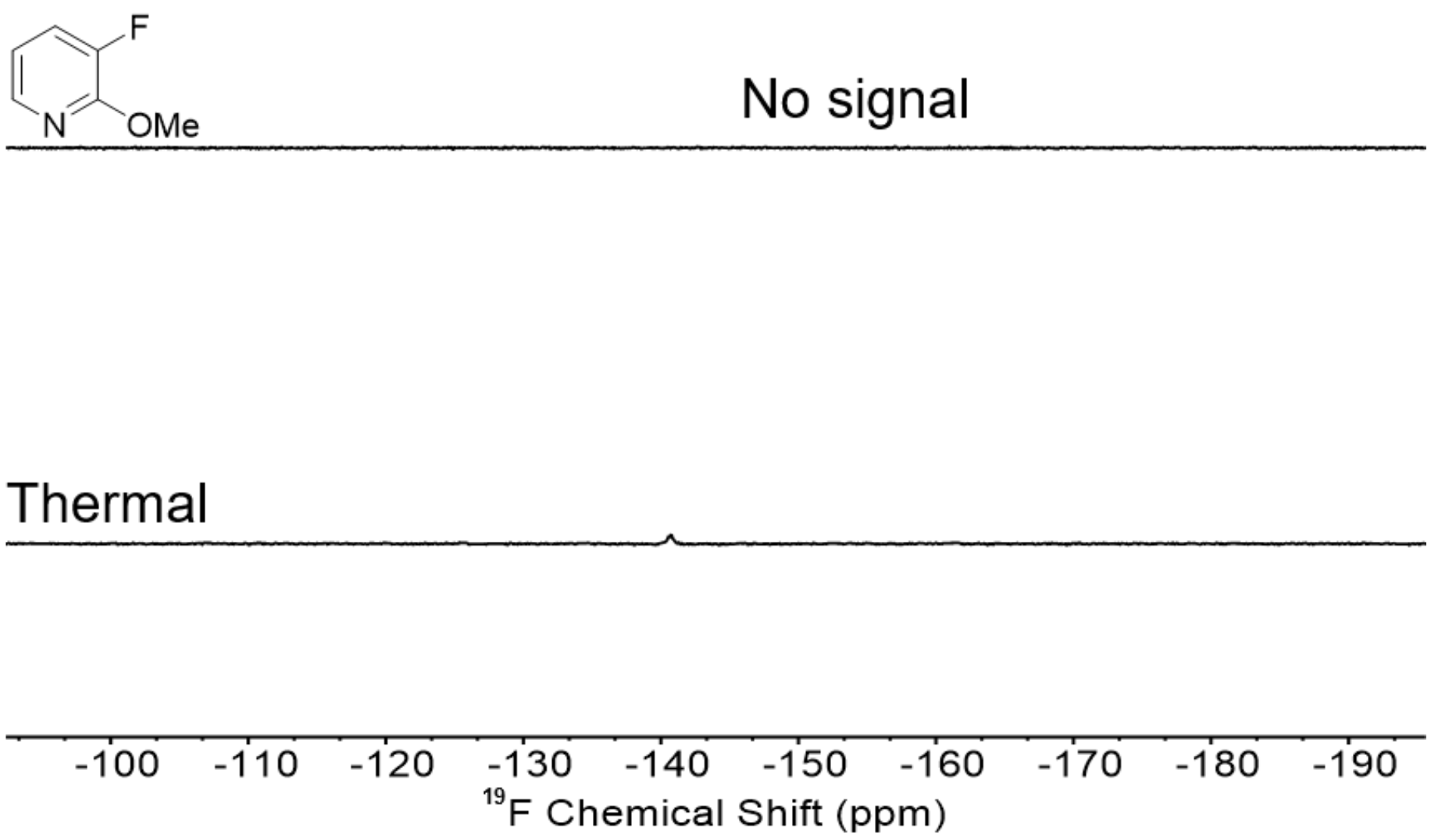

Fig. S55. ^{19}F NMR spectra of substrate **c2** at a concentration of 50 mM acquired under SABRE (top, 1 scan) and in the thermally polarized state (bottom, 64 scans).

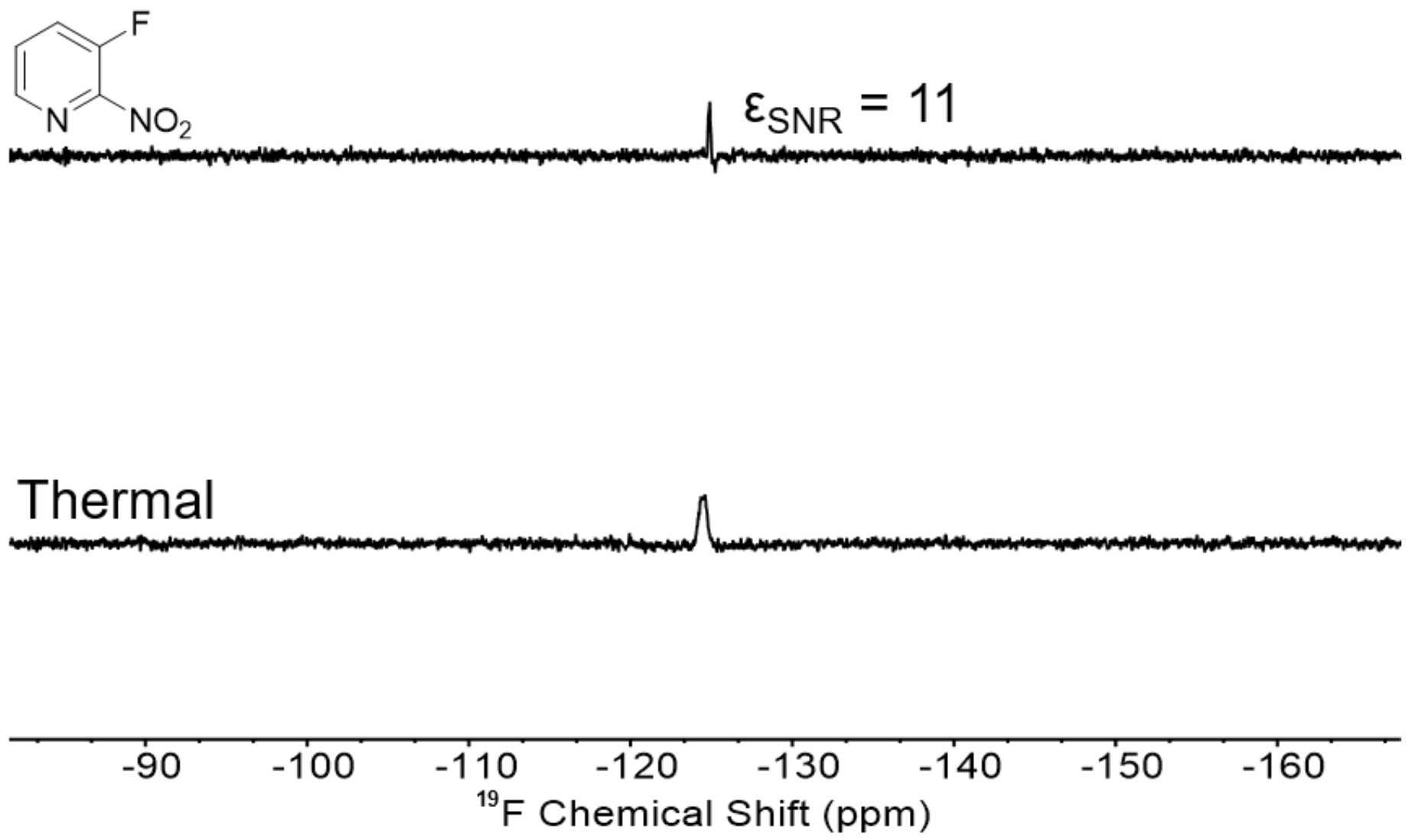

Fig. S56. ^{19}F NMR spectra of substrate **c3** at a concentration of 50 mM acquired under SABRE (top, 1 scan) and in the thermally polarized state (bottom, 64 scans).

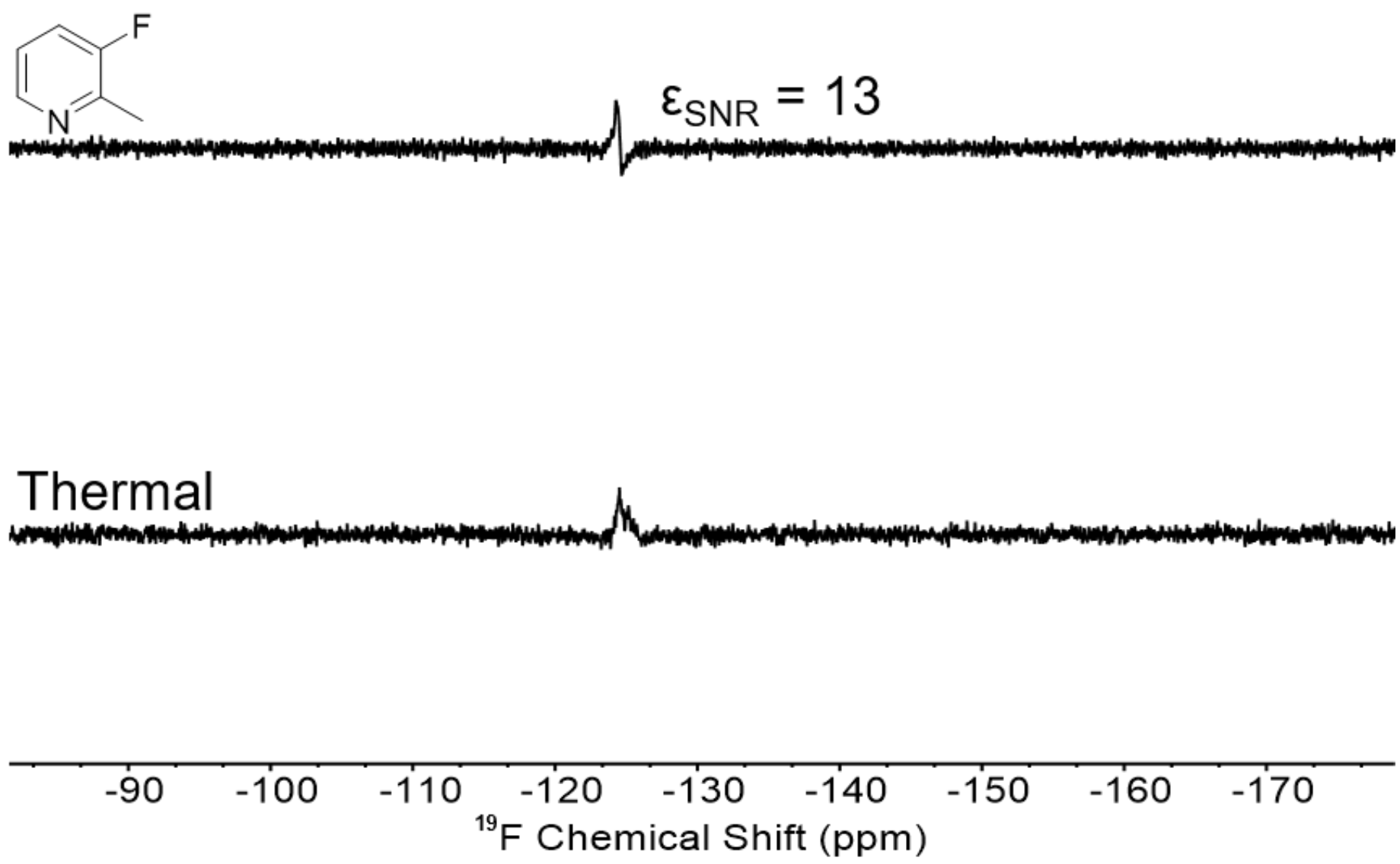

Fig. S57. ^{19}F NMR spectra of substrate **c4** at a concentration of 50 mM acquired under SABRE (top, 1 scan) and in the thermally polarized state (bottom, 64 scans).

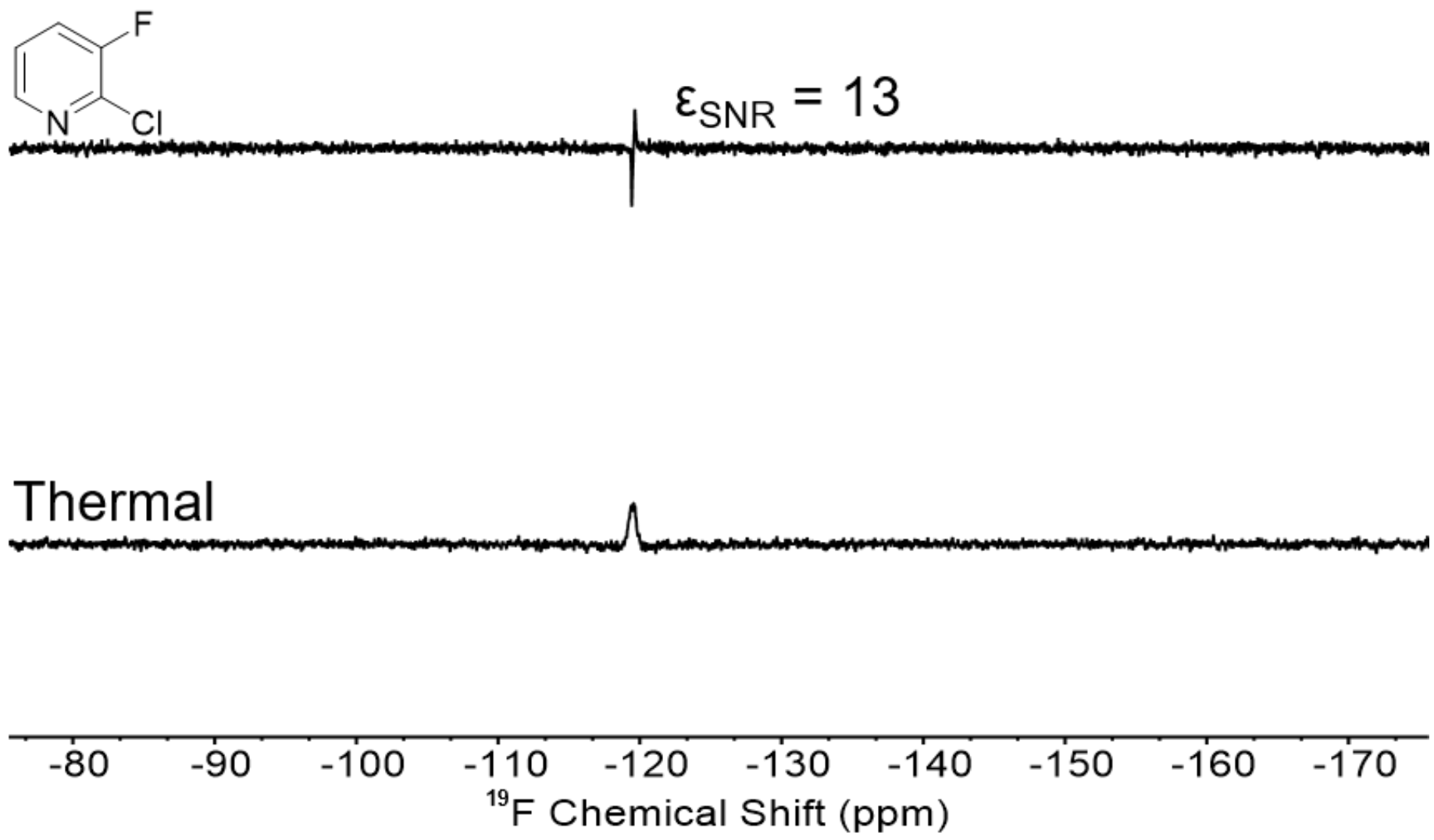

Fig. S58. ^{19}F NMR spectra of substrate **c5** at a concentration of 50 mM acquired under SABRE (top, 1 scan) and in the thermally polarized state (bottom, 64 scans).

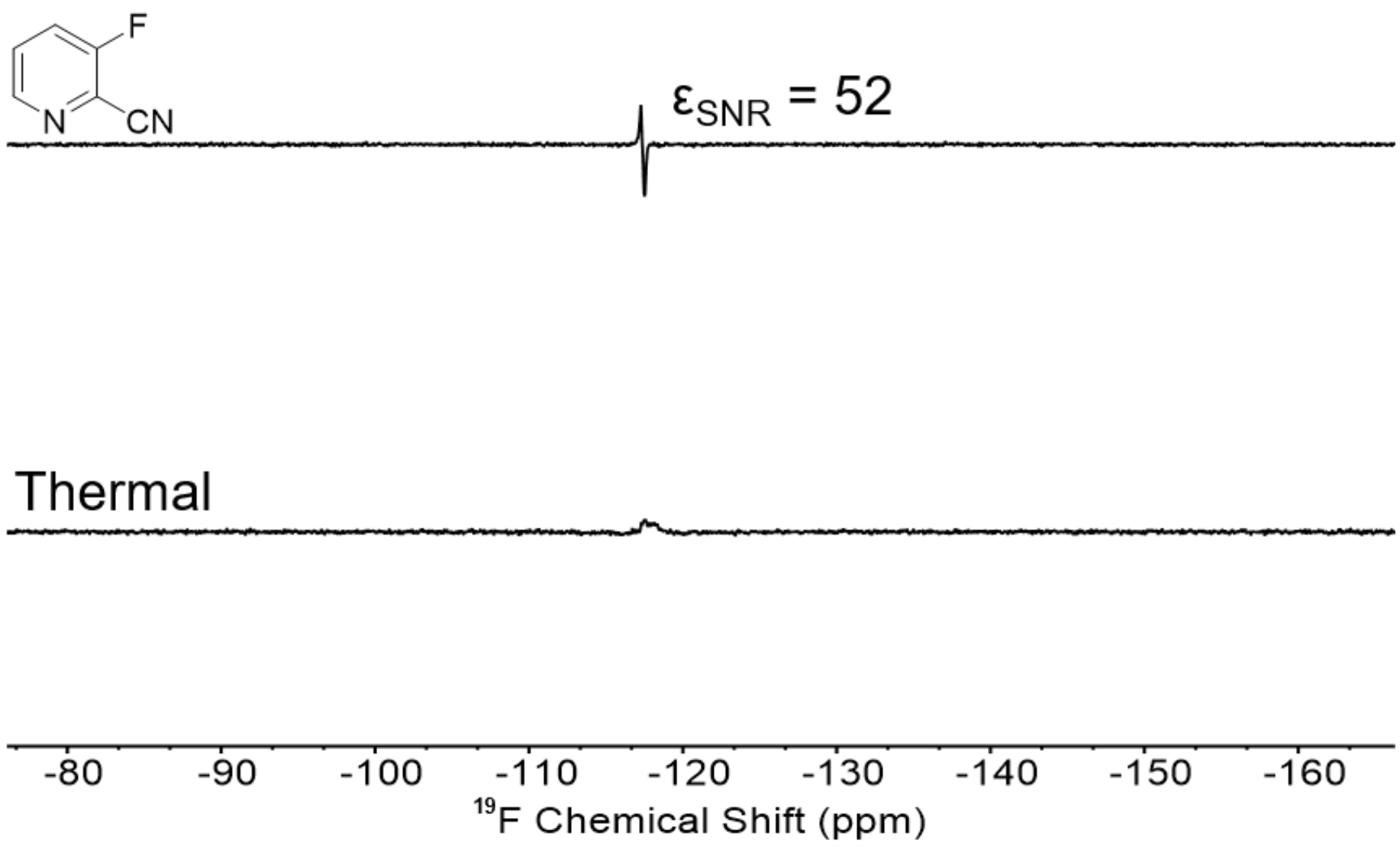

Fig. S59. ^{19}F NMR spectra of substrate **c6** at a concentration of 50 mM acquired under SABRE (top, 1 scan) and in the thermally polarized state (bottom, 64 scans).

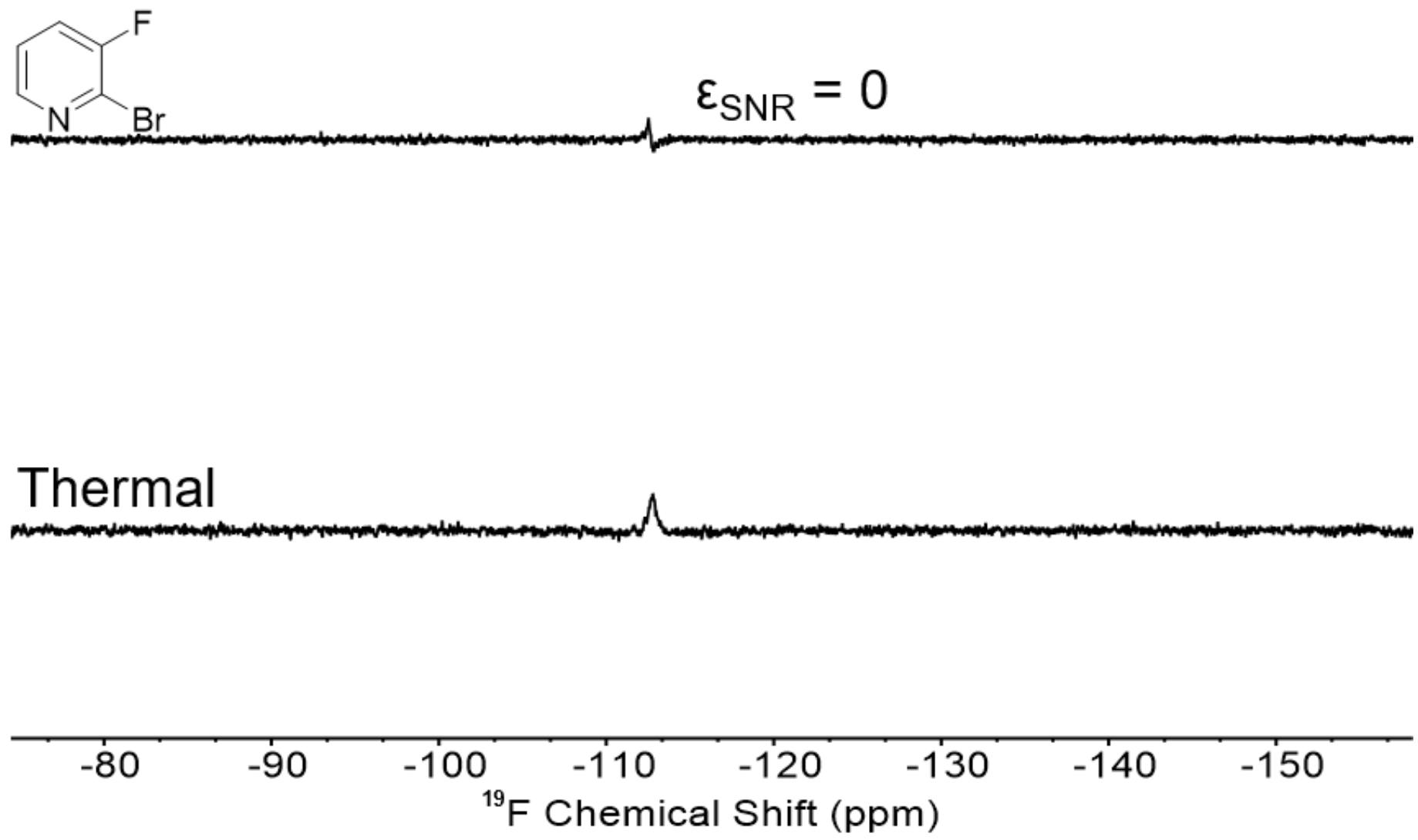

Fig. S60. ^{19}F NMR spectra of substrate **c7** at a concentration of 50 mM acquired under SABRE (top, 1 scan) and in the thermally polarized state (bottom, 64 scans).

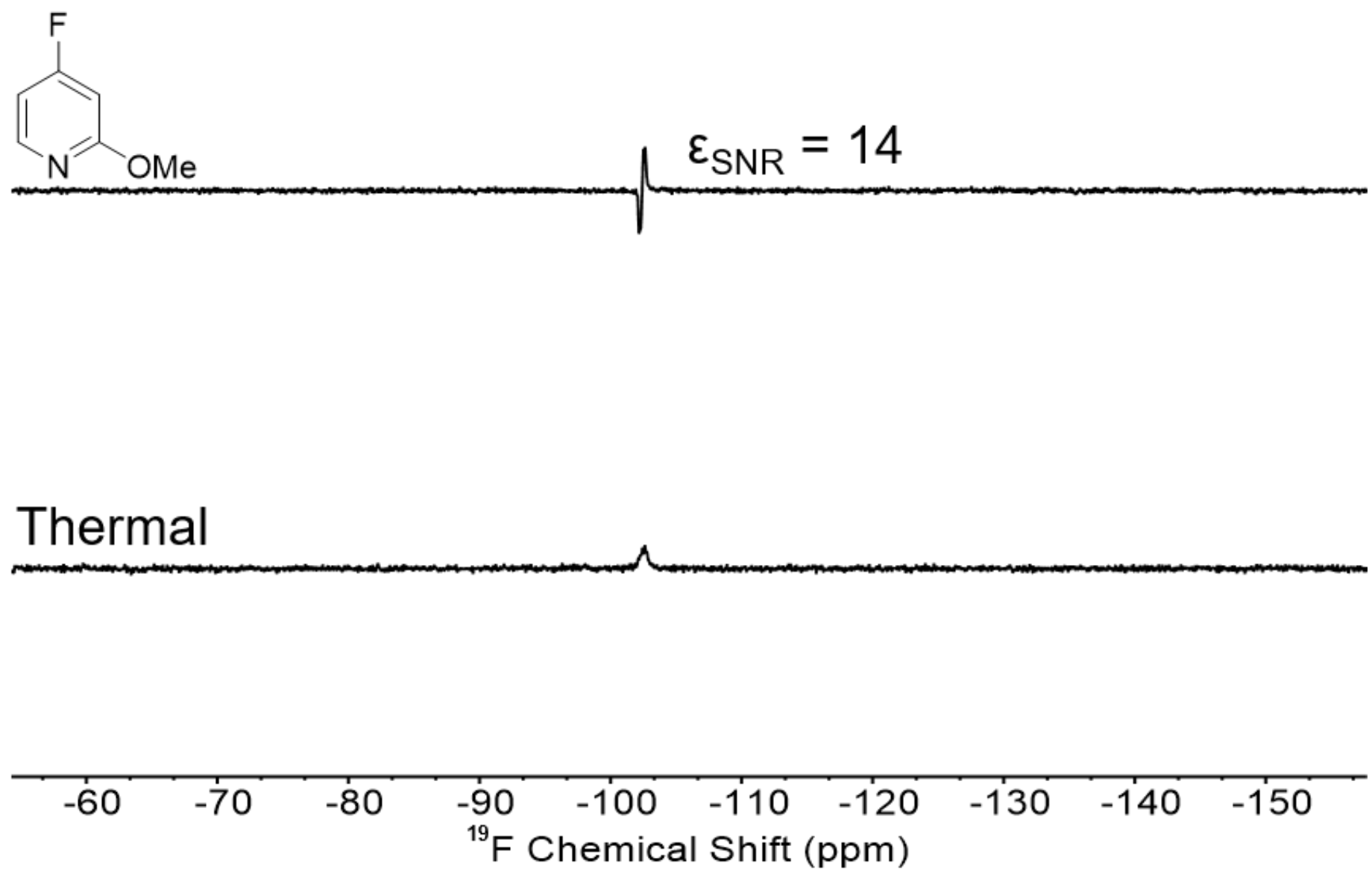

Fig. S61. ^{19}F NMR spectra of substrate **d1** at a concentration of 50 mM acquired under SABRE (top, 1 scan) and in the thermally polarized state (bottom, 64 scans).

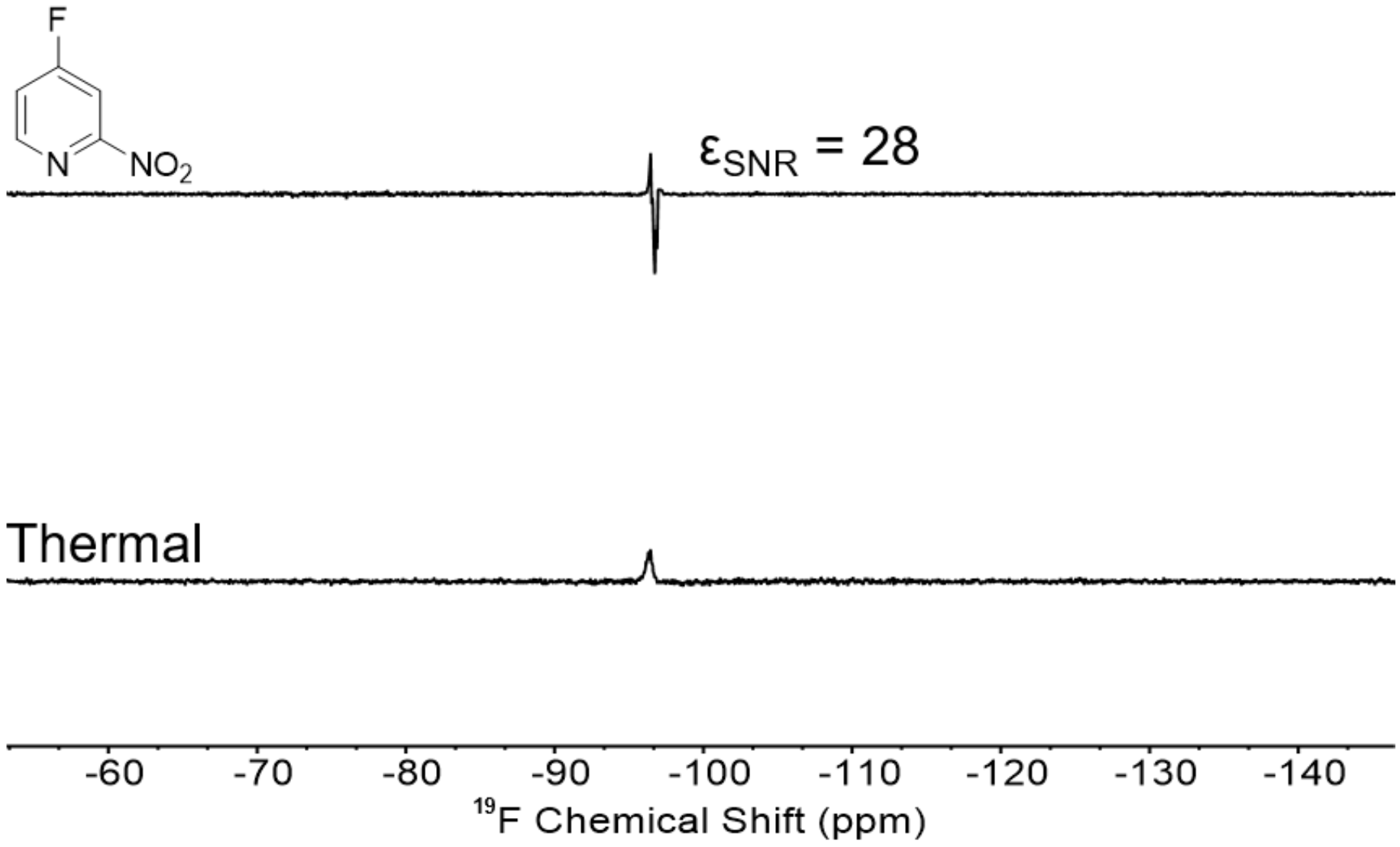

Fig. S62. ^{19}F NMR spectra of substrate **d2** at a concentration of 50 mM acquired under SABRE (top, 1 scan) and in the thermally polarized state (bottom, 64 scans).

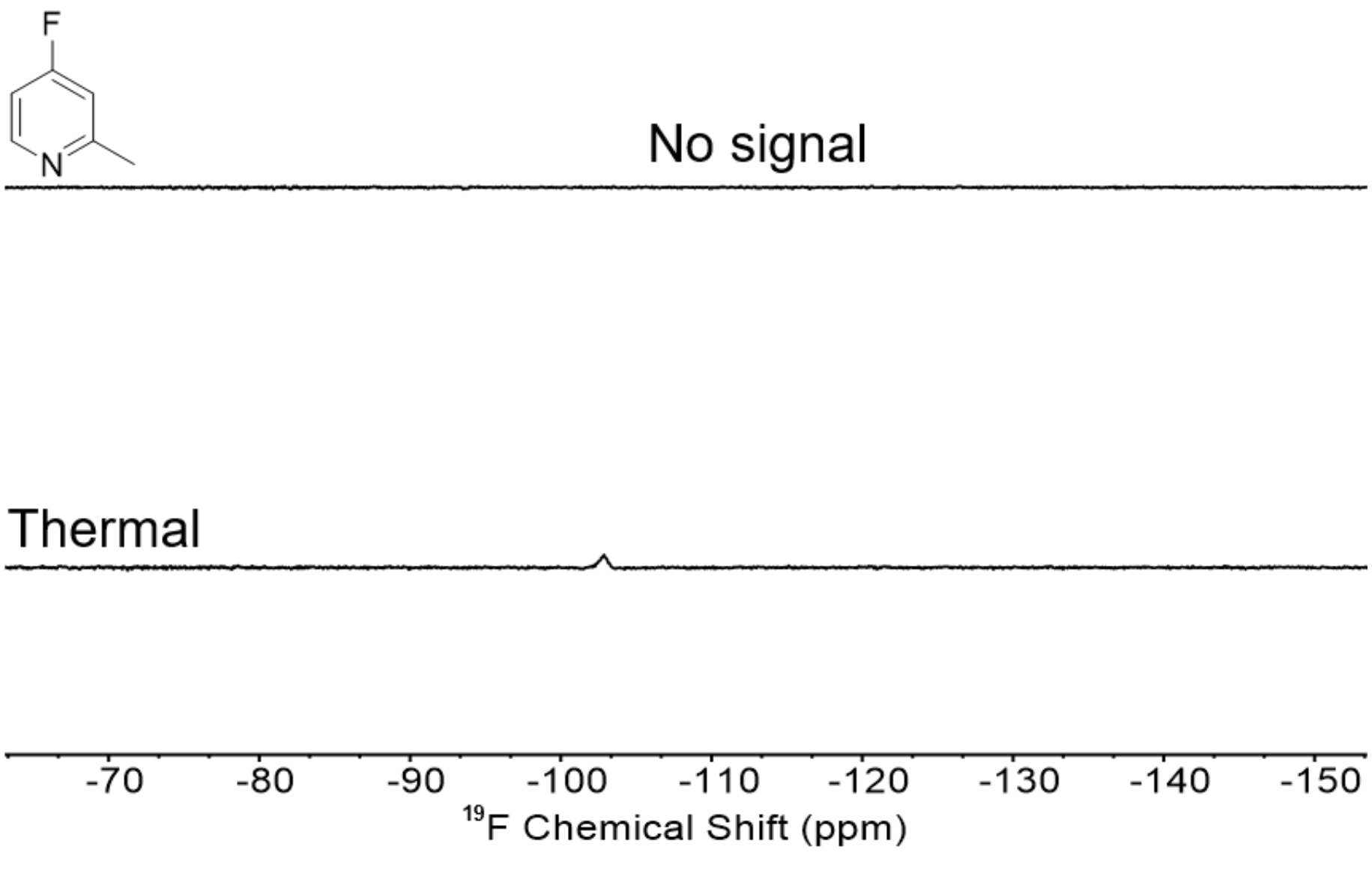

Fig. S63. ^{19}F NMR spectra of substrate **d3** at a concentration of 50 mM acquired under SABRE (top, 1 scan) and in the thermally polarized state (bottom, 64 scans).

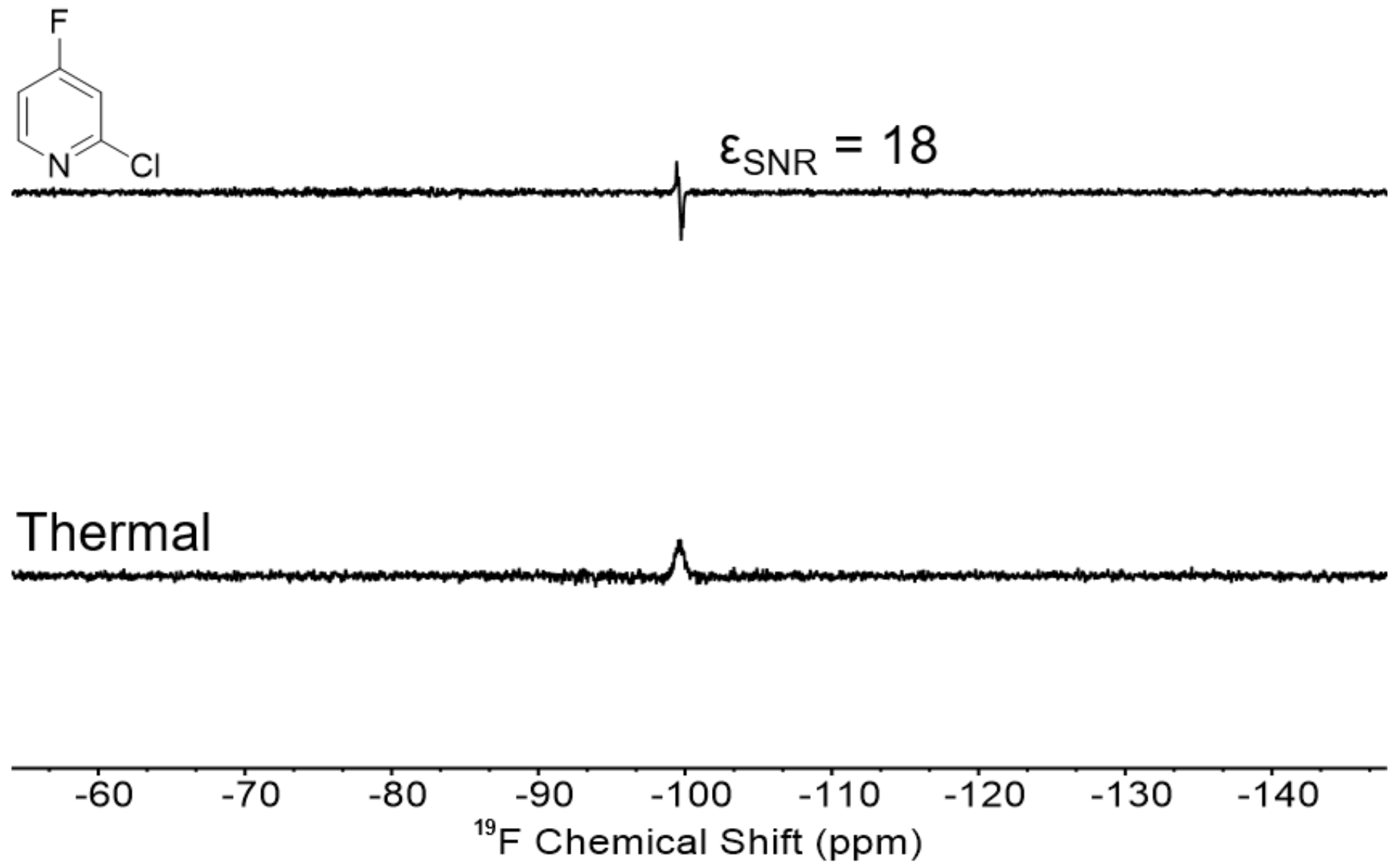

Fig. S64. ^{19}F NMR spectra of substrate **d4** at a concentration of 50 mM acquired under SABRE (top, 1 scan) and in the thermally polarized state (bottom, 64 scans).

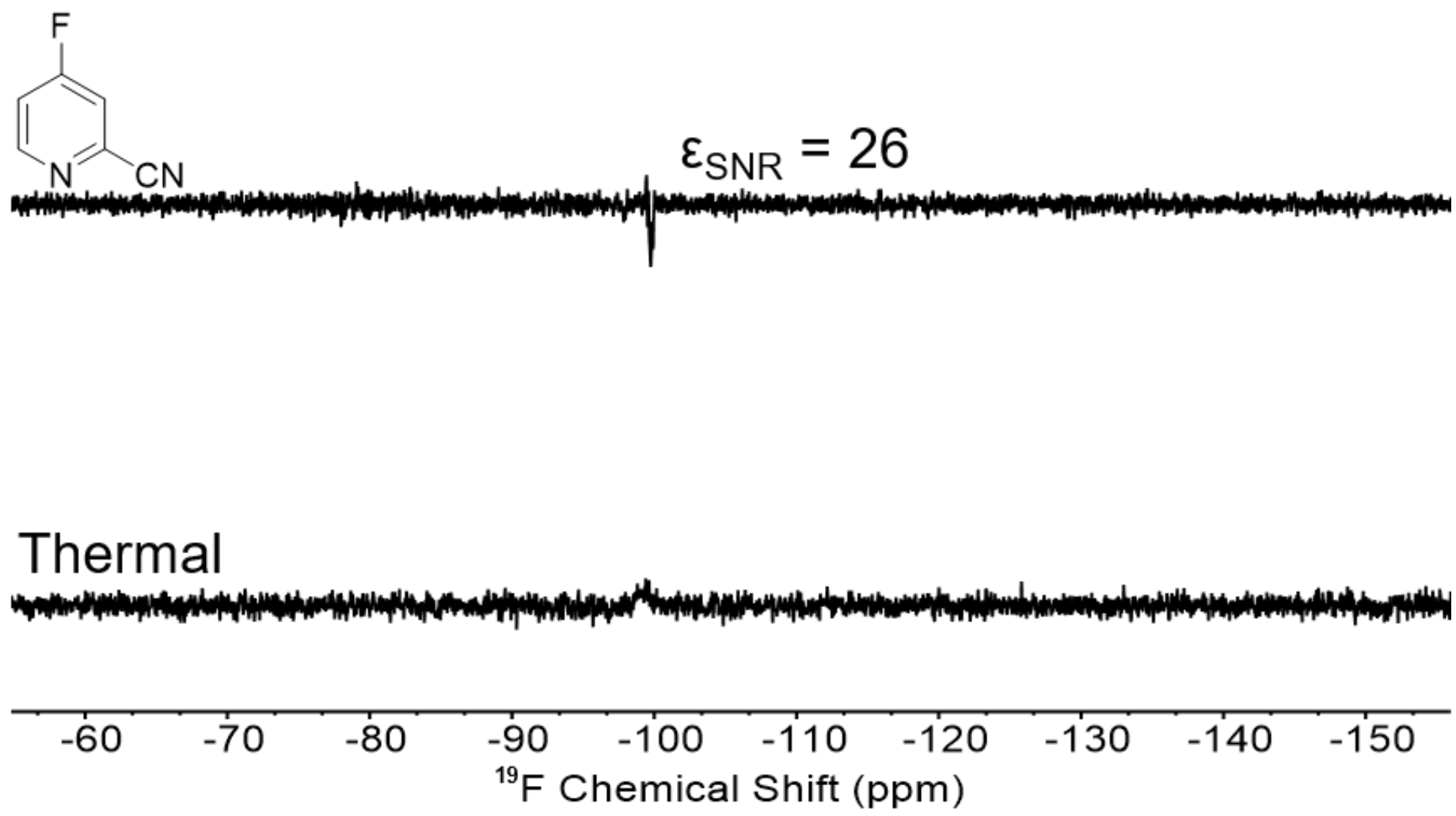

Fig. S65. ^{19}F NMR spectra of substrate **d5** at a concentration of 50 mM acquired under SABRE (top, 1 scan) and in the thermally polarized state (bottom, 64 scans).

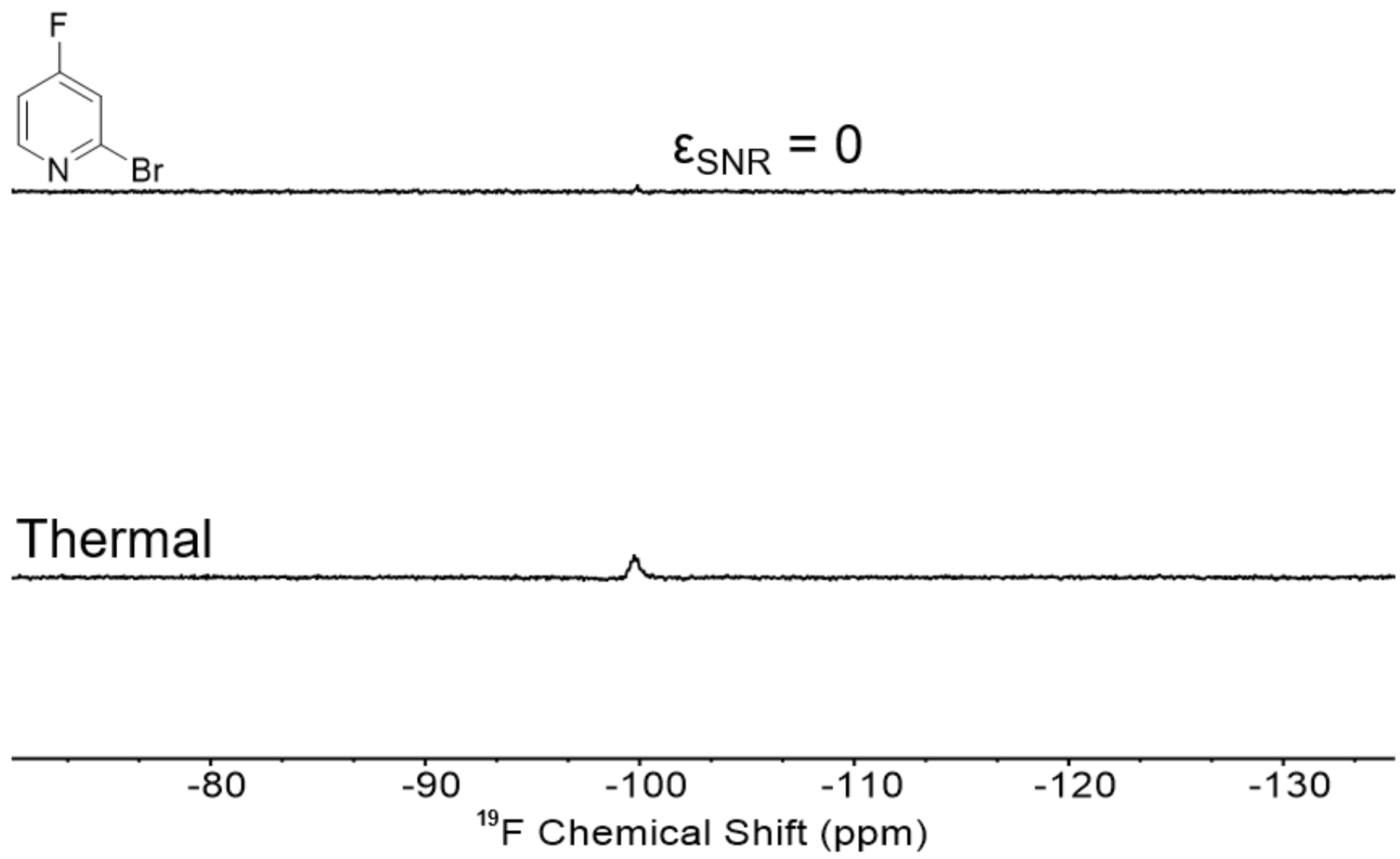

Fig. S66. ^{19}F NMR spectra of substrate **d6** at a concentration of 50 mM acquired under SABRE (top, 1 scan) and in the thermally polarized state (bottom, 64 scans).

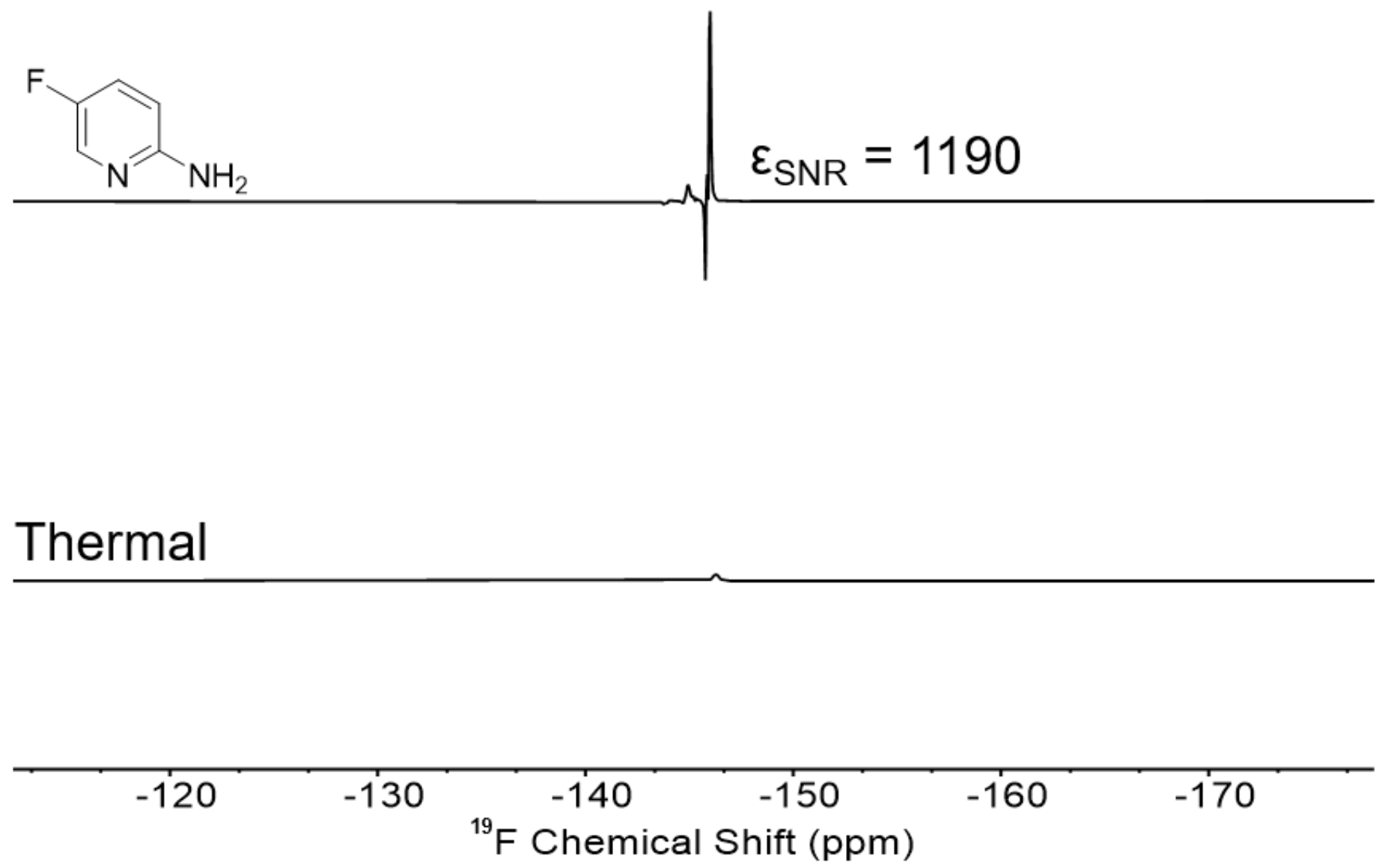

Fig. S67. ^{19}F NMR spectra of substrate **e1** at a concentration of 50 mM acquired under SABRE (top, 1 scan) and in the thermally polarized state (bottom, 64 scans).

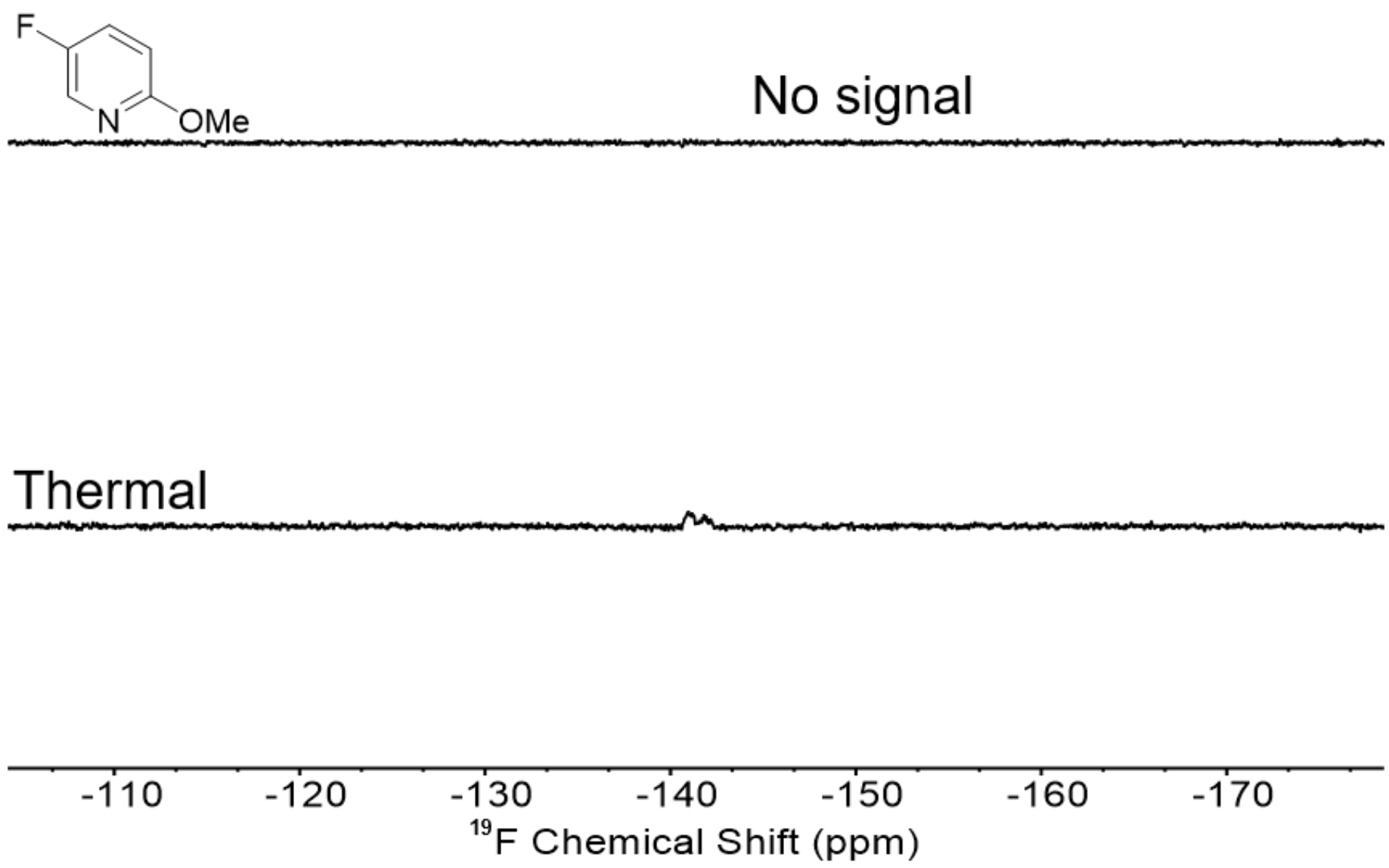

Fig. S68. ^{19}F NMR spectra of substrate **e2** at a concentration of 50 mM acquired under SABRE (top, 1 scan) and in the thermally polarized state (bottom, 64 scans).

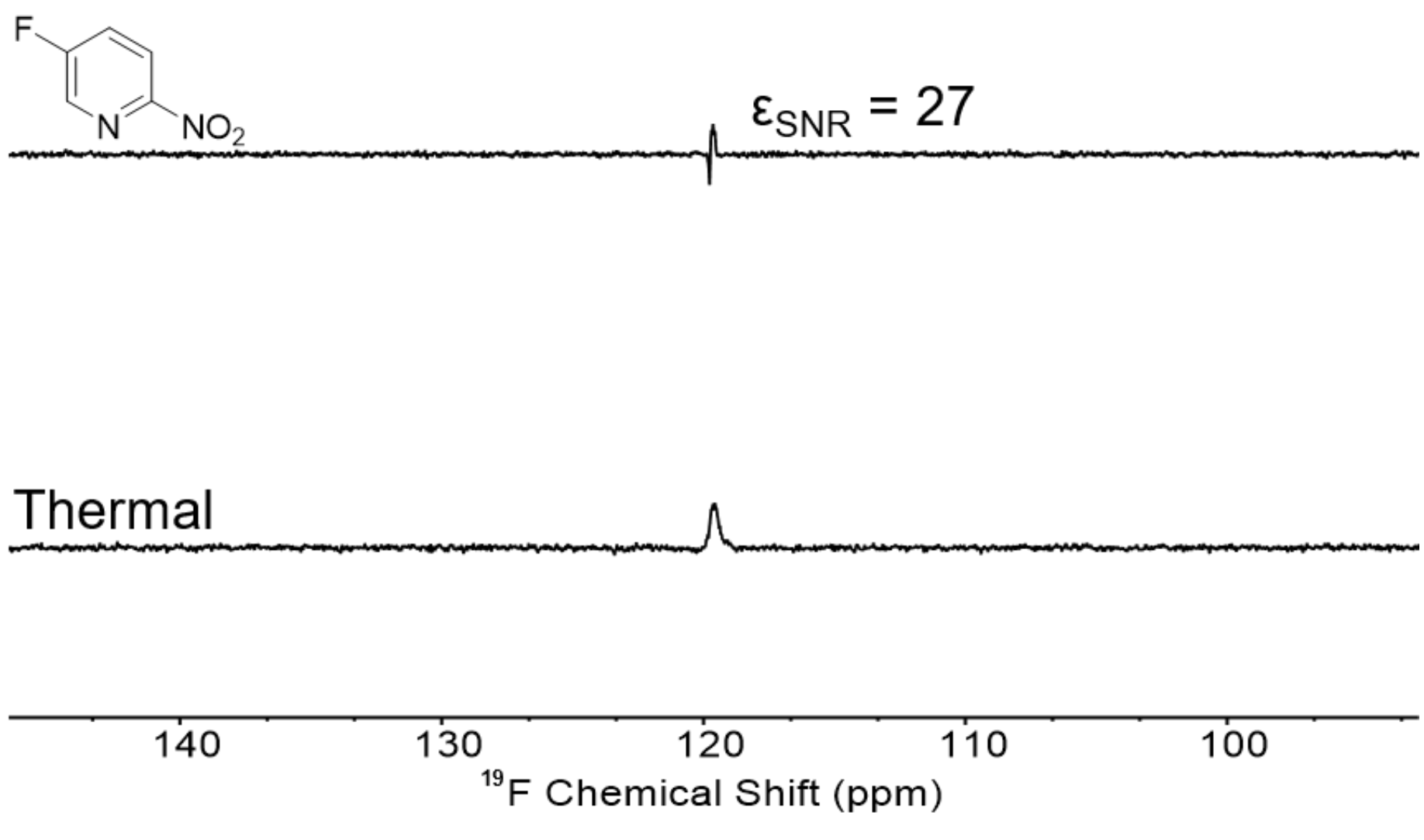

Fig. S69. ^{19}F NMR spectra of substrate **e3** at a concentration of 50 mM acquired under SABRE (top, 1 scan) and in the thermally polarized state (bottom, 64 scans).

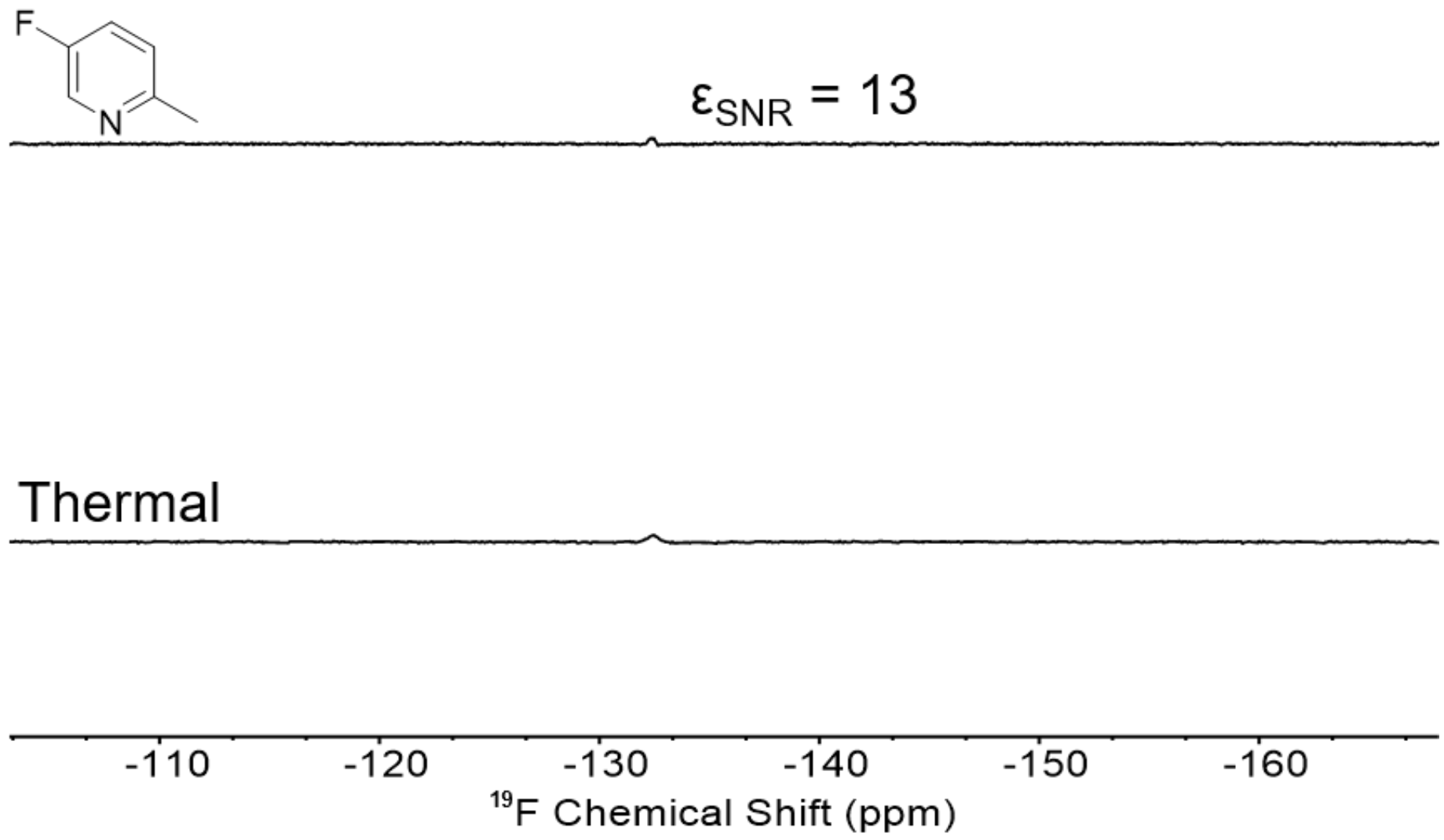

Fig. S70. ^{19}F NMR spectra of substrate **e4** at a concentration of 50 mM acquired under SABRE (top, 1 scan) and in the thermally polarized state (bottom, 64 scans).

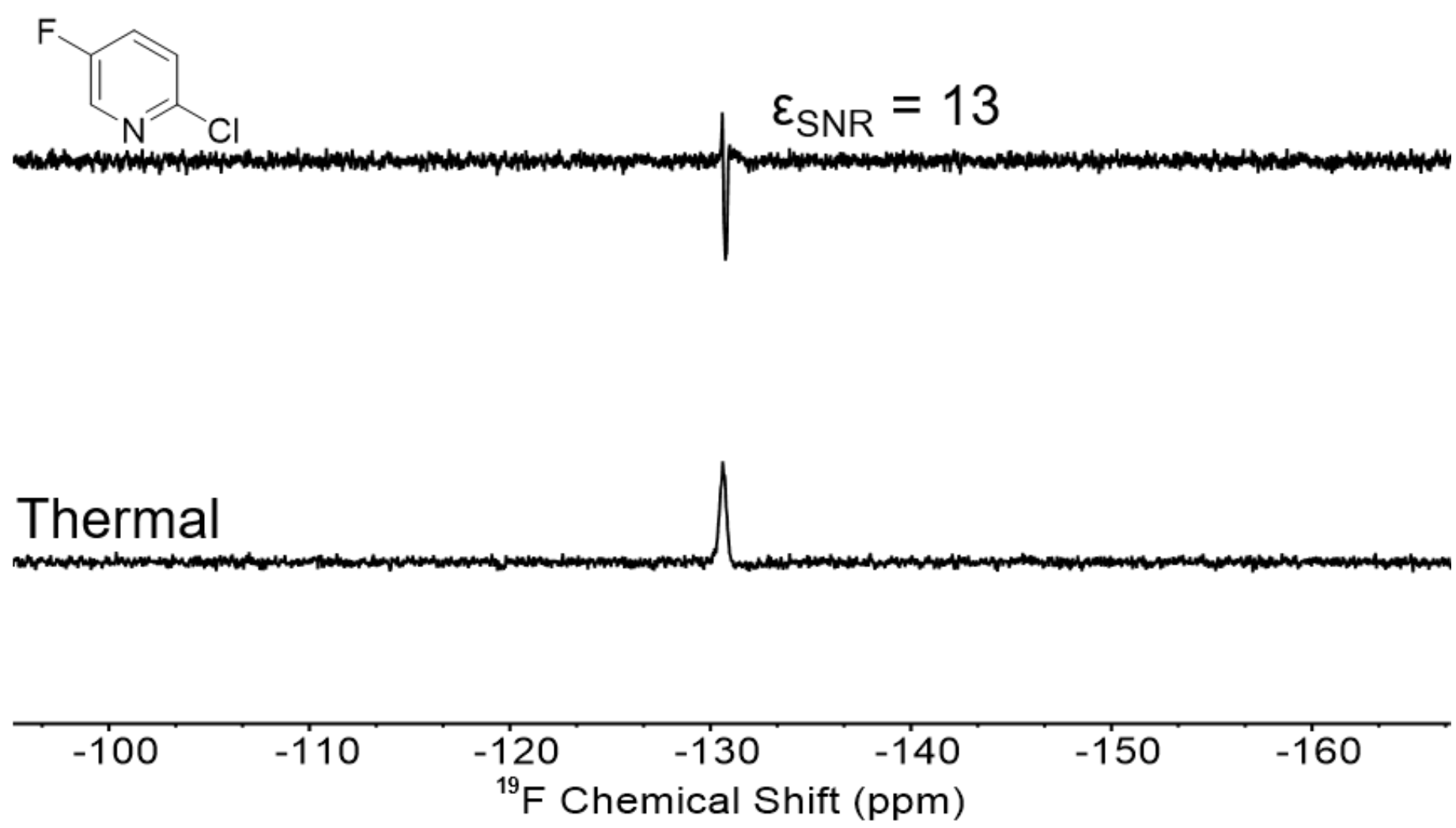

Fig. S71. ^{19}F NMR spectra of substrate **e5** at a concentration of 50 mM acquired under SABRE (top, 1 scan) and in the thermally polarized state (bottom, 64 scans).

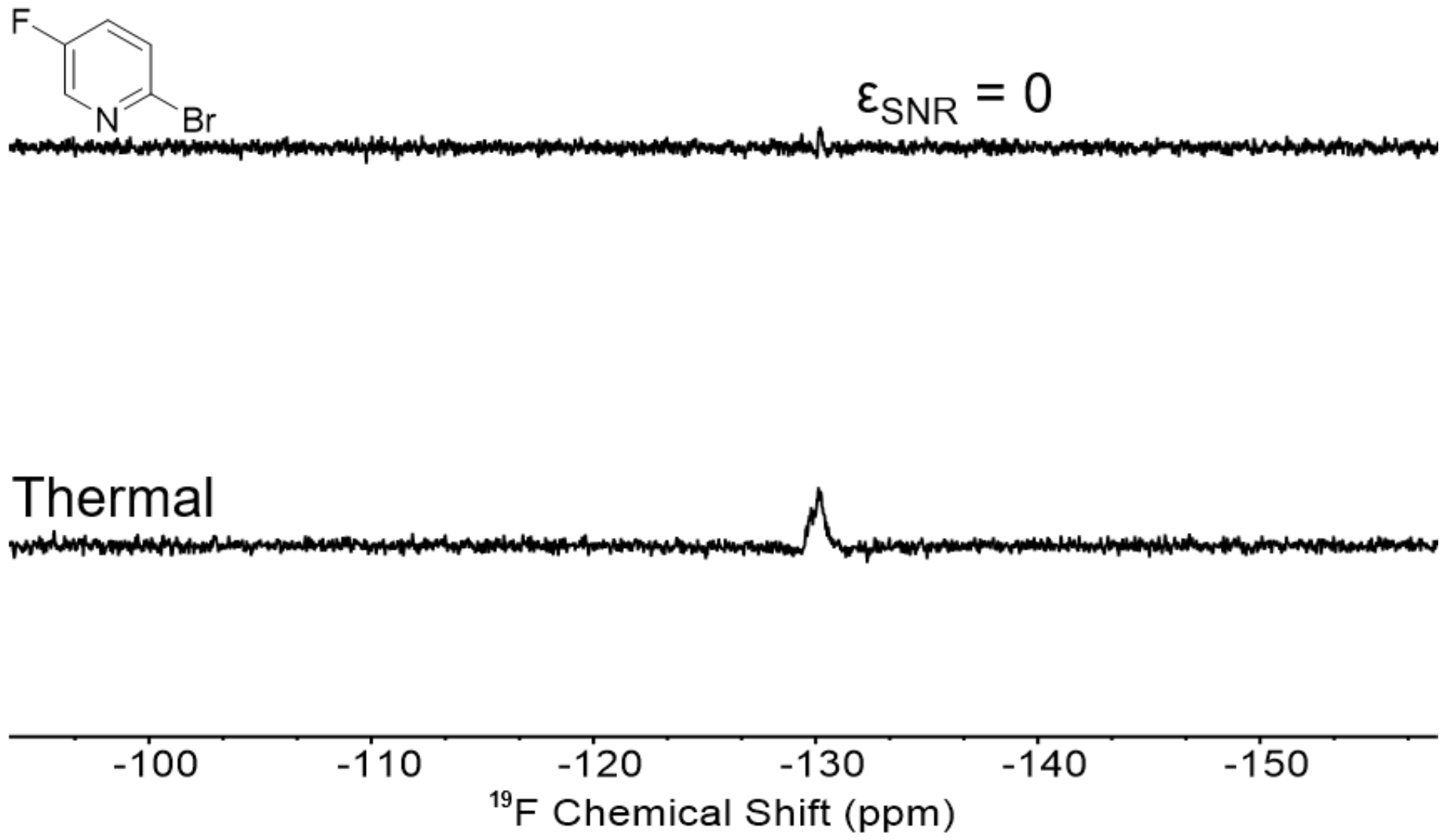

Fig. S72. ^{19}F NMR spectra of substrate **e6** at a concentration of 50 mM acquired under SABRE (top, 1 scan) and in the thermally polarized state (bottom, 64 scans).

6 Figures and Tables for Machine Learning

Table S1. DFT-derived molecular features of the fluoropyridine derivatives used in the random-forest classification and regression analyses.

Global Electronic Structure	Structural & Shape	Surface & Distribution	Local Reactivity
ODI_HOMO_1	AtomNum	Volume	Nu
ODI_HOMO	Weight	Density	Pi
ODI_LUMO	Farthest_Distance	ESPmin	MPI
ODI_LUMO_Add1	Mol_RadiusMol_Size_Short	ESPmax	ALIE_Ave
ODI_Mean	Mol_Size_2	Overall_Surface_Area	ALIE_Var
ODI_Std	Mol_Size_L	Pos_Surface_Area	LEAmin
HOMO	Length_Ratio	Neg_Surface_Area	LEAmax
LUMO	Len_Div_Diameter	Overall_Average	LEA_Ave
HOMO_number	MPP	Pos_Average	LEA_Var
HOMO_LUMO_Gap	SDP	Neg_Average	ADCH_Std
Dipole_Moment		Overall_Variance	ADCH_Min
Quadrupole_Moment		Nonpolar_Area	ADCH_Max
Octopole_Moment		Polar_Area	N_Total_Valence
			N_Sum_of_connectivity
			N_LP_Value

Table S2. Comparison of precision, recall, and f1-score for RF classifiers based on LOOCV under different enhancement thresholds.

	Threshold(Enhancement)	Precision	Recall	F1-Score
Low-enhancement	200	0.77	0.89	0.83
	250	0.77	0.89	0.83
	300	0.83	1.00	0.91
	350	0.83	1.00	0.91
	400	0.83	1.00	0.91
	500	0.77	0.95	0.85
	600	0.77	0.95	0.85
High-enhancement	200	0.82	0.64	0.72
	250	0.82	0.64	0.72
	300	1.00	0.69	0.82
	350	1.00	0.69	0.82
	400	1.00	0.69	0.82
	500	0.86	0.50	0.63
	600	0.86	0.50	0.63

Table S3. Quantitative classification metrics for LR and RF models based on LOOCV.

Model	Class	Precision	Recall	F1-Score
LR	Low-enhancement	0.95	0.90	0.92
	High-enhancement	0.86	0.92	0.89
RF	Low-enhancement	0.83	1.00	0.91
	High-enhancement	1.00	0.69	0.82

Table S4. Statistical significance and collinearity assessment of the molecular descriptors in the forward stepwise linear regression model.

	Neg_Average	HOMO_LUMO_Gap	Nu	ESPmax	Quadrupole_Moment
VIF	2.712	1.272	1.766	2.147	2.839
P-value	0.000	0.002	0.003	0.007	0.042

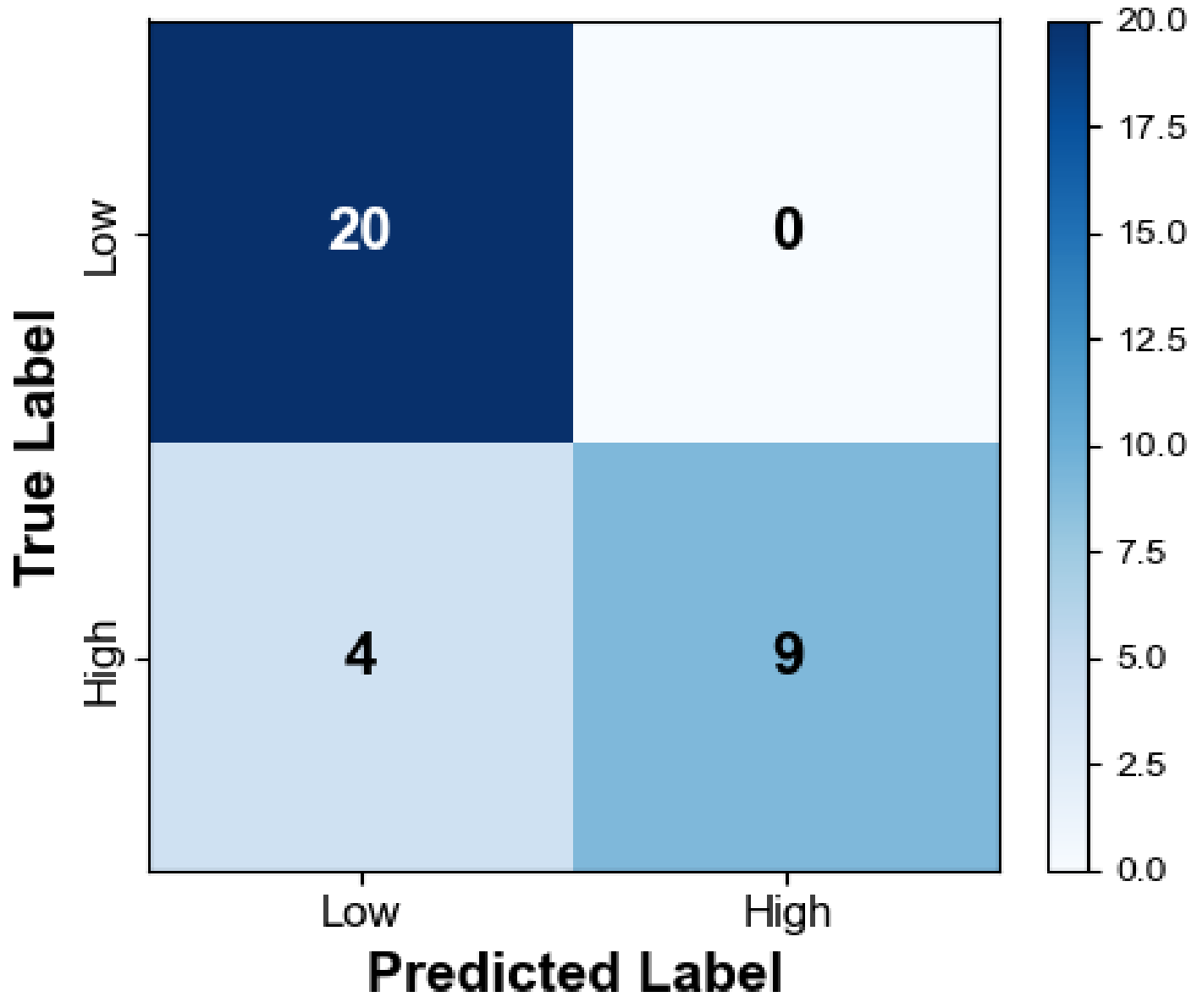

Fig. S73. Confusion matrix of the random forest (RF) classification model for molecular enhancement performance.

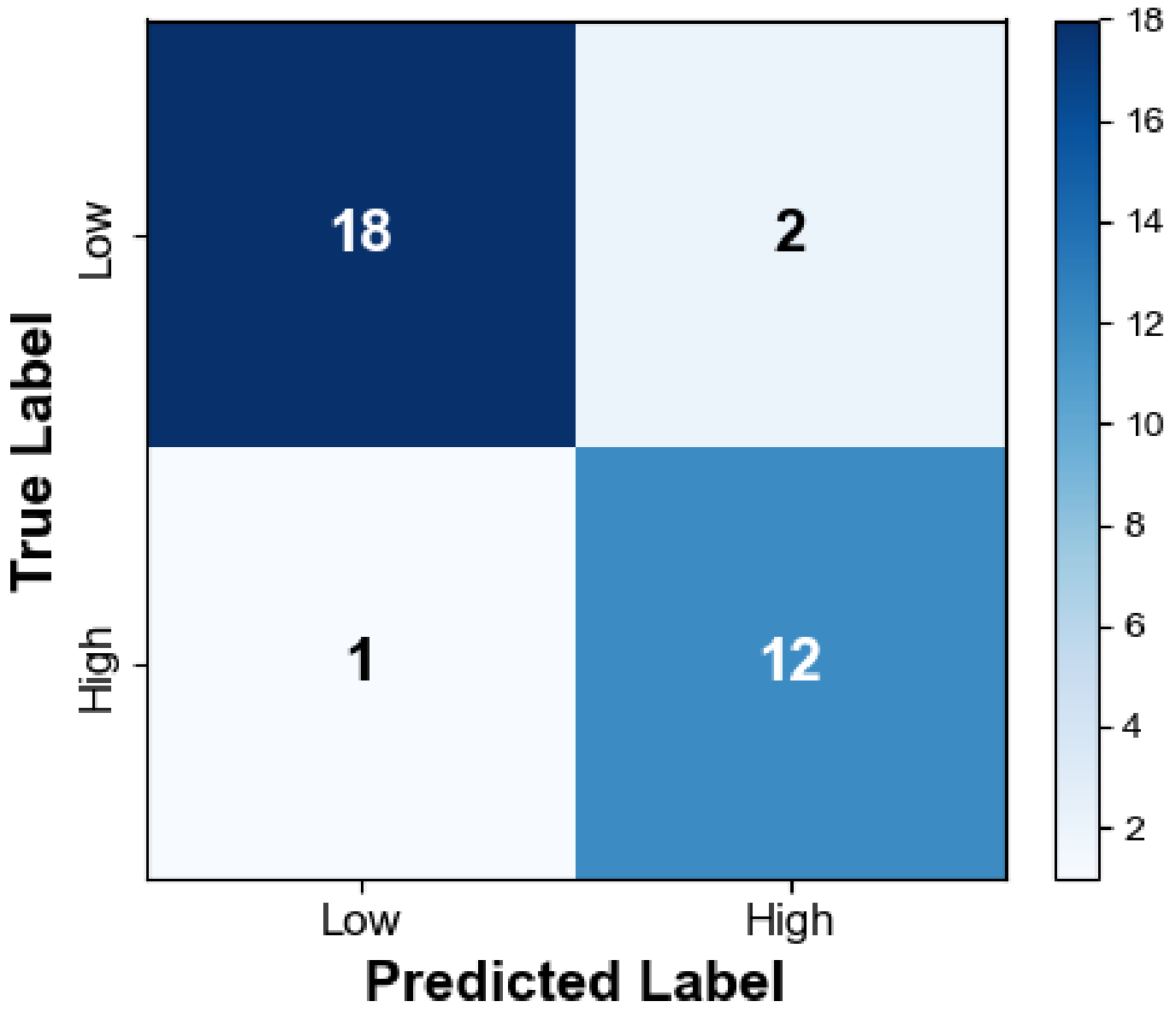

Fig. S74. Confusion matrix of the logistic regression (LR) classification model for molecular enhancement performance.

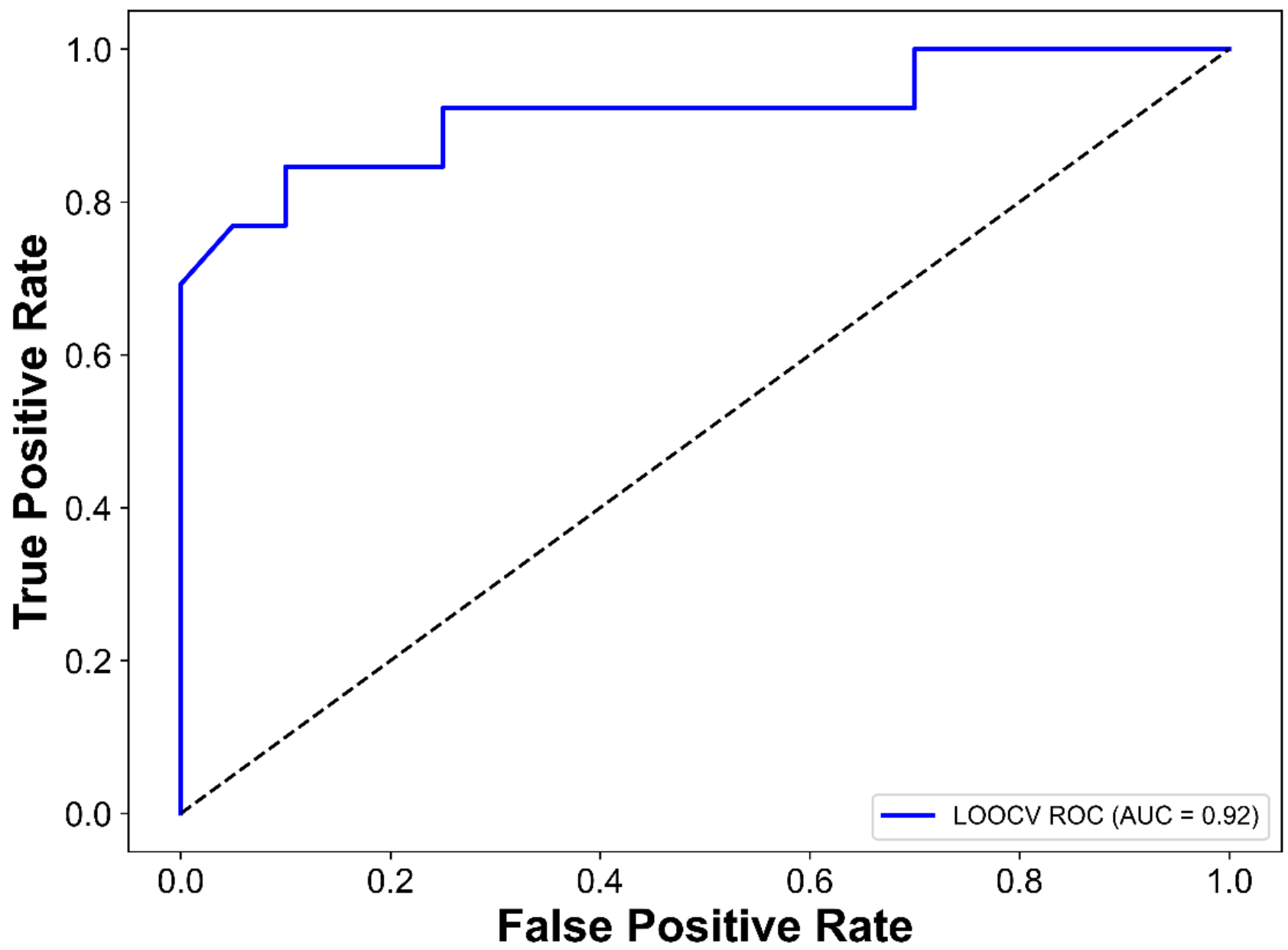

Fig. S75. Performance evaluation of the random forest classifier. The figure displays the ROC curve obtained via leave-one-out cross-validation (LOOCV), with an area under the curve (AUC) of 0.92.

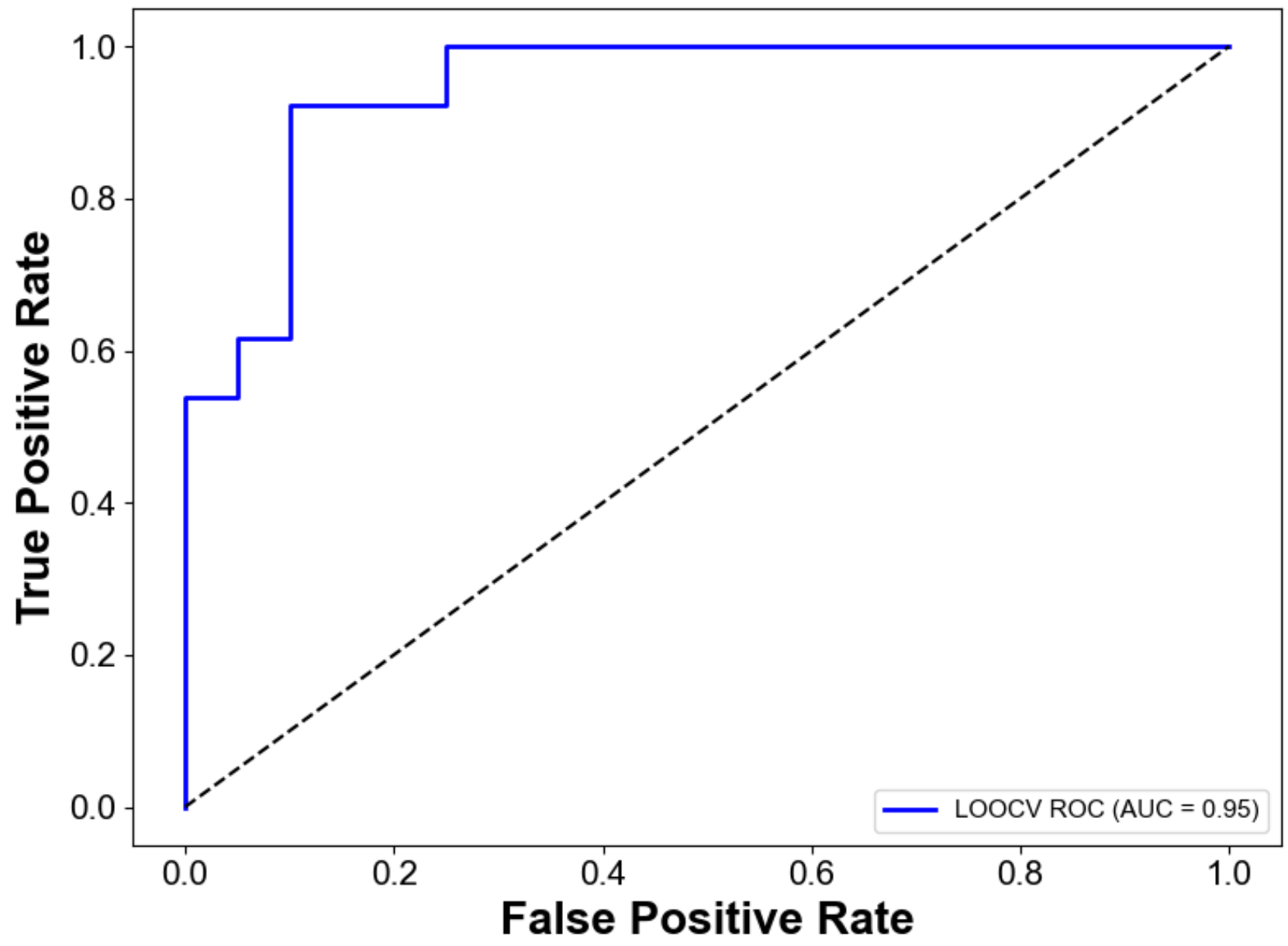

Fig. S76. Performance evaluation of the logistic regression classifier. The figure displays the ROC curve obtained via leave-one-out cross-validation (LOOCV), with an area under the curve (AUC) of 0.95.

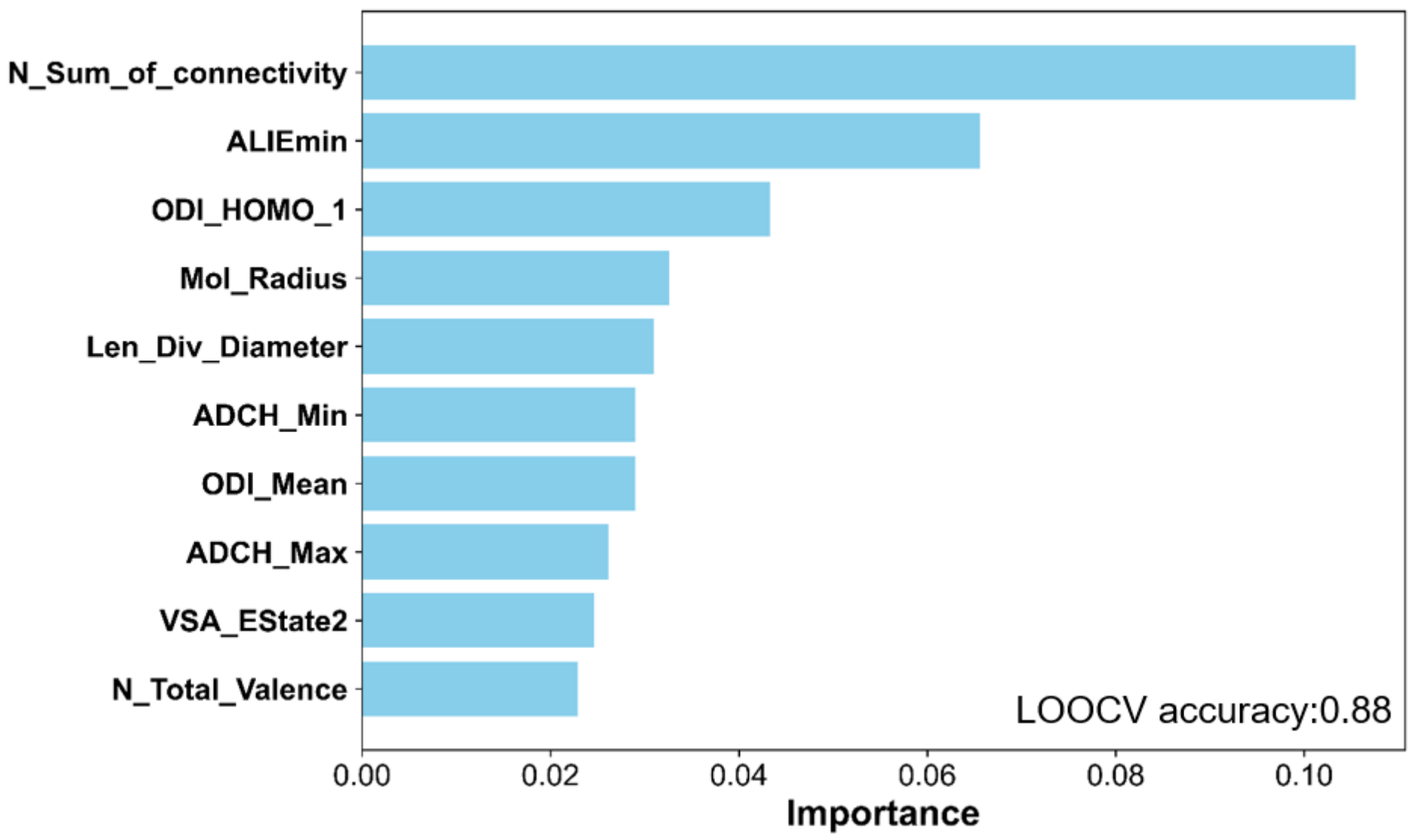

Fig. S77. Feature importance ranking derived from the RF classification model, with importance decreasing from top to bottom.

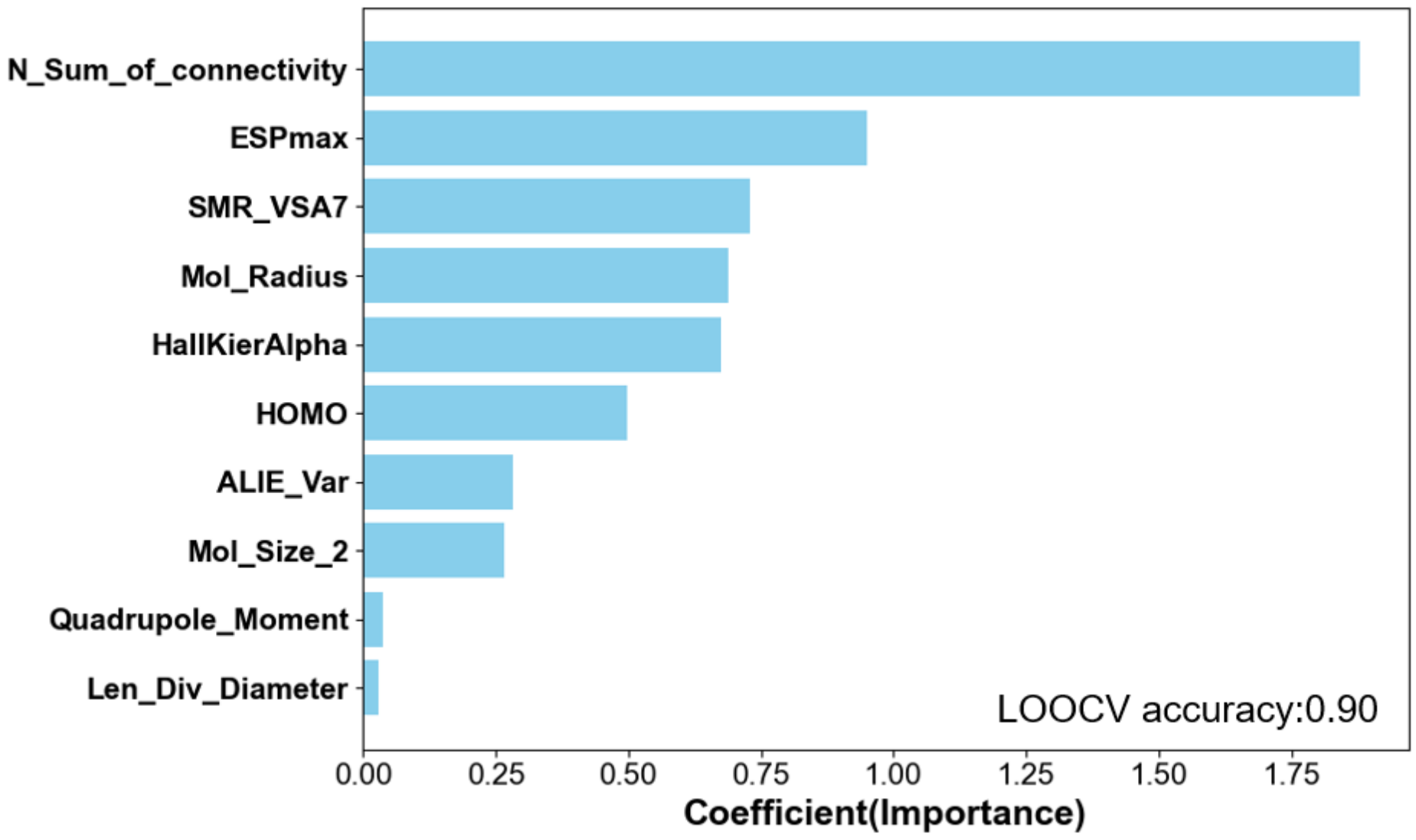

Fig. S78. Feature importance ranking derived from the LR classification model, with importance decreasing from top to bottom.

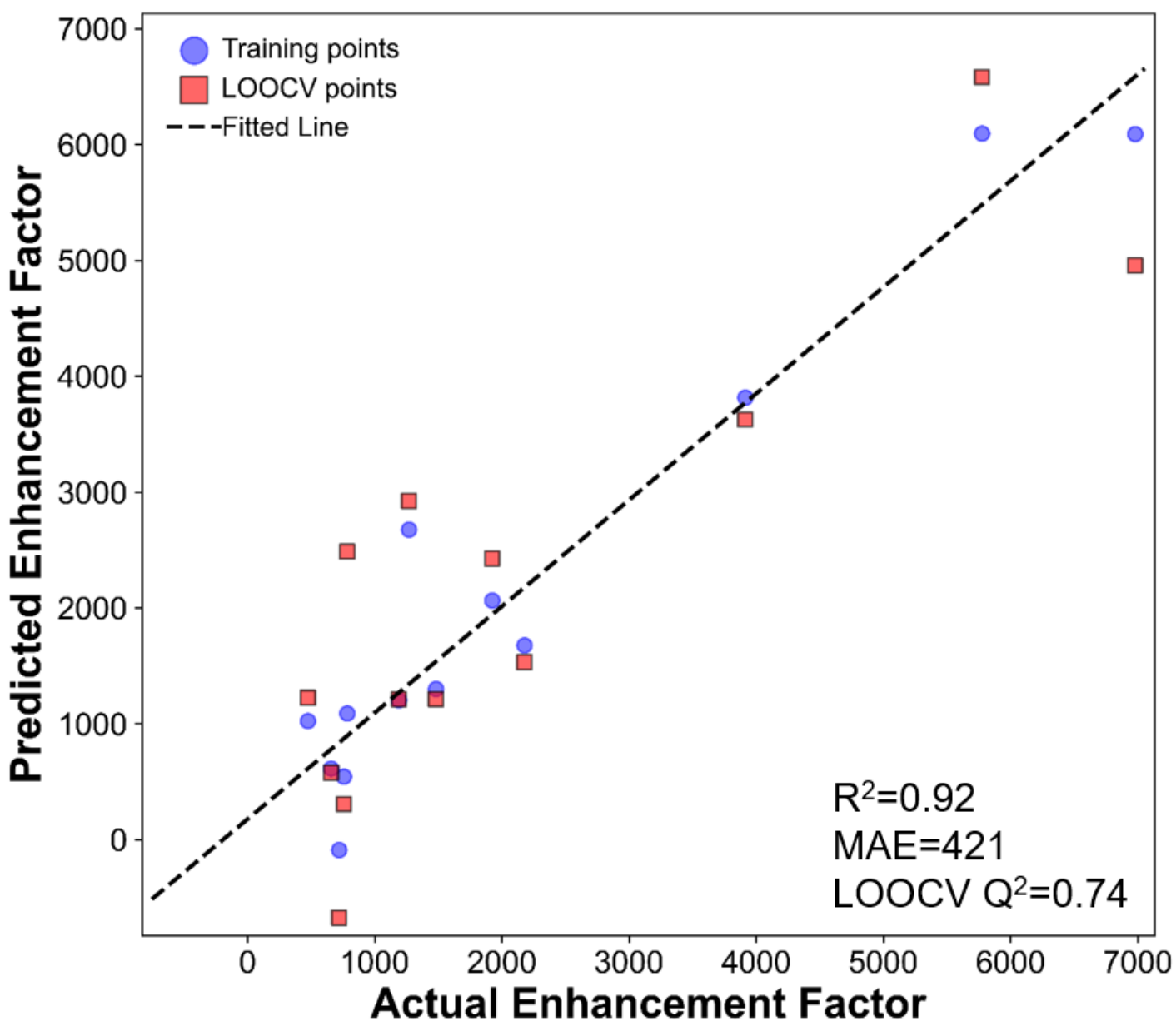

Fig. S79. Performance evaluation of the forward stepwise linear regression model. Blue circles represent the fitting results of the training set, while red squares represent the predictive performance from leave-one-out cross-validation (LOOCV).

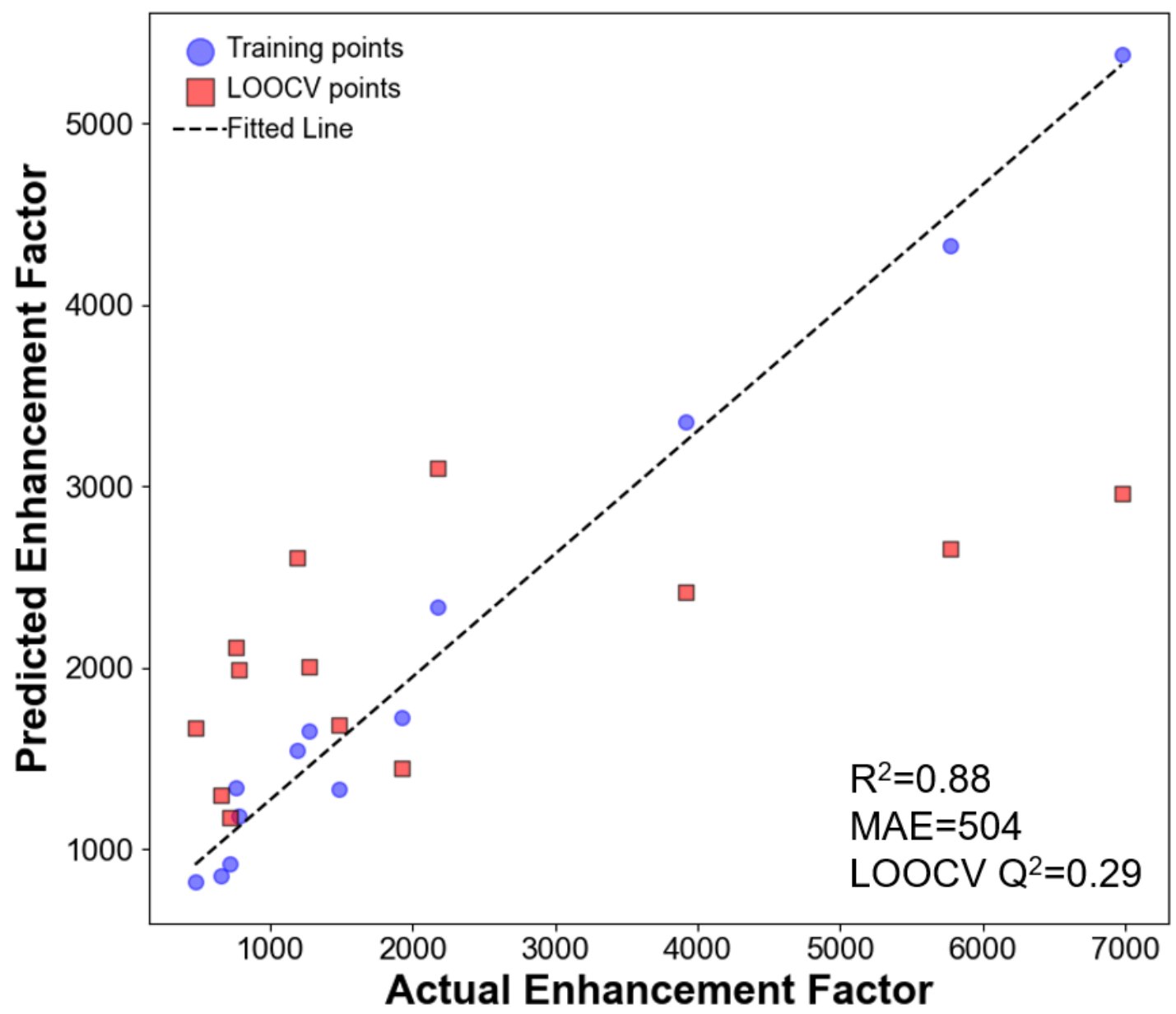

Fig. S80. Performance evaluation of the random forest (RF) regression model. Blue circles represent the fitting results of the training set, while red squares represent the predictive performance from leave-one-out cross-validation (LOOCV).

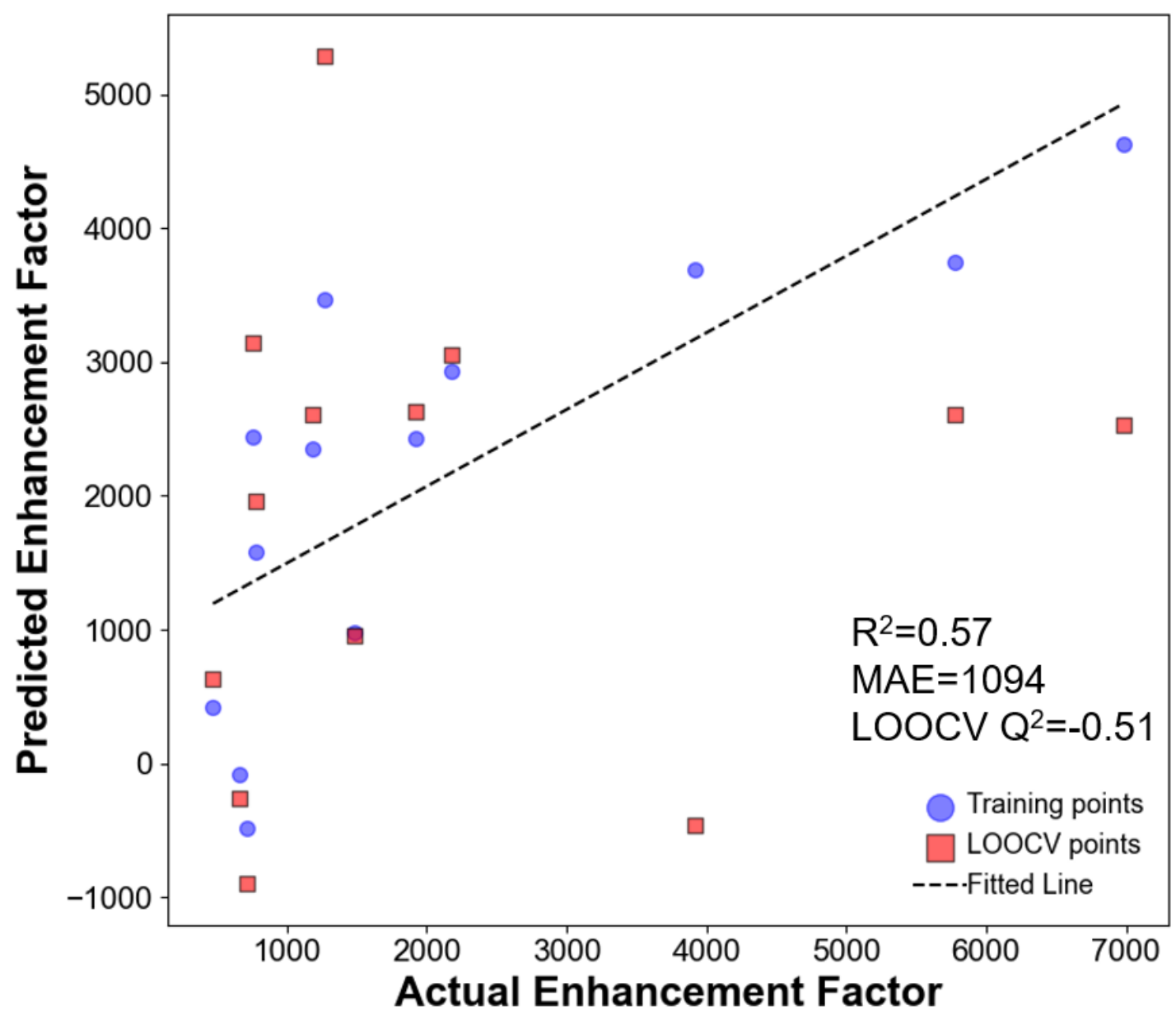

Fig. S81. Performance evaluation of the partial least squares (PLS) regression model. Blue circles represent the fitting results of the training set, while red squares represent the predictive performance from leave-one-out cross-validation (LOOCV).

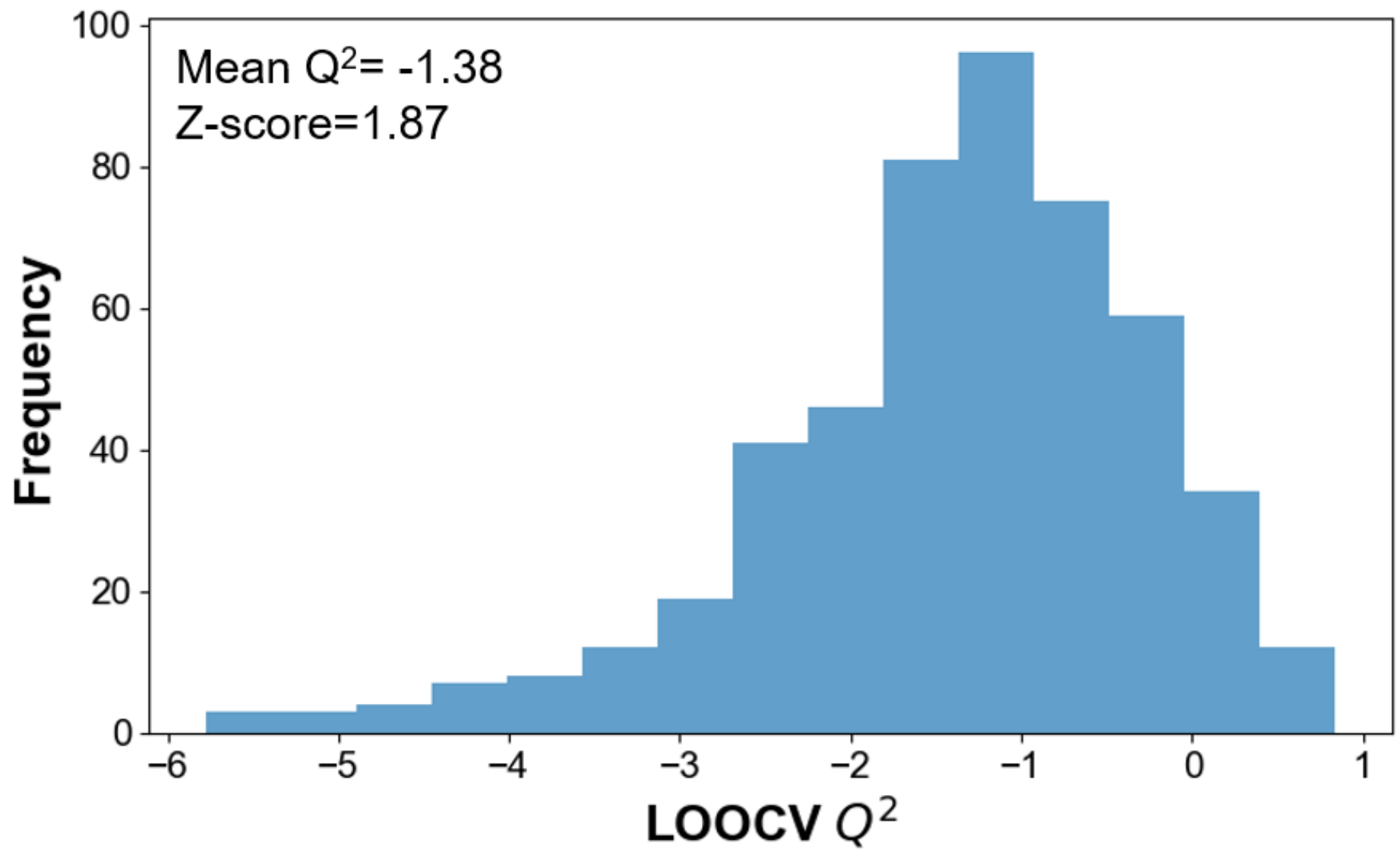

Fig. S82. Y-randomization test for the forward stepwise linear regression model. The histogram illustrates the distribution of LOOCV Q^2 values obtained from 500 random permutations of the response $\varepsilon_{\mathrm{SNR}}$.

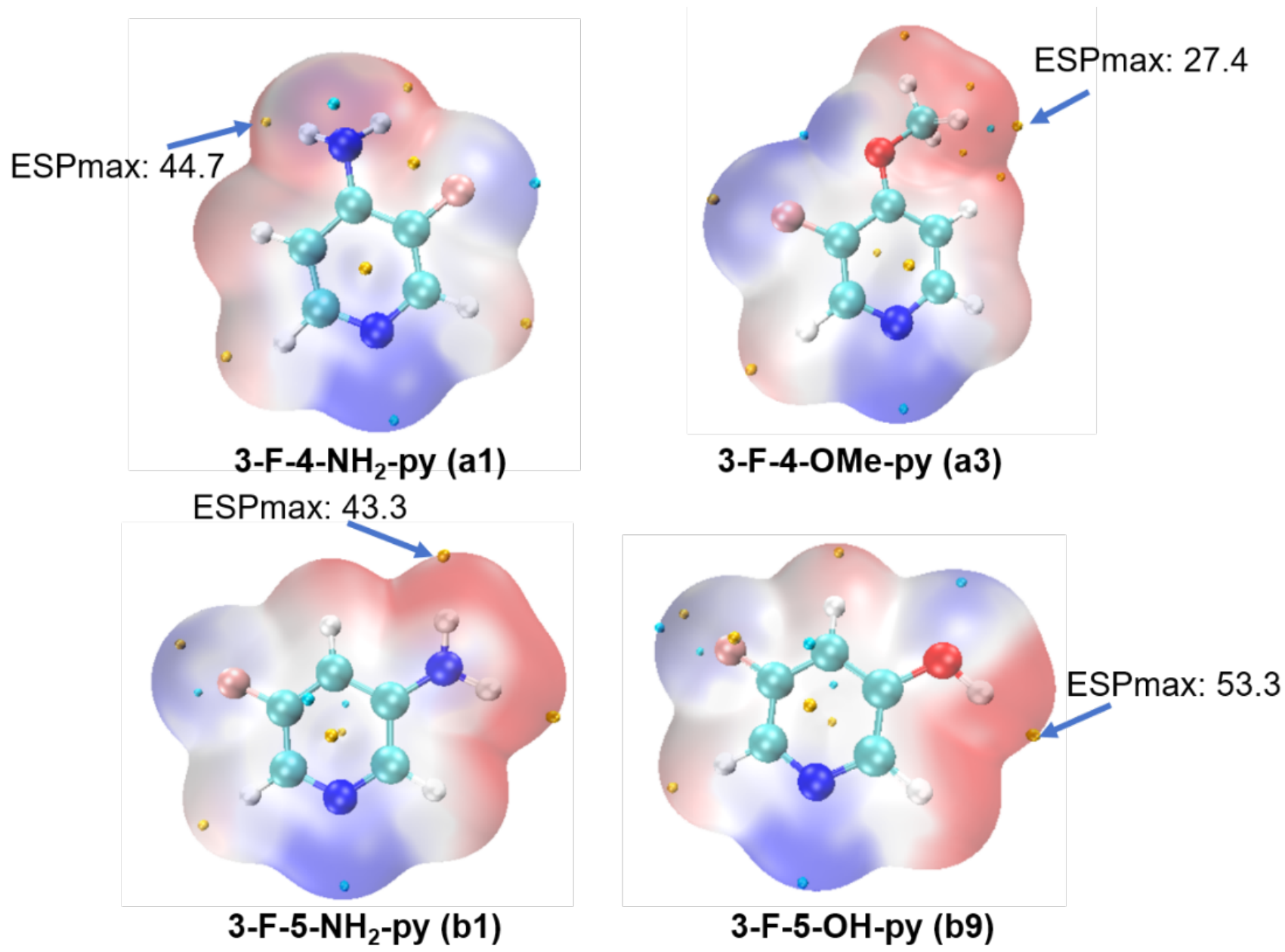

Fig. S83. Molecular electrostatic potential (ESP) surfaces of representative fluoropyridine derivatives, with arrows highlighting the maximum ESP values on the molecular surfaces.

7 Chemically Informed Parameters Relevant to ^{19}F SABRE Enhancement

Table S5. Ortho-substitution status, ^{19}F-T_1 relaxation times, dissociation rates at 298 K, and experimental ^{19}F SABRE enhancement factors for substrates **a1–e3**.

	Ortho-substitution	^{19}F-T_1(s)	Dissociation rate(s^{-1})	Actual ε_{SNR}
**a1**	No	2.3	11.09 ± 0.44	5775
**a2**	No	3.1	23.09 ± 0.42	6979
**a3**	No	2.9	23.75 ± 1.25	291
**a4**	No	3.9	23.01 ± 0.24	1481
**a5**	No	3.5	20.28 ± 0.18	474
**b1**	No	3.0	13.54 ± 0.12	2178
**b2**	No	3.9	20.83 ± 0.40	1269
**b3**	No	4.1	22.32 ± 0.75	113
**b4**	No	4.1	25.79 ± 0.48	785
**b5**	No	3.5	27.86 ± 1.31	658
**b7**	No	4.5	28.71 ± 0.43	721
**b8**	No	3.0	18.27 ± 0.16	3915
**b9**	No	3.1	19.08 ± 0.65	1925
**c1**	Yes	4.2	20.07 ± 0.18	759
**c2**	Yes	4.2	42.72 ± 2.79	0
**c3**	Yes	3.1	37.18 ± 1.10	0
**d1**	Yes	3.2	57.44 ± 1.58	0
**d2**	Yes	3.8	42.89 ± 3.71	28
**e1**	Yes	4.0	17.04 ± 0.51	1190
**e2**	Yes	4.4	44.35 ± 1.65	0
**e3**	Yes	4.0	43.39 ± 1.22	27

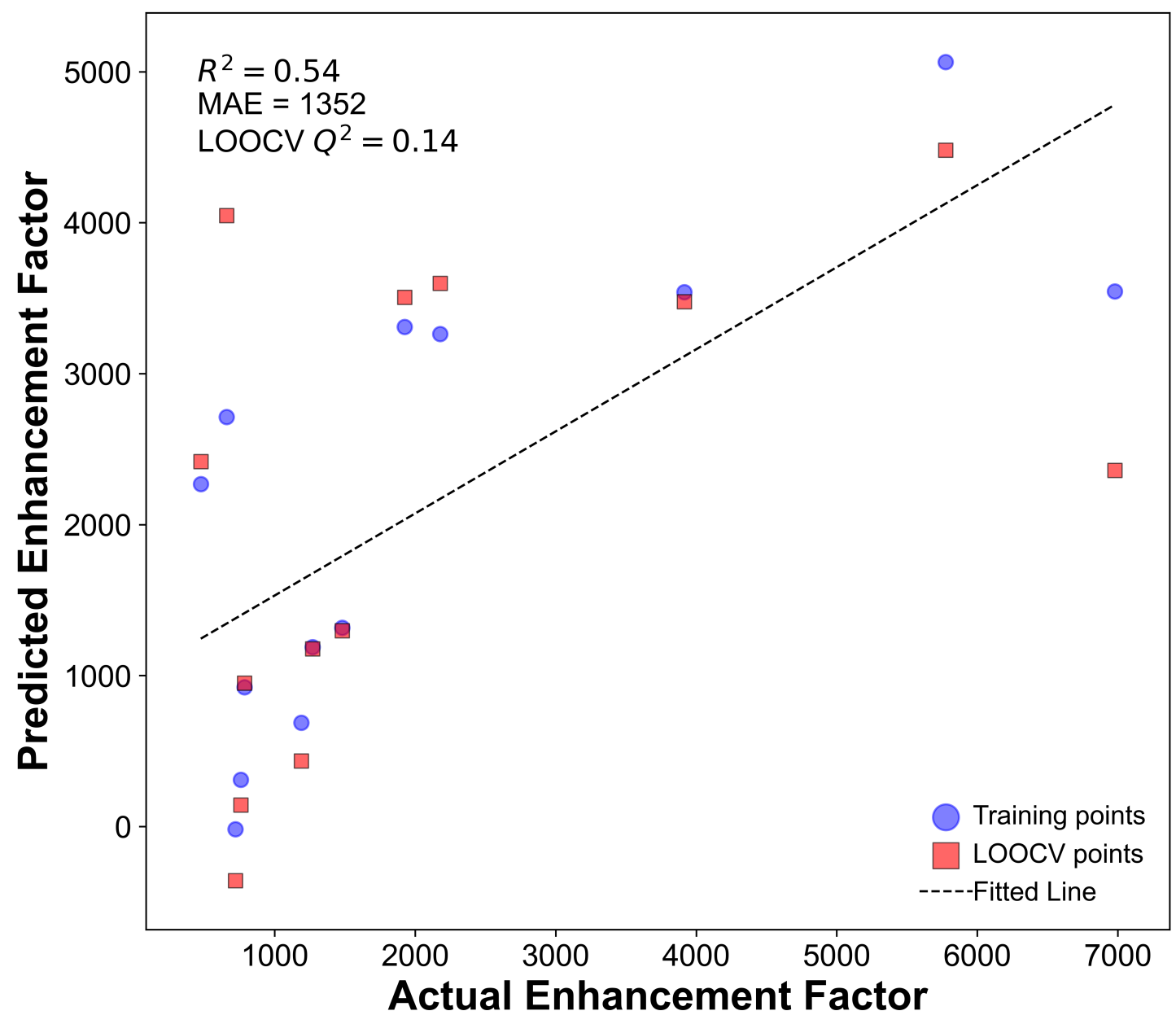

Fig. S84. Chemically informed baseline model for the high-enhancement subset. The model uses ^{19}F–T_1 and the substrate dissociation rate as predictors. The ordinary least-squares (OLS) fit gives R^2 = 0.54, while leave-one-out cross-validation (LOOCV) gives Q^2 = 0.14.The blue circles represent the OLS fit, the red squares represent the LOOCV predictions.

8 Hyperpolarized NMR Spectra and Predicted Results for Group F (Test Set)

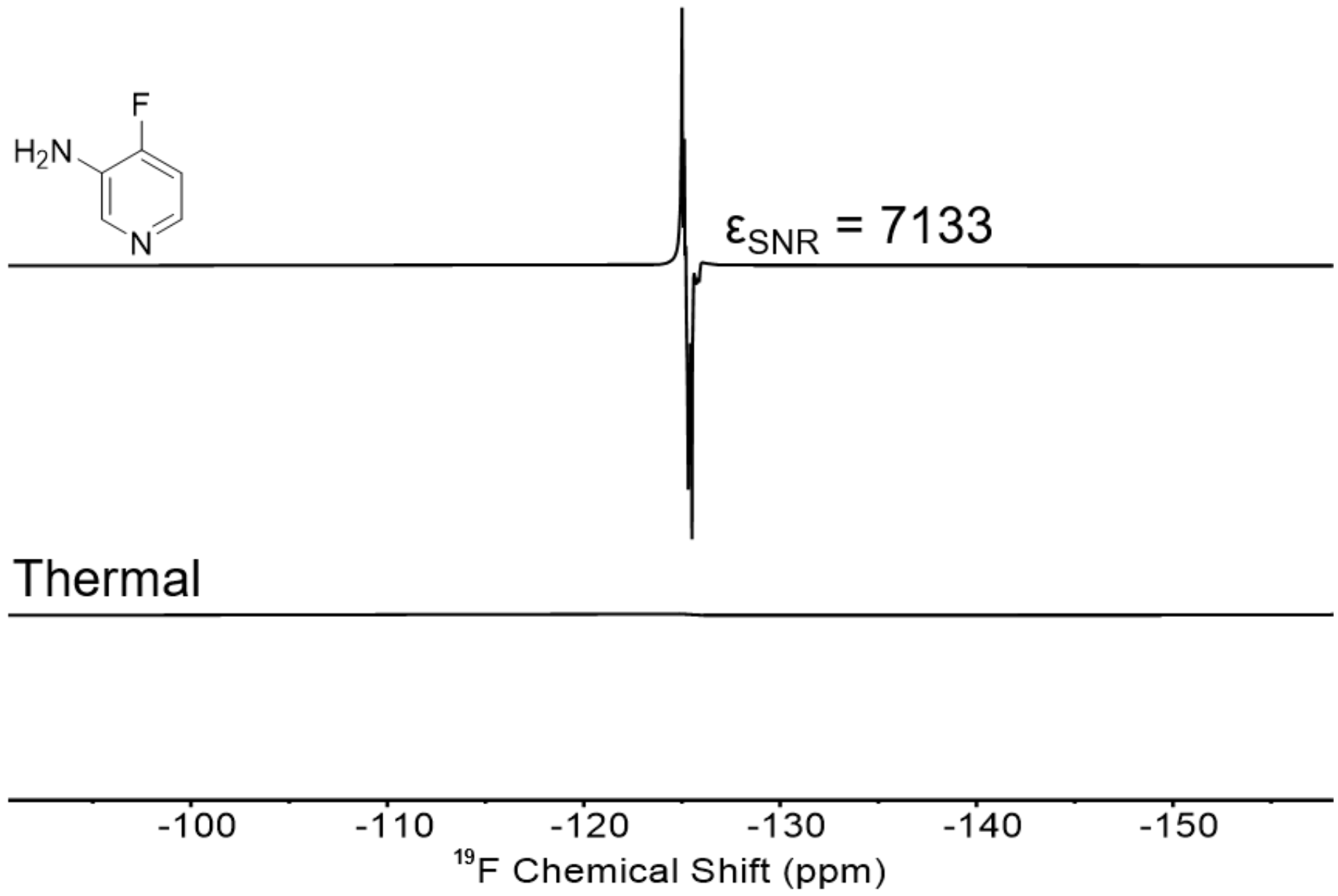

Fig. S85. ^{19}F NMR spectra of substrate **f1** at a concentration of 50 mM acquired under SABRE (top, 1 scan) and in the thermally polarized state (bottom, 64 scans).

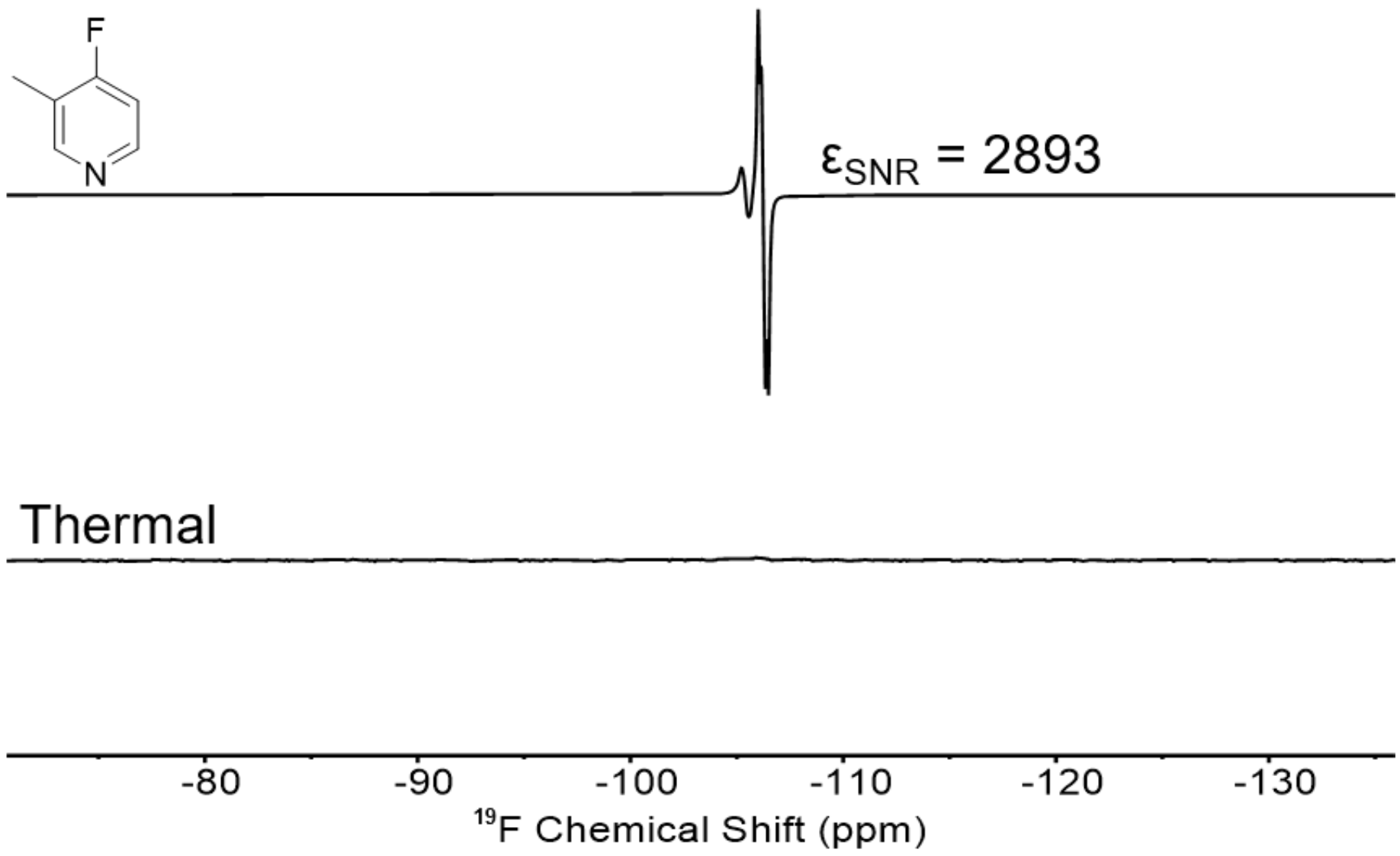

Fig. S86. ^{19}F NMR spectra of substrate **f2** at a concentration of 50 mM acquired under SABRE (top, 1 scan) and in the thermally polarized state (bottom, 64 scans).

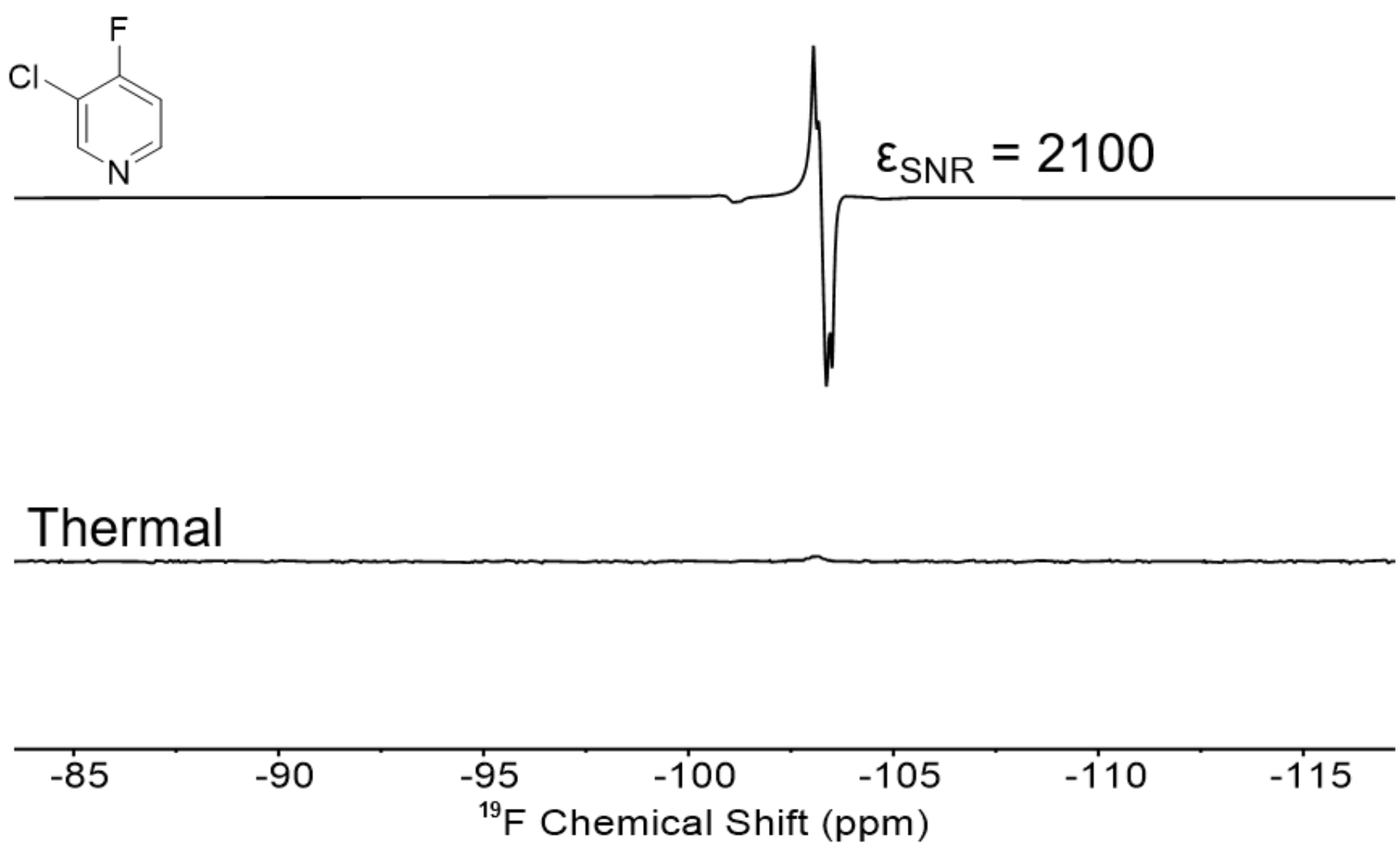

Fig. S87. ^{19}F NMR spectra of substrate **f3** at a concentration of 50 mM acquired under SABRE (top, 1 scan) and in the thermally polarized state (bottom, 64 scans).

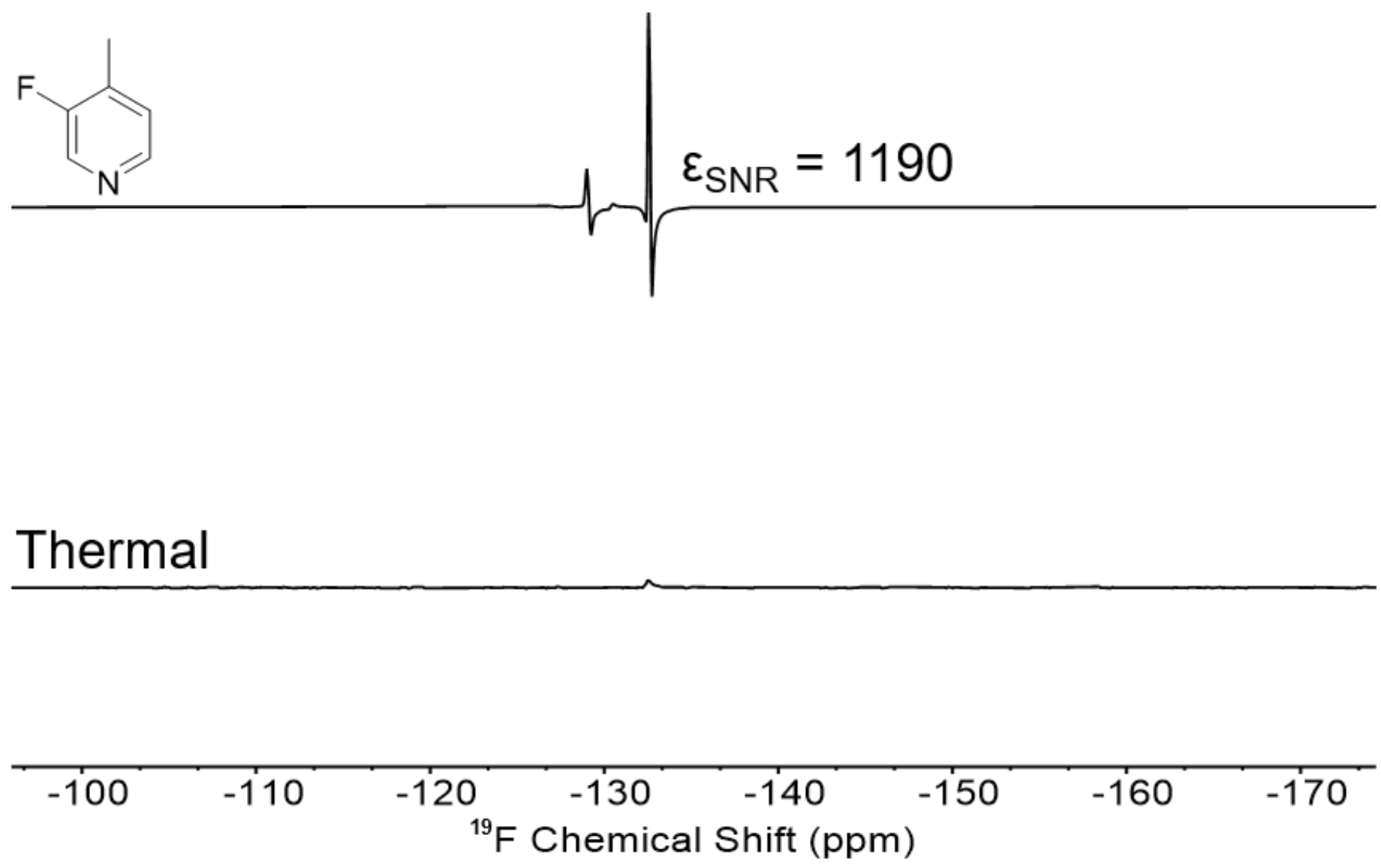

Fig. S88. ^{19}F NMR spectra of substrate **f4** at a concentration of 50 mM acquired under SABRE (top, 1 scan) and in the thermally polarized state (bottom, 64 scans).

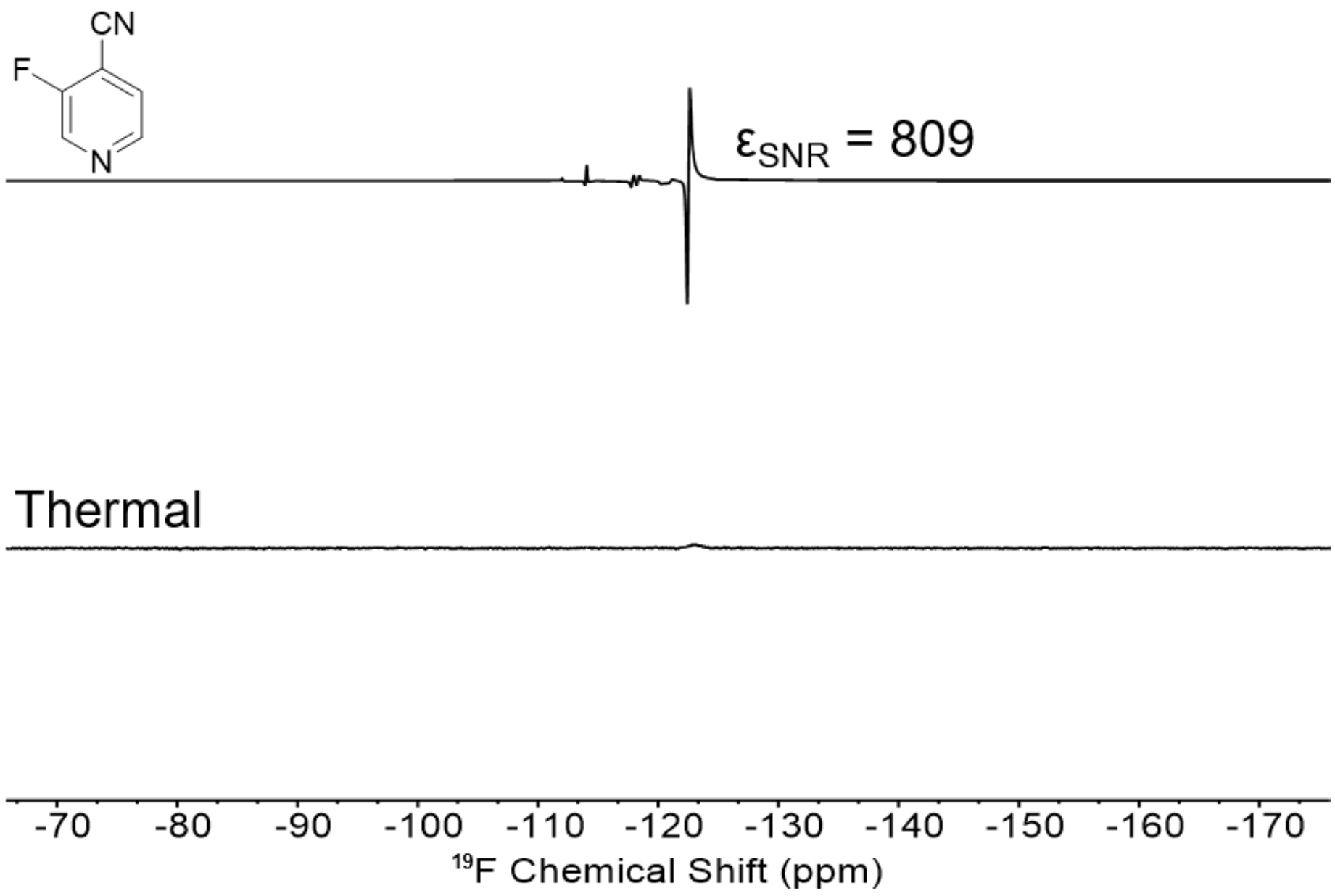

Fig. S89. ^{19}F NMR spectra of substrate **f5** at a concentration of 50 mM acquired under SABRE (top, 1 scan) and in the thermally polarized state (bottom, 64 scans).

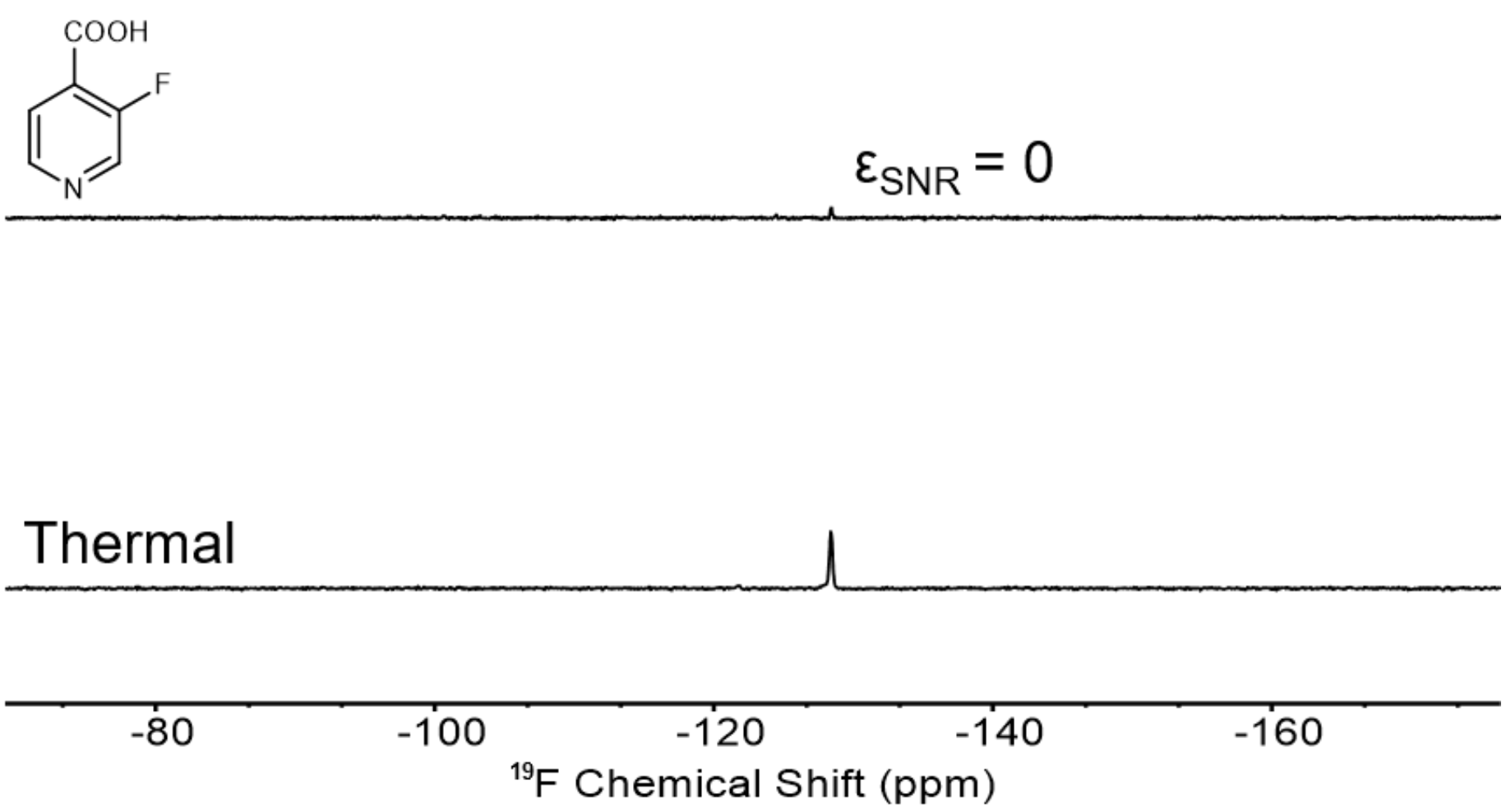

Fig. S90. ^{19}F NMR spectra of substrate **f6** at a concentration of 50 mM acquired under SABRE (top, 1 scan) and in the thermally polarized state (bottom, 64 scans).

Table S6. Molecular descriptors, RF classification, predicted ε_{SNR}, and actual ε_{SNR} of group F (test set).

	Neg_Average	HOMO_LUMO_Gap	Nu	ESPmax	Quadrupole_Moment	RF Classification	Predicted ε_{SNR}	Actual ε_{SNR}
**f1**	-0.02078541	0.223356	0.2489673	33.42503	13.990749	High	6637	7133
**f2**	-0.01614498	0.236456	0.1661086	21.04452	9.871251	Low	-2215	2893
**f3**	-0.01287705	0.227218	0.2486605	26.23597	8.371439	High	1068	2100
**f4**	-0.01650747	0.226013	0.1889723	21.90577	4.616845	High	828	1190
**f5**	-0.02146512	0.201135	0.2312571	30.14392	14.638429	Low	3466	809
**f6**	-0.02264307	0.192976	0.2498872	53.58759	9.252311	Low	9955	0

References

1 O. G. Salnikov, C. D. Assaf, A. P. Yi, S. B. Duckett, E. Y. Chekmenev, J.-B. Hovener, I. V. Koptyug and A. N. Pravdivtsev, *Anal. Chem.*, 2024, **96**, 11790–11799.

2 E. V. Stanbury, P. M. Richardson and S. B. Duckett, *Catal. Sci. Technol.*, 2019, **9**, 3914–3922.

3 K. X. Moreno, K. Nasr, M. Milne, A. D. Sherry and W. J. Goux, *J. Magn. Reson.*, 2015, **257**, 15–23.

4 M. J. Frisch, G. W. Trucks, H. B. Schlegel, G. E. Scuseria, M. A. Robb, J. R. Cheeseman, G. Scalmani, V. Barone, G. A. Petersson, H. Nakatsuji, X. Li, M. Caricato, A. V. Marenich, J. Bloino, B. G. Janesko, R. Gomperts, B. Mennucci, H. P. Hratchian, J. V. Ortiz, A. F. Izmaylov, J. L. Sonnenberg, F. Ding, F. Lipparini, F. Egidi, J. Goings, B. Peng, A. Petrone, T. Henderson, D. Ranasinghe, V. G. Zakrzewski, J. Gao, N. Rega, G. Zheng, W. Liang, M. Hada, M. Ehara, K. Toyota, R. Fukuda, J. Hasegawa, M. Ishida, T. Nakajima, Y. Honda, O. Kitao, H. Nakai, T. Vreven, K. Throssell, J. A. Montgomery Jr., J. E. Peralta, F. Ogliaro, M. J. Bearpark, J. J. Heyd, E. N. Brothers, K. N. Kudin, V. N. Staroverov, T. A. Keith, R. Kobayashi, J. Normand, K. Raghavachari, A. P. Rendell, J. C. Burant, S. S. Iyengar, J. Tomasi, M. Cossi, J. M. Millam, M. Klene, C. Adamo, R. Cammi, J. W. Ochterski, R. L. Martin, K. Morokuma, O. Farkas, J. B. Foresman and D. J. Fox, *Gaussian 16 Rev. C.01*, 2016.

5 P. J. Stephens, F. J. Devlin, C. F. Chabalowski and M. J. Frisch, *J. Phys. Chem.*, 1994, **98**, 11623–11627.

6 S. Grimme, J. Antony, S. Ehrlich and H. Krieg, *J. Chem. Phys.*, 2010, **132**, 154104.

7 F. Weigend and R. Ahlrichs, *Phys. Chem. Chem. Phys.*, 2005, **7**, 3297–3305.

8 T. Lu, *J. Chem. Phys.*, 2024, **161**, 082503.

9 T. Lu and F. W. Chen, *J. Comput. Chem.*, 2012, **33**, 580–592.
